# Design and Synthesis of Hydroxamate-Based Matrix Metalloproteinase-2 Inhibitors for Anti-Photoaging

**DOI:** 10.4014/jmb.2412.12027

**Published:** 2025-02-10

**Authors:** Jin Young Lee, Geunhyuk Jang, Yung Hyup Joo, Joonho Choi, Dong-Woo Lee, Jae Won Yoo

**Affiliations:** 1 Amorepacific Research and Innovation Center, Yongin, Gyeonggi-do 17074, Republic of Korea; 2 Department of Biotechnology, Yonsei University, Seoul 03722, Republic of Korea; 3 Graduate Program in Biomaterials Science & Engineering, Yonsei University, Seoul 03722, Republic of Korea

**Keywords:** Matrix metalloproteinases (MMP), MMP-2, hydroxamate derivatives, photoaging, collagen degradation

## Abstract

Matrix metalloproteinases-2 (MMP-2) is crucial for collagen degradation at the dermal-epidermal junction, contributing to skin aging and photoaging. This study presents a series of hydroxamate-based inhibitors selectively targeting MMP-2. Through structure-activity relationship analysis, we systematically modified the *N*-arylsulfonyl group and amino acid backbone to enhance MMP-2 selectivity. Compounds 1ad, 1af, and **4an** showed strong MMP-2 inhibition, with 1ad demonstrating nanomolar-level selectivity. Zymogram assays revealed 30-60% MMP-2 activity reduction, while gene expression analysis confirmed post-transcriptional inhibition. These hydroxamate-based inhibitors are promising candidates for anti-photoaging applications, combining potent MMP-2 inhibition with simplified synthesis, supporting their potential for large-scale cosmetic formulations aimed at improving skin firmness and reducing wrinkles.

## Introduction

Skin aging, particularly wrinkle formation and loss of elasticity, results from both intrinsic and extrinsic factors. Intrinsic aging arises from the natural decline in cellular processes, while extrinsic factors, such as ultraviolet (UV) radiation, pollution, and oxidative stress, accelerate the breakdown of key skin components, especially collagen fibers [1-3]. Matrix metalloproteinases (MMPs), a family of zinc-dependent endopeptidases, play a pivotal role in this degradation by breaking down extracellular matrix (ECM) components, particularly collagen, which is vital for maintaining skin structure [4]. MMPs also participate in tissue remodeling and are implicated in various pathological conditions [5].

Among the MMPs, MMP-1 is primarily responsible for degrading type I collagen, a major structural component of the dermis [6]. UV-induced activation of the activator protein-1 (AP-1) pathway upregulates MMP-1, accelerating collagen breakdown and leading to visible signs of photoaging, such as wrinkles and fine lines [7, 8]. Anti-aging treatments, including retinoids and peptides, aim to reduce collagen degradation by targeting MMP-1 [9-11]. Pretreatment with tretinoin, for example, inhibits AP-1 activation and subsequent MMP upregulation following UV exposure [12, 13]. Several pan-MMP inhibitors, such as Batimastat, marimastat, and ilomastat, have been developed [14, 15]. However, these compounds face limitations, including structural complexity, synthetic challenges [16], and poor skin permeability [17], making them unsuitable for large-scale cosmetic applications.

Recent advancements in gene expression analysis, including Genome-Tissue Expression (GTEx) data, have highlighted the increasing role of MMP-2 in skin aging, particularly in photoaging [8, 18, 19]. MMP-2 degrades type IV collagen at the dermal-epidermal junction (DEJ), which is critical for maintaining the barrier between the dermis and epidermis [20]. Damage to the DEJ contributes to deep wrinkle formation and skin thinning, hallmark signs of aged skin [8]. Unlike MMP-1, which primarily affects the dermis, MMP-2’s involvement in the DEJ highlights its role in the progressive decline of skin integrity. Given the complexity of UV-induced MMP-2 upregulation in photoaged skin, targeting the inhibition of MMP-2 offers a promising strategy for effective anti-photoaging interventions. This approach necessitates the development of novel MMP-2-specific inhibitors with simplified structures and scalable synthetic pathways.

Hydroxamate-based inhibitors, known for their strong zinc-binding affinity and favorable peptidomimetic backbone, present a promising approach for selective MMP inhibition, with potential commercial applications [16, 21]. This study focuses on the design and synthesis of a series of hydroxamate derivatives of *N*-arylsulfonyl amino acids 1, specifically targeting MMP-2 over MMP-1. By exploiting structural differences between the S1’ pockets of MMP-1 and MMP-2, these inhibitors were optimized for MMP-2 selectivity. The larger, more hydrophobic S1’ pocket of MMP-2 allows for fine-tuning of inhibitory activity through modifications to the hydroxamate derivatives [22, 23]. Using molecular docking simulations and *in vitro* assays, this study evaluates the efficacy of these compounds and explores their potential as commercially viable treatments for anti-photoaging, while optimizing inhibitory profiles through simple synthetic methods.

## Materials and Methods

### Synthesis of Hydroxamate Derivatives of *N*-arylsulfonyl Amino Acids

All chemicals used for the synthesis were purchased from Sigma Aldrich (USA). *N*-Arylsulfonyl amino acids **3** were synthesized through two-step processes (Fig. S1A). Initially, amino acid **2** (5.0 mmol) was dissolved in tetrahydrofuran (THF, 10 ml) and combined with 1 M aqueous sodium carbonate solution (2.3 eq.). Arylsulfonyl chloride (6.0 mmol) was then added dropwise at 0°C, and the reaction mixture was stirred for 1 h. After extraction with diethyl ether (2 × 20 ml), the aqueous phase was acidified to pH 3 using 3 N HCl, then extracted with ethyl acetate (2 × 20 ml). The combined organic layers were dried with MgSO_4_ and concentrated under reduced pressure using a rotary evaporator. Purification of the crude product was performed via silica gel column chromatography to yield *N*-arylsulfonyl amino acid **3**.

For hydroxamate formation, *N*-arylsulfonyl amino acid **3** (3.0 mmol) was dissolved in THF (6 ml) and *N*-methylmorpholine (363 μl, 3.3 mmol) was added. Ethyl chloroformate (316 μl, 3.3 mmol) was introduced dropwise at 0°C, and the reaction mixture was stirred for 30 minutes. After filtering, the filtrate was added dropwise to hydroxylamine hydrochloride (313 mg, 4.5 mmol) and triethylamine (627 μl, 4.5 mmol) in DMF (4 ml) and stirred for 30 min at room temperature. DMF was evaporated under vacuum, and the reaction mixture was extracted with ethyl acetate (3 × 10 ml). The organic layers were concentrated under reduced pressure and purified by silica gel column chromatography to yield the desired hydroxamate derivative **1**.

Reactions were monitored using thin-layer chromatography (TLC) using Merck silica gel 60 F254 glass plates (0.25-mm thickness), visualized under UV light (254 nm). Column chromatography was performed on kieselgel 60 (70-230 mesh) silica gel. Nuclear magnetic resonance (NMR) spectra were recorded on spectrometers at 500 MHz for ^1^H and 100 MHz for ^13^C in DMSO, with chemical shifts (ppm) referenced to solvent signals (^1^H: DMSO, 2.50 ppm; ^13^C: DMSO, 39.52 ppm) (Supplementary Tables). NMR data were reported as: chemical shift (parts per million, ppm), multiplicity (s = singlet, d = doublet, t = triplet, q = quartet, qui*n* = quintet, sex = sextet, m = multiplet, br = broad signal), coupling constant (Hz), and integration. High-resolution mass spectra were obtained using electron ionization (EI), the chemical ionization (CI), or fast atom bombardment (FAB), analyzed by magnetic sector mass analyzer or LTQ Orbitrap Spectrometry (Thermo Fisher Scientific, USA).

### *In vitro* MMP Inhibition Assay

Hydroxamate derivatives of *N*-arylsulfonyl amino acids **1** were evaluated for MMP-1 and MMP-2 inhibition using assay kits (catalog #ab139443 for MMP-1 and #ab139446 for MMP-2) (Abcam, UK) according to the manufacturer’s instructions. Their MMP inhibitory effects were compared with those of ARP-101 (**5**), a known selective MMP-2 inhibitor. Test inhibitors were incubated with MMP enzymes and assay buffer at 37ºC for 30 min. Enzyme reaction was initiated by adding substrate, and MMP activity was measured by monitoring optical density (OD) at 412 nm for 20 min at 1-min intervals. The residual activity (%) was calculated as: residual activity = (V_inhibitor_/V_control_) × 100. The reaction velocity (V) in OD/min were determined by the slope of a line fit to the linear portion of the measured values. IC_50_ values were determined for compounds showing inhibitory activity against MMP-1 (500 nM) and MMP-2 (50 nM), and experiments were performed in duplicate.

### Structure-Activity Relationship (SAR) Analysis

For molecular docking simulation, the three-dimensional structures of MMP-1 (PDB ID: 3SHI) and MMP-2 (1QIB) were obtained from the RCSB Protein Data Bank (PDB). The structures of hydroxamate derivatives of *N*-arylsulfonyl amino acids 1 were prepared using ChemDraw software. Docking simulations were performed using AMDock (Assisted Molecular Docking, https://github.com/Valdes-Tresanco-MS), with AutoDock4 under the Autodock4Zn force field to preserve the zinc metal charge [24-26]. The three-dimensional structures of the MMPs and ligands were optimized using Open Babel [27] and PDB2PQR with AMBER forcefield [28], and the search space was defined using AutoLigand according to the default setting of AMDock program [29]. The search space coordinates for MMP-1 (3SHI) were X = 10.6, Y = -30.0, Z = 0.0, and for MMP-2 (PDB ID: 1QIB), X = 74.6, Y = 26.9, Z = 17.2. The box size used for docking was 30×30×30 with each point set to 0.375 Å. The predicted poses and affinity energies was calculated and displayed using AMDock. PyMOL in AMDock package was utilized to visualize the obtained results [30].

### Cell Culture

Normal human fibroblast (NHF) and HaCaT keratinocyte cells were cultured in Dulbecco's Modified Eagles Medium (DMEM) (Welgene Inc., Republic of Korea) supplemented with 10% fetal bovine serum (FBS) (Gibco, USA) and 100 U/ml potassium penicillin and 100 mg/ml streptomycin sulfate (Lonza Ltd., Switzerland). Cells were incubated at 37ºC with 5% CO_2_ (Thermo Fisher Scientific).

### Zymogram Assay

Conditioned media containing MMP-2 from NHF and HaCaT cell cultures were loaded onto SDS-polyacrylamide gels containing gelatin (Invitrogen #ZY00100BOX) (Thermo Fisher Scientific). After electrophoresis, the gels were washed in renaturing buffer and incubated in developing buffer for 20 h at 37°C with or without MMP inhibitors. Gels were stained with SimplyBlue™ SafeStain (Invitorgen #LC6065) (Thermo Fisher Scientific) and scanned. MMP activity appeared as clear bands, and quantification was performed by densitometry using Image J (https://imagej.net/ij/docs/faqs.html) [31]. The gelatin degradation (%) was calculated as: gelatin degradation = (PV_inhibitor_/PV_control_) × 100. The pixel values (PV) for the clear bands were determined by the pixel intensity in the same area.

### Gelatin Degradation Assay

Gelatin digestion was assessed using the QCM™ Gelatin Invadopodia Assay (#ECM670) (Sigma-Aldrich, USA). Plates were first coated with poly-L-lysine, activated with glutaraldehyde, and then coated with fluorescein-labelled gelatin. NHF cells were seeded onto the fluorescent gelatin-coated plates and incubated for 4 days. After incubation, the cells were fixed with formaldehyde and stained with DAPI for normalization based on cell number. Fluorescent gelatin levels were measured by detecting fluorescent intensity at an excitation wavelength of 492 nm and an emission wavelength of 518 nm using a Bio Tek Synergy H1 fluorescence reader (Agilent Technologies, USA). The residual gelatin (RG) was calculated by subtracting the fluorescent value of the wells with seeded cells from the values of wells without cells. This was normalized using the DAPI fluorescence to account for cell numbers per well. Gelatin degradation (%) was determined as: Gelatin degradation = (RG_inhibitor_/RG_control_) × 100.

### RNA Extraction and Reverse Transcription–Real-Time PCR

Total RNA from NHF and HaCaT cells was isolated using RNeasy Kits (#74104) (Qiagen, Germany) according to the manufacturer’s instructions. cDNA was synthesized using the SuperScript III First-Strand Synthesis System (#1808851) (Thermo Fisher Scientific). The levels of mRNA were quantified by real time PCR with an Applied Biosystem™ 7500 Fast Real-Time PCR system (Thermo Fisher Scientific) by normalizing to the expression of the housekeeping gene RPLP0. Primers used were as follows: MMP-1 and MMP-2 (TaqMan™ Gene Expression Assay ID: Hs00899658_m1, Hs01548727_m1)

### Wound Healing Assay

To evaluate *in vitro* wound closure, a cell scratch assay was performed using HaCaT cells. The cells were seeded to reach 70-80% confluence, and a scratch was created in the cell monolayer using a sterile pipette tip to simulate a wound. The cells were incubated, and images of the scratch area were captured at 1-hour intervals for 40 h using the JuLI stage imaging system (Nanoenteck, Republic of Korea). The images were analyzed to quantify wound closure over time.

## Results

### Structural Analysis and Design of Novel MMP Inhibitors Targeting MMP-2

**Fig. 1:**
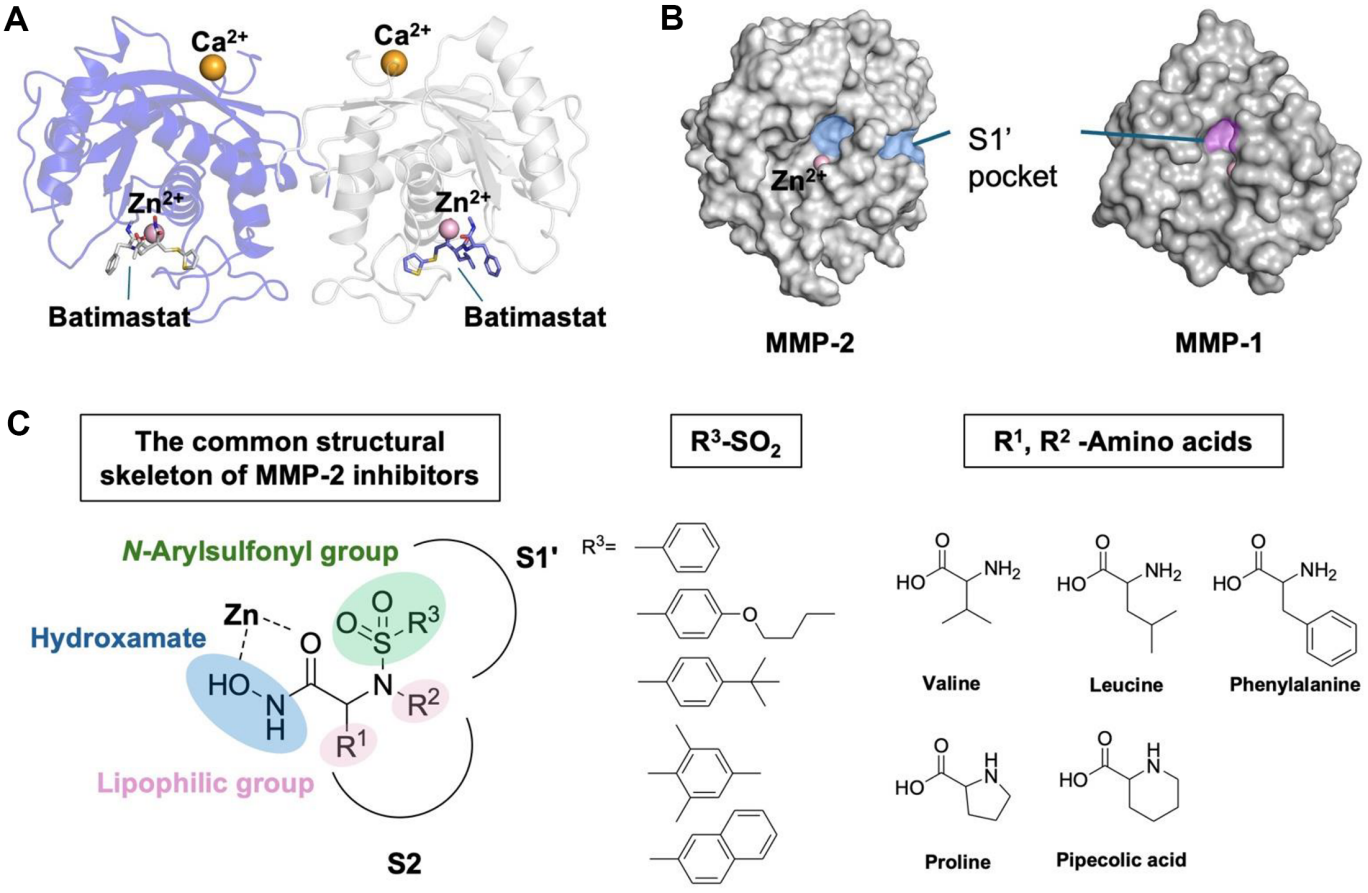
Structural analysis, design strategy, and synthesis of MMP-2 inhibitors. (**A**) Overall structure of MMP-1 (PDB ID: 1DTH) bound to a hydroxamate-based inhibitor; shown in front view (blue) and back view (grey) (**B**) Comparison of MMP-2 (PDB ID: 1QIB) and MMP-1 (PDB ID: 3SHI), highlighting essential binding domains, including the zinc-binding site and substrate-binding S1’ pocket, which are critical for selective inhibition. (**C**) Semi-rational design strategy for MMP-2 inhibitors, featuring hydroxamate-based zinc-binding groups (ZBGs) and selection of *N*-arylsulfonyl groups with varying lengths and bulkiness to enhance interactions within the hydrophobic S1' pocket of MMP-2.

A detailed structural analysis of the catalytic domains of MMP-1 and MMP-2, focusing on the zinc (Zn) ion-binding site and the substrate-binding S1’ pocket, revealed critical distinctions between these enzymes (Fig. 1A and 1B). The zinc ion at the catalytic site is essential for substrate degradation, making it a key target for inhibition. Zinc-binding groups (ZBGs), particularly hydroxamates, are effective in chelating the zinc ion, thereby blocking MMP activity. This inhibition mechanism is well-documented in established inhibitors (*e.g.*, Batimastat and CGS-27023A), which inhibit MMP-1 by binding within crystallized complexes. These insights informed a semi-rational design strategy aimed at developing selective MMP-2 inhibitors. Our design approach combined hydroxamate-based ZBGs with various *N*-arylsulfonyl groups to exploit the structural differences between the S1’ pockets of MMP-1 and MMP-2. The S1’ pocket of MMP-2 is notably larger and more hydrophobic than that of MMP-1, making it an ideal target for selective inhibition. The primary objective was to maximize MMP-2 inhibition while minimizing structural complexity to facilitate synthesis and scalability for potential commercial production.

To achieve this, we designed hydroxamate derivatives of *N*-arylsulfonyl amino acids 1 with a focus on cosmetic applications. The hydroxamates functioned as the zinc-binding moiety, effectively inhibiting the catalytic activity of MMPs. Various amino acids and *N*-arylsulfonyl groups were incorporated into a peptidomimetic backbone to optimize interactions within MMP-2’s larger, hydrophobic S1’ pocket. Bulky amino acids, such as valine, leucine, and phenylalanine, along with cyclic amino acids like proline and pipecolic acid, were selected for their compatibility with the S2 pocket (Fig. 1C). This rational design approach prioritized both steric and hydrophobic interactions to achieve selective MMP-2 inhibition while maintaining synthetic accessibility and scalability, making these compounds promising candidates for cosmetic applications targeting anti-photoaging treatment.

### Synthesis of Hydroxamate Derivatives of *N*-Arylsulfonyl Amino Acids

Based on the structural design, we synthesized a series of hydroxamate derivatives of *N*-arylsulfonyl amino acids **1** to develop selective inhibitors targeting MMP-2 over MMP-1. The synthesis strategy involved *N*-arylsulfonylation of amino acids with bulky side chains, such as valine, leucine, and phenylalanine, chosen to optimize binding within the active sites of MMP-1 and MMP-2 (Fig. 1C and S1). Cyclic amino acids, including proline and pipecolic acid, were also incorporated to compare their inhibitory effects with those of bulky, linear side-chain amino acids. The *N*-arylsulfonylation reactions were performed in an aqueous two-phase system using various *N*-arylsulfonyl chlorides (Fig. S1A, Scheme 1). The resulting *N*-arylsulfonyl carboxylic acids **3** were then converted into hydroxamates **1** via activation with ethyl chloroformate, followed by reaction with hydroxylamine hydrochloride and triethylamine (TEA) in the presence of *N*-methylmorpholine.

Substrates containing sterically hindered amino acids or bulky *N*-arylsulfonyl groups, such as those listed in entries **b**, **j**, and **ah**, generally yielded lower products. Notably, reactions involving 4-acetylbenzenesulfonyl pipecolic acid (**3an**) unexpectedly resulted in the formation of oxime **4an**, even with only 1.0 equivalent of hydroxylamine hydrochloride, suggesting that the reactivity of the acetyl group contributed to additional product modifications (Fig. S1A, Scheme 2). Overall, the synthetic pathway followed consistent steps of *N*-arylsulfonylation, followed by conversion to hydroxamates using ethyl chloroformate, *N*-methylmorpholine, and hydroxylamine hydrochloride.

In total, 40 compounds were successfully synthesized, including the unexpected oxime derivative **4an** (Table S1). Yields for these compounds varied significantly, ranging from 6.1% to 76.6%, with certain compounds, such as **1h**, **1ad**, and **1af**, showing both reasonable yields and potent inhibitory activity against MMP-2 (Table S2).

### *In vitro* MMP Inhibition Assay

**Fig. 2:**
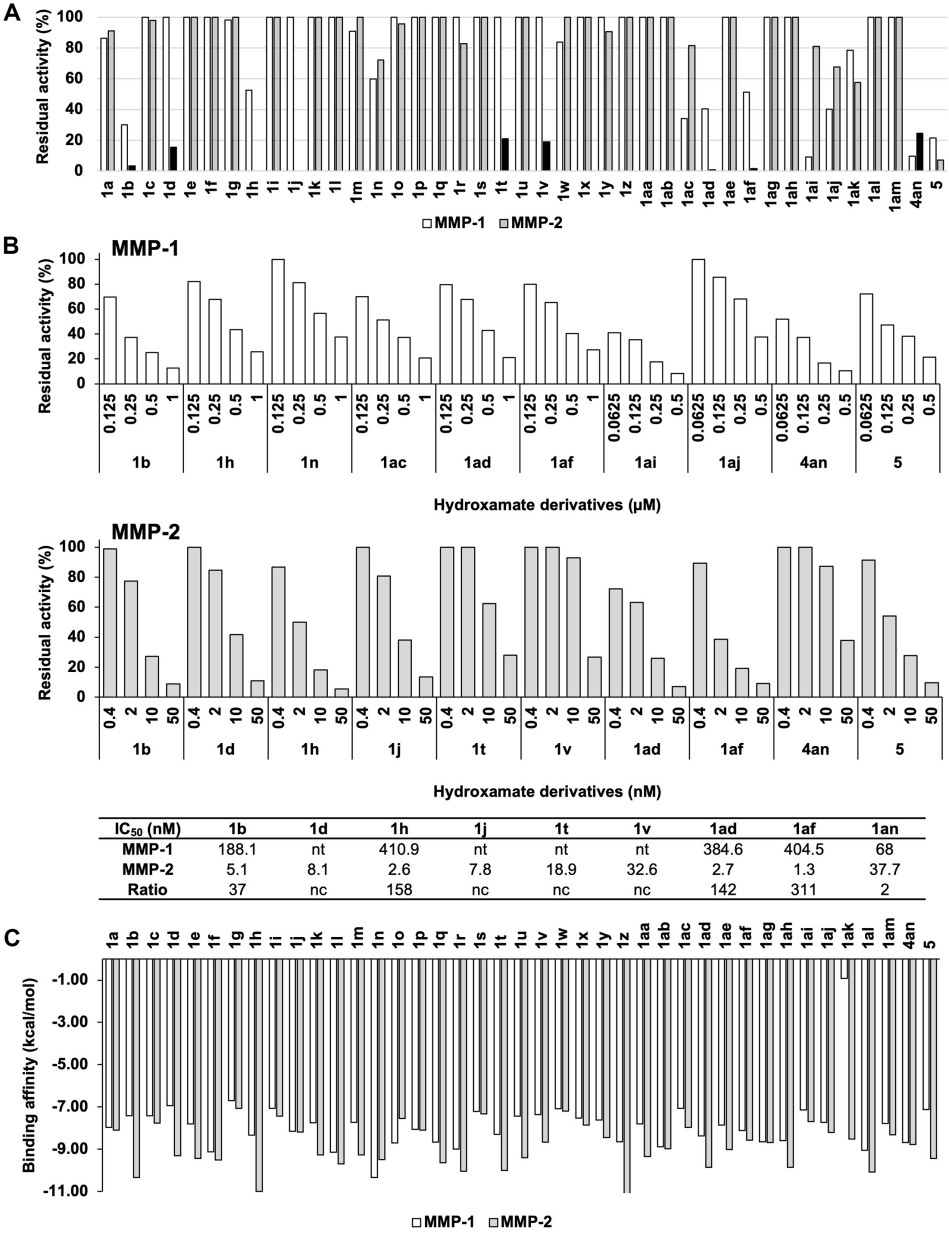
*In vitro* inhibition assay of synthesized hydroxamate derivatives of *N*-arylsulfonyl amino acids against MMP-1 and MMP-2. (**A**) Inhibition profiles of synthesized compounds, tested at concentrations of 500 nM for MMP-1 and 50 nM for MMP-2, reveal greater sensitivity of MMP-2 to inhibition. Experiments were conducted in duplicate. (**B**) Concentration-dependent inhibition and apparent IC_50_ values for selected compounds, demonstrating MMP-2 selectivity. Compounds such as **1b, 1d, 1h, 1j, 1ad** and **1af** show potent inhibition of MMP-2, with IC_50_ values below 10 nM, indicating their potential as selective MMP-2 inhibitors. (**C**) Binding affinity values (kcal/mol) for hydroxamate derivatives of *N*-arylsulfonyl amino acids **1**, determined through molecular docking simulations with the catalytic zinc ion in the S1’ pocket of MMP-1 and MMP-2. Compounds with extended *N*-arylsulfonyl chains, such as **1ad** and **1af**, displayed stronger affinity for MMP-2, reflected by lower binding energy values, suggesting tighter binding and higher inhibition potency.

The synthesized hydroxamate derivatives of *N*-arylsulfonyl amino acids **1** were evaluated for their inhibitory effects on MMP-1 and MMP-2 by measuring the degradation of synthetic MMP substrates. Initial *in vitro* inhibition assays demonstrated that these compounds exhibited significantly stronger inhibition of MMP-2 compared to MMP-1, with effective MMP-2 inhibition observed at concentrations below 50 nM, whereas MMP-1 inhibition required concentrations exceeding 500 nM. Key compounds, including **1b, 1d, 1h, 1j, 1t, 1v, 1ad**, and **1af**, selectively inhibited MMP-2 over MMP-1, underscoring the impact of structural modifications. In contrast, oxime **4an** exhibited dual inhibition, effectively targeting both MMP-1 and MMP-2 (Fig. 2A). These results highlight the potential of these compounds as targeted MMP-1 and MMP-2 dual inhibitors for anti-photoaging applications.

Concentration-dependent inhibition studies on selected compounds provided effective inhibitory concentration (IC_50_) values and selectivity ratios (Fig. 2B). Molecular docking simulations supported these findings, predicting stronger binding interactions between the hydroxamate derivatives and the catalytic sites of MMP-2 compared to MMP-1. Compounds such as **1ad** exhibited higher binding affinity for MMP-2 (-9.86 kcal/mol) than for MMP-1 (-8.37 kcal/mol), suggesting that longer, more hydrophobic *N*-arylsulfonyl groups are more compatible with the S1’ pocket of MMP-2 (Fig. 2C). These molecular insights explain the selective inhibition observed in the *in vitro* assays, indicating that the compounds were structurally optimized for MMP-2 inhibition.

### Structure-Activity Relationship (SAR) Analysis

**Fig. 3:**
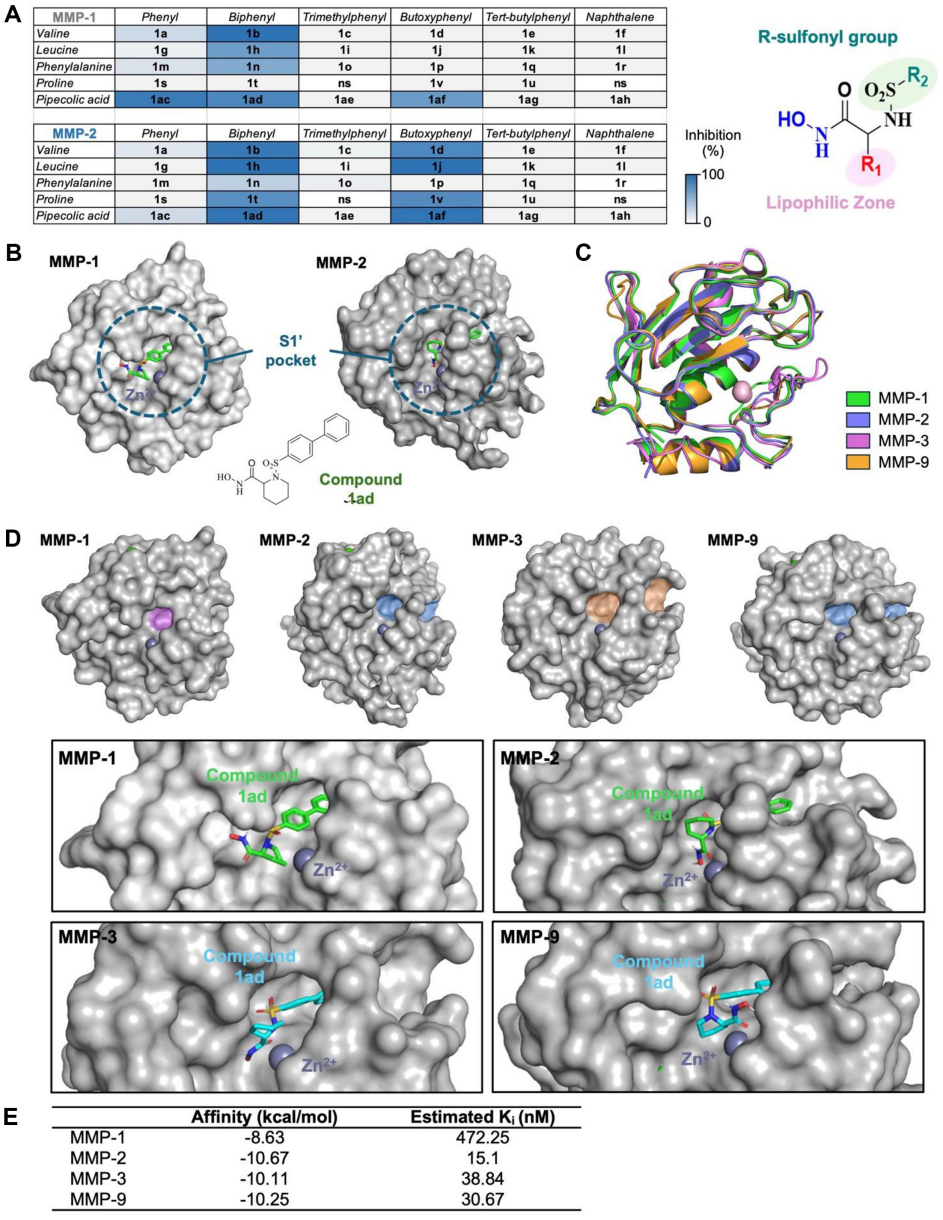
Comparison of synthesized compounds for various MMPs. (**A**) Comparison of MMP-1 (500 nM) and MMP- 2 (50 nM) inhibition according to amino acid and sulfonyl group combinations. (**B**) Molecular docking poses of compound **1ad** in MMP-1 and MMP-2, showing interactions with the catalytic zinc ion in the S1’ pocket. The docking simulations underscore the enhanced binding affinity of compounds like **1ad** for MMP-2 over MMP-1. (**C**) Structural alignment of MMP-2 with other matrix metalloproteinases (MMP-1, MMP-3, and MMP-9), emphasizing structural differences in the S1' pocket and zincbinding regions that inform selective inhibition strategies. MMP-1 (PDB ID: 3SHI) is shown in yellow, MMP-2 (PDB ID: 1QIB) in cyan, MMP-3 (PDB ID: 2D1O) in magenta, and MMP-9 (PDB ID: 5I12) in green. (**D**) (upper panel) Structural variations in the S1’pocket shape across MMP catalytic domains, including MMP-1 (PDB ID: 3SHI), MMP-2 (PDB ID: 1QIB), MMP-3 (PDB ID: 2D1O), and MMP-9 (PDB ID: 5I12), illustrating shape and depth differences. S1’ pockets of these proteins are color-coded by depth: magenta for shallow, blue for intermediate, and orange for deep, with gray surfaces for overall pocket structure. (bottom panel) Compound **1ad** is shown in green or cyan with gray surfaces, and zinc ions are depicted as dark gray spheres. (**E**) Binding affinity of synthesized compound **1ad** in various MMPs in molecular docking simulations.

The SAR analysis revealed that steric hindrance and hydrophobicity of the *N*-arylsulfonyl group were critical factors in modulating the inhibitory activity, particularly for MMP-2. Too bulky *N*-arylsulfonyl groups, such as *N*-4-*tert*-butylphenylsulfonyl, *N*-2,4,6-trimethylphenylsulfonyl, and *N*-4-adamantylphenylsulfonyl, showed reduced inhibition of both MMP-1 and MMP-2, likely due to poor accommodation within the narrow S1’ pocket (Fig. 1B). In contrast, compounds featuring the *N*-4-butoxyphenylsulfonyl group (**1d, 1j, 1af**) demonstrated strong selectivity for MMP-2, suggesting that longer, more flexible side chains better fit the larger, hydrophobic S1’ pocket of MMP-2 (Fig. 3A).

The amino acid backbone also significantly influenced selectivity. Bulky amino acids, such as phenylalanine and valine, enhanced MMP-2 selectivity, whereas proline-containing derivatives (*e.g.*, **1t**) exhibited weaker MMP-1 inhibition, possibly due to the cyclic structure of proline limiting optimal binding within the MMP-1 pocket (Fig. 3A). Compounds with *N*-(4-phenyl)phenylsulfonyl groups (*e.g.*, **1b, 1h, 1ad**) showed weak MMP-1 inhibition, while phenylsulfonyl derivatives generally displayed reduced inhibition for both MMP-1 and MMP-2. Interestingly, phenylalanine-containing derivatives (*e.g.*, **1n, 1p**) exhibited lower inhibitory activity against MMP-2 compared to other amino acids, regardless of the presence of *N*-(4-phenyl)phenylsulfonyl or *N*-4-butoxyphenylsulfonyl groups (Fig. 3A). For the same *N*-arylsulfonyl group, pipecolic acid derivatives generally showed higher inhibitory activity against MMP-1 and MMP-2 compared to proline derivatives. While proline derivatives, such as **1t**, showed reduced inhibition, pipecolic acid derivatives (**1ac, 1af**) maintained MMP-1 inhibition when combined with phenylsulfonyl or *N*-4-butoxyphenylsulfonyl groups (Fig. 3A). This suggests that the pipecolic acid backbone plays a notable effect on modulating inhibitory potential against MMP-1.

Molecular docking simulations further elucidated the binding affinities of the inhibitors within the S1’ pocket of MMP-2. Compounds with extended *N*-arylsulfonyl chains, such as **1ad**, exhibited strong binding affinity for MMP-2, effectively fitting into its hydrophobic zones while showing lower affinity for MMP-1 (Fig. 3B). Comparative analysis of the S1’ pockets among MMP-1, MMP-2, MMP-3, and MMP-9 highlighted spectific structural adaptations in MMP-2 that enable selective inhibition by these hydroxamate derivatives (Fig. 3C and 3D). Calculated binding affinities further confirmed this selectivity, with compound **1ad** showing a stronger affinity for MMP-2 (-10.67 kcal/mol) compared to other MMP isoforms (Fig. 3E).

In summary, the MMP inhibition assays and SAR analysis identified several promising candidates for selective MMP-2 inhibition. These findings indicate the importance of optimizing *N*-arylsulfonyl groups and amino acid backbones to achieve high MMP-2 selectivity while minimizing MMP-1 inhibition. These selective MMP-2 inhibitors present strong candidates for further development in anti-photoaging applications.

### Biological Validation and Functional Evaluation of MMP-2 Inhibitors

**Fig. 4:**
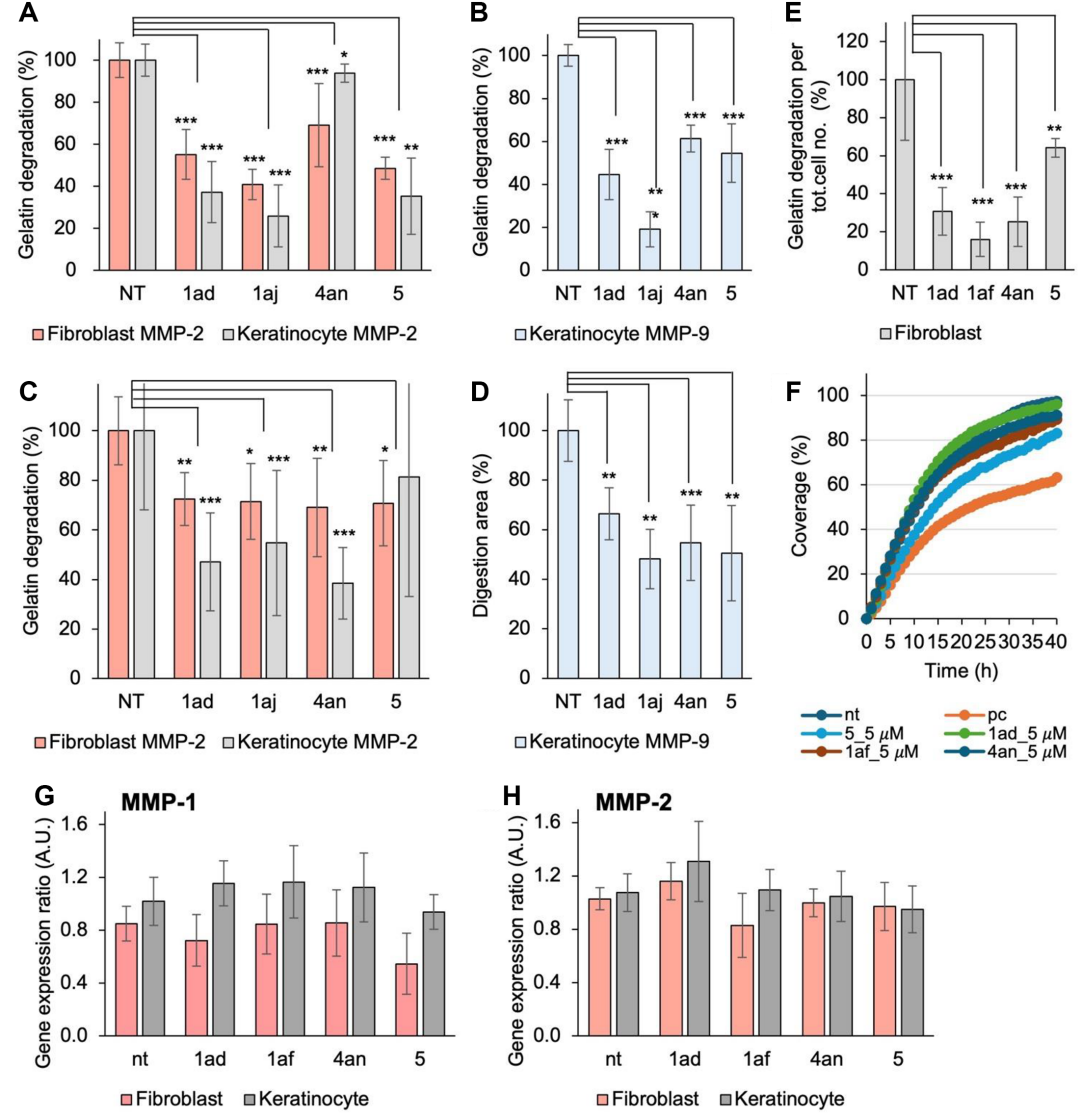
Gelatin digestion and gene expression analysis of MMP-2 and MMP-9 inhibitors. (**A**) Zymogram showing MMP-2 inhibition in conditioned media from normal human fibroblast (**NHF**) and HaCaT cells treated with 50 nM candidate compounds during the developing phase. Significance was assessed using JMP software with the Kruskal-Wallis test, followed by post-hoc analysis using the Pairwise Wilcoxon Test (***p < 0.001, **p < 0.01). (**B**) Zymogram showing MMP-9 inhibition in HaCaT cell conditioned media treated with 50 nM candidate compounds, performed similarly to (a) but only in keratinocytes. (Significance was assessed using JMP software with the Kruskal-Wallis test, followed by post-hoc analysis using the Pairwise Wilcoxon Test (***p < 0.001, **p < 0.01). (**C**) Zymogram analysis of MMP-2 activity in conditioned media from NHF and HaCaT cells after 48-h treatment with 500 nM candidate compounds, demonstrating sustained inhibition. Significance was assessed using JMP software with the Kruskal-Wallis test, followed by post-hoc analysis using the Pairwise Wilcoxon Test (***p < 0.001, **p < 0.01). (**D**) Zymogram analysis of MMP-9 activity in HaCaT cells after 48-h treatment with 500 nM candidate compounds, following the same experimental setup as (**C**) but only in keratinocytes. Zymogram assays were conducted in duplicate. Significance was assessed using JMP software with the Kruskal-Wallis test, followed by post-hoc analysis using the Pairwise Wilcoxon Test (***p < 0.001, **p < 0.01). (**E**) Inhibition of fluorescent gelatin degradation by NHF cells cultured with candidate compounds at 1 μM for 4 days on a fluorescent-gelatin-coated plate, performed in triplicate. Significance was assessed using JMP software with the Kruskal-Wallis test, followed by post-hoc analysis using the Pairwise Wilcoxon Test (***p < 0.001, **p < 0.01). (**F**) Wound closure assay of HaCaT cells treated with candidate compounds at 5 μM for 40 hours, evaluating the impact on cell migration and wound healing. (**G**) Gene expression analysis of MMP-1 and (**H**) MMP-2 in NHF and HaCaT cells treated with candidate compounds at 5 μM for 24 hours, indicating no significant changes in expression levels. Statistical significance was assessed using the Kruskal-Wallis test followed by the Pairwise Wilcoxon Test (*n* = 3).

The inhibitory efficacy of selected MMP-2 inhibitors (**1ad, 1af**, and **4an**) was validated through a series of *in vitro* assays, including zymogram assays, gelatin degradation analysis, gene expression studies, and wound healing assays. Zymogram assays conducted on conditioned media from normal human fibroblast (NHF) and keratinocyte (HaCaT) cultures treated with 50 nM of the candidate inhibitors during the developing phase confirmed MMP-2 inhibition, with inhibition rates between 30% and 60% across all tested compounds (Fig. 4A). Further zymogram analysis on conditioned media from NHF and HaCaT cells treated with the 500 nM of the inhibitors for 48 hours also demonstrated MMP-2 inhibition (Fig. 4C). Additional zymogram analysis of keratinocyte-conditioned media demonstrated that structural modifications effectively inhibited both MMP-2 and MMP-9, supporting the specificity of these compounds **1ad, 1af**, and **4an** was comparable to ARP-101 (**5**), a known selective MMP-2 inhibitor (Fig. 4B and 4D).

The gelatin degradation assays further demonstrated the sustained inhibitory activity of these compounds under near-physiological conditions. NHF cells cultured on fluorescent-gelatin-coated plates and treated with 1 μM of the candidate compounds for four days showed significant reductions in gelatin degradation, indicating effective inhibition of extracellular matrix breakdown by MMP-2. Quantification of gelatin degradation per cell revealed statistically significant reductions for **1ad, 1af** and **4an** (*p* < 0.001) and for ARP-101 (**5**) (*p* < 0.01), indicating the potential of compounds for long-term inhibition of MMP-mediated degradation (Fig. 4E).

A wound healing assay on HaCaT cells over a 40-hour period assessed the impact of MMP-2 inhibitors on cell migration and would closure. Wound closure rates in treated cells were similar to those in untreated controls, indicating that the inhibitors did not hinder natural skin regeneration (Fig. 4F). In contrast, the positive control (cell growth inhibition condition) showed significantly impaired wound healing. Although ARP-101 (5) strongly inhibited cell migration, compounds **1af** and **4an** caused only minor reductions, and **1ad** showed no inhibitory effect at 5 μM. This minimal effect on cell migration highlights the suitability of these compounds for cosmetic formulations targeting anti-photoaging, where preserving skin repair mechanisms is essential.

To explore the mechanism of inhibition, RT-qPCR analysis was performed to assess MMP-1 and MMP-2 gene expression in NHF and HaCaT cells treated with 5 μM of the inhibitors for 24 h. The results indicated no significant alterations in MMP-1 and MMP-2 mRNA levels, suggesting that the inhibitors act post-transcriptionally by directly targeting enzyme activity without influencing gene expression (Fig. 4G and 4H). This post-transcriptional mechanism offers the advantage of maintaining physiological skin homeostasis, achieving effective MMP-2 inhibition without interfering with cellular regulatory pathways.

In conclusion, the biological validation assays confirmed that compounds **1ad**, **1af**, and **4an** effectively inhibit MMP-2, with compound **4an** also demonstrating dual inhibition of MMP-1 and MMP-2. Although **4an** was less potent than the pan-MMP inhibitor Batimastat, its simpler structure and synthetic accessibility make it a promising candidate for anti-photoaging applications. The selective inhibition of MMP-2, combined with a post-transcriptional mechanism and minimal impact on wound healing, underscores the potential of these compounds as safe, effective agents for cosmetic formulations aimed at reducing collagen degradation and maintaining skin integrity.

## Discussion

This study focused on the design and synthesis of hydroxamate-based *N*-arylsulfonyl amino acid derivatives **1** to selectively inhibit MMP-2, a critical enzyme involved in collagen degradation in photoaged skin. The structure-activity relationship (SAR) analysis revealed that both the *N*-arylsulfonyl group and the amino acid backbone significantly influence selective inhibition of MMP-2 over MMP-1, a key goal for anti-photoaging applications where targeted MMP-2 inhibition is essential for preventing skin damage without disrupting normal collagen turnover. The hydroxamate group, known for its strong zinc-binding affinity, served as the core structure of these compounds due to its efficacy in chelating the catalytic zinc ion in MMPs, thereby blocking enzymatic activity. Molecular docking simulations confirmed that the most potent inhibitors displayed strong zinc interactions, essential for effectively disrupting the substrate degradation process (Figs. 2C and 3D). This binding was particularly favorable for MMP-2 due to the structural characteristics of its S1’ pocket, which is larger and more hydrophobic compared to MMP-1, allowing these inhibitors to achieve greater specificity (Fig. 1B).

To optimize MMP-2 selectivity, various *N*-arylsulfonyl groups with different steric and electronic properties were introduced (Fig. 1C). Compounds with longer, flexible groups, such as *N*-4-butoxyphenylsulfonyl and (4-phenyl)phenylsulfonyl, showed high selectivity for MMP-2. These groups were able to fit effectively into the larger and more hydrophobic S1’ pocket of MMP-2, enhancing binding affinity and inhibitory potency (Fig. 3A). In contrast, bulkier substituents like *N*-adamantylphenyl and naphthalene hindered inhibitory activity, likely due to steric hinderance that prevented optimal binding. Additionally, amino acid backbones, such as those derived from valine, leucine, and pipecolic acid, further enhanced selectivity by modulating hydrophobic and steric interactions within the binding pocket. Interestingly, proline-containing derivatives showed minimal MMP-1 inhibition, while pipecolic acid derivatives exhibited inhibition against both MMP-1 and MMP-2, suggesting the importance of flexibility on the backbone structure in targeting different MMPs.

Key compounds, such as **1ad, 1af**, and **4an**, exhibited potent MMP-2 inhibition in both *in vitro* and molecular docking assays (Figs. 2 and 3). Compound **1ad** and **1aj** showed selective inhibition of MMP-2 at nanomolar concentrations without significantly affecting MMP-1, validating its potential for targeted anti-photoaging applications. Compound **4an**, while showing dual inhibition of MMP-1 and MMP-2, presented a simpler structure with effective inhibition, making it a viable candidate for broader-spectrum MMP inhibition. Further supporting these findings, molecular docking simulations indicated that compounds like **1ad** had a stronger binding affinity for MMP-2 (-10.67 kcal/mol) compared to MMP-1 (-8.63 kcal/mol), highlighting the importance of the larger S1’pocket in MMP-2 to accommodate *N*-arylsulfonyl groups more effectively (Fig. 3B and 3E). It was successfully coordinated with the zinc ion in MMP-2, while achieving less favorable interactions in MMP-1. This molecular insight underscores the necessity of structural optimization to enhance selectivity and potency for MMP-2, reinforcing the design strategy employed in this study.

The straightforward synthetic routes and readily available starting materials make these inhibitors promising for large-scale production, particularly for commercial formulations targeting wrinkle reduction and skin firmness (Fig. S1). The selective MMP-2 inhibitors, such as **1ad** and **1af**, are well-suited for cosmetic applications aimed at reducing collagen degradation at the DEJ without disrupting normal collagen turnover. Future studies could focus on enhancing the stability and delivery of these compounds in formulations to improve bioavailability and skin penetration.

*In vitro* validation assays further confirmed the efficacy of selected compounds (*i.e.*, **1ad, 1af**, and **4an**), with zymogram assays showing MMP-2 inhibition rates of 30%-60% across all tested compounds (Fig. 4A and 4B). Conditioned media from NHF and HaCaT cells treated with the inhibitors demonstrated consistent MMP-2 inhibition, validating their potential for sustained activity in biological environments (Fig. 4C and 4D). Additionally, gelatin degradation assays indicated prolonged inhibitor activity in an *in vivo*-like setting, further supporting the potential utility of these compounds for anti-aging treatments. RT-qPCR analysis showed no significant alterations in MMP-2 mRNA levels, suggesting that these inhibitors act at a post-transcriptional level by directly targeting enzyme activity rather than gene expression (Fig. 4G and 4H). This post-transcriptional inhibition is advantageous, as it maintains skin homeostasis while effectively inhibiting MMP-2 activity without interfering with cellular processes.

**Fig. 5:**
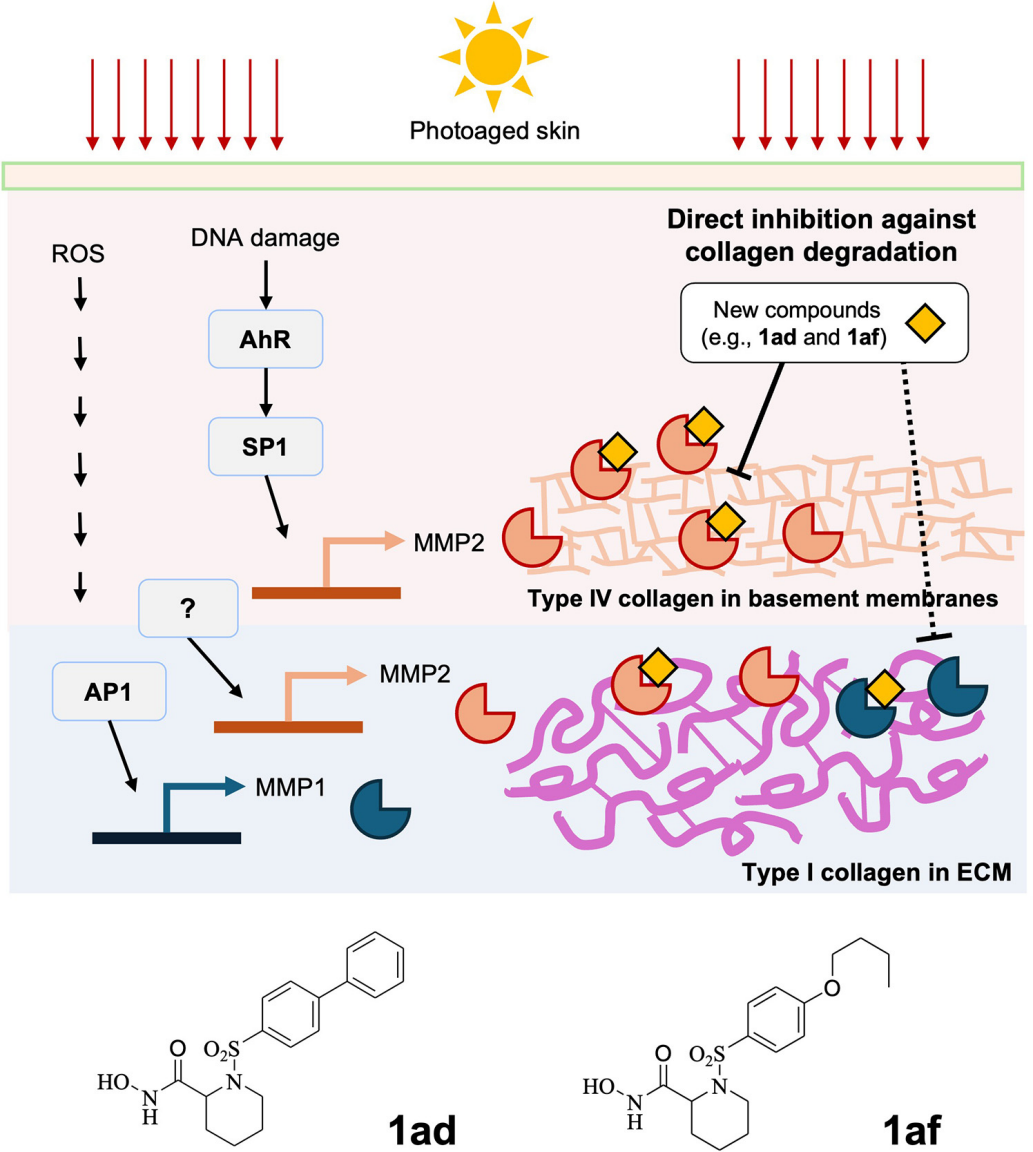
Proposed inhibition mechanism for compound 5ad in anti-photoaging. Compound **5ad** exhibits antiphotoaging effects through selective inhibition of MMP-2, which is upregulated by UV exposure. By inhibiting MMP-2, compound 5ad prevents the degradation of type IV collagen, thereby protecting the DEJ from UV-induced photo-damage. Additionally, it inhibits MMP-1 activity, reducing collagen breakdown within the dermis and preserving the structural integrity of the skin. This selective inhibition is anticipated to have minimal influence on the regulation of MMP gene expression within the skin, as its action is primarily through direct enzyme inhibition.

While MMP-1 is a primary contributor to collagen degradation in photoaged skin via UV-induced AP-1 activation [32], MMP-2’s role in degrading type IV collagen at the DEJ becomes increasingly significant with age [33-35]. This distinction emphasizes the need for selective MMP-2 inhibitors that can prevent further collagen breakdown while preserving MMP-1’s role in normal collagen turnover (Fig. 5). Compound **4an**, with its dual inhibition profile, provides an option for broader MMP inhibition, though it is less potent than the pan-MMP inhibitor Batimastat. Its simpler structure, however, offers advantages in terms of scalability and commercial viability.

## Conclusion

This study successfully synthesized a series of hydroxamate-based *N*-arylsulfonyl amino acid derivatives, demonstrating their effectiveness as selective MMP-2 inhibitors. SAR analysis highlighted how targeted modifications to the *N*-arylsulfonyl group and amino acid backbone modulate inhibitory activity, leading to the identification of both selective MMP-2 inhibitors (*e.g.*, **1ad, 1af**) and dual inhibitors (*e.g.*, **4an**). Given MMP-2's growing recognition as a contributor to photoaging, these compounds hold strong potential for use in anti-aging skincare formulations designed to target collagen degradation while preserving skin integrity (Fig. 5). Future studies should focus on further optimizing these inhibitors for improved potency and delivery in cosmetic applications.

## Supplemental Materials

Supplementary data for this paper are available on-line only at http://jmb.or.kr.
