# Supplementary material for: Design and Synthesis of Hydroxamate-Based Matrix Metalloproteinase-2 Inhibitors for Anti-Photoaging
Source: J Microbiol Biotechnol. 2025 Feb 10;35:e2412027. doi: 10.4014/jmb.2412.12027 (PMC11876007; doi:10.4014/jmb.2412.12027)

J. Microbiol. Biotechnol. 2025. 35: e2412027

https://doi.org/10.4014/jmb.2412.12027

Supplementary Tables and Figures

Design and Synthesis of Hydroxamate-Based Matrix Metalloproteinase-2 Inhibitors for Anti-Photoaging

Jin Young Lee[1,3], Geunhyuk Jang[1], Yung Hyup Joo[1], Joonho Choi[1], Dong-Woo Lee[2,3]*, and Jae Won Yoo[1,]*

[1]Amorepacific Research and Innovation Center, Yongin, Gyeonggi-do 17074, Republic of Korea

[2]Department of Biotechnology, Yonsei University, Seoul, 03722, Republic of Korea.

[3]Graduate Program in Biomaterials Science & Engineering, Yonsei University, Seoul, 03722, Republic of Korea.

Running title: Hydroxamate derivatives as MMP-2 inhibitors

*Corresponding authors:

Dong-Woo Lee,

E-mail: leehicam@yonsei.ac.kr

Jae Won Yoo,

Email: jwyoo@amorepacific.com

Table of Contents

Table **S1**. The synthesized hydroxamate derivates of *N*-sulfonyl amino acids: ***S3***

Table **S2**. Preparation and yield of hydroxamate derivates of *N*-sulfonyl amino acids: ***S4***

Figure **S1**. Synthesis pathway and structures of hydroxamate derivatives of *N*-arylsulfonyl amino acids:

S6

Characterization of the target compounds: ***S7***

1H-, 13C-NMR spectra and mass spectra of the target compounds: ***S13***

Table S1. The synthesized hydroxamate derivates of *N*-sulfonyl amino acids.

Entry	Compound Name
**1a**	*N*-hydroxy-3-methyl-2-(phenylsulfonamido)butanamide
**1b**	2-([1,1'-biphenyl]-4-sulfonamido)-*N*-hydroxy-3-methylbutanamide
**1c**	*N*-hydroxy-3-methyl-2-((2,4,6-trimethylphenyl)sulfonamido)butanamide
**1d**	2-((4-butoxyphenyl)sulfonamido)-*N*-hydroxy-3-methylbutanamide
**1e**	2-((4-(*tert*-butyl)phenyl)sulfonamido)-*N*-hydroxy-3-methylbutanamide
**1f**	*N*-hydroxy-3-methyl-2-(naphthalene-2-sulfonamido)butanamide
**1g**	*N*-hydroxy-4-methyl-2-(phenylsulfonamido)pentanamide
**1h**	2-([1,1'-biphenyl]-4-sulfonamido)-*N*-hydroxy-4-methylpentanamide
**1i**	*N*-hydroxy-4-methyl-2-((2,4,6-trimethylphenyl)sulfonamido)pentanamide
**1j**	2-((4-butoxyphenyl)sulfonamido)-*N*-hydroxy-4-methylpentanamide
**1k**	2-((4-(*tert*-butyl)phenyl)sulfonamido)-*N*-hydroxy-4-methylpentanamide
**1l**	*N*-hydroxy-4-methyl-2-(naphthalene-2-sulfonamido)pentanamide
**1m**	*N*-hydroxy-3-phenyl-2-(phenylsulfonamido)propanamide
**1n**	2-([1,1'-biphenyl]-4-sulfonamido)-*N*-hydroxy-3-phenylpropanamide
**1o**	*N*-hydroxy-3-phenyl-2-((2,4,6-trimethylphenyl)sulfonamido)propanamide
**1p**	2-((4-butoxyphenyl)sulfonamido)-*N*-hydroxy-3-phenylpropanamide
**1q**	2-((4-(*tert*-butyl)phenyl)sulfonamido)-N-hydroxy-3-phenylpropanamide
**1r**	*N*-hydroxy-2-(naphthalene-2-sulfonamido)-3-phenylpropanamide
**1s**	*N*-hydroxy-1-(phenylsulfonyl)pyrrolidine-2-carboxamide
**1t**	1-([1,1'-biphenyl]-4-ylsulfonyl)-*N*-hydroxypyrrolidine-2-carboxamide
**1u**	1-((4-(*tert*-butyl)phenyl)sulfonyl)-*N*-hydroxypyrrolidine-2-carboxamide
**1v**	1-((4-butoxyphenyl)sulfonyl)-*N*-hydroxypyrrolidine-2-carboxamide
**1w**	1-((4-fluorophenyl)sulfonyl)-*N*-hydroxypyrrolidine-2-carboxamide
**1x**	*N*-hydroxy-1-tosylpyrrolidine-2-carboxamide
**1y**	*N*-hydroxy-1-((4-propylphenyl)sulfonyl)pyrrolidine-2-carboxamide
**1z**	1-((4-(adamantan-1-yl)phenyl)sulfonyl)-*N*-hydroxypyrrolidine-2-carboxamide
**1aa**	1-((4-acetamidophenyl)sulfonyl)-*N*-hydroxypyrrolidine-2-carboxamide
**1ab**	1-((4-acetylphenyl)sulfonyl)-*N*-hydroxypyrrolidine-2-carboxamide
**1ac**	*N*-hydroxy-1-(phenylsulfonyl)piperidine-2-carboxamide
**1ad**	1-([1,1'-biphenyl]-4-ylsulfonyl)-*N*-hydroxypiperidine-2-carboxamide
**1ae**	*N*-hydroxy-1-(mesitylsulfonyl)piperidine-2-carboxamide
**1af**	1-((4-butoxyphenyl)sulfonyl)-*N*-hydroxypiperidine-2-carboxamide
**1ag**	1-((4-(*tert*-butyl)phenyl)sulfonyl)-*N*-hydroxypiperidine-2-carboxamide
**1ah**	*N*-hydroxy-1-(naphthalen-2-ylsulfonyl)piperidine-2-carboxamide
**1ai**	1-((4-fluorophenyl)sulfonyl)-*N*-hydroxypiperidine-2-carboxamide
**1aj**	*N*-hydroxy-1-tosylpiperidine-2-carboxamide
**1ak**	*N*-hydroxy-1-((4-propylphenyl)sulfonyl)piperidine-2-carboxamide
**1al**	1-((4-(adamantan-1-yl)phenyl)sulfonyl)-*N*-hydroxypiperidine-2-carboxamide
**1am**	1-((4-acetamidophenyl)sulfonyl)-*N*-hydroxypiperidine-2-carboxamide
**4an**	*N*-hydroxy-1-((4-(1-(hydroxyimino)ethyl)phenyl)sulfonyl)piperidine-2-carboxamide

Table S2. Preparation and yield of hydroxamate derivates of *N*-sulfonyl amino acids.

Entry	R^1	R^2	R^3	Yields	
				**3**	**1**
a	-	-H	Ph-	75.0 %	41.5 %
b			4-Ph-Ph-	38.6 %	22.0 %
c			2,4,6-Me_3-Ph-	45.2 %	35.6 %
d			4-BuO-Ph-	52.2 %	38.7 %
e			4-*tert*-Bu-Ph-	55.0 %	50.9 %
f			2-Naph-	33.2 %	61.0 %
g	-	-H	Ph-	52.9 %	47.0 %
h			4-Ph-Ph-	46.6 %	64.3 %
i			2,4,6-Me_3-Ph-	58.1 %	27.6 %
j			4-BuO-Ph-	67.9 %	13.8 %
k			4-*tert*-Bu-Ph-	76.2 %	23.4 %
l			2-Naph-	33.5 %	18.2 %
m	-CH_2Ph	-H	Ph-	54.4 %	71.0 %
n			4-Ph-Ph-	75.7 %	38.1 %
o			2,4,6-Me_3-Ph-	67.3 %	34.0 %
p			4-BuO-Ph-	81.2 %	52.9 %
q			4-*tert*-Bu-Ph-	82.1 %	60.4 %
r			2-Naph-	77.2 %	33.5 %
s	-$(CH_2)_3$-	-	Ph-	95.2 %	61.9 %
t			4-Ph-Ph-	44.8 %	70.7 %
u			4-*tert*-Bu-Ph-	73.6 %	84.4 %
v			4-BuO-Ph-	94.4 %	45.4 %
w			4-F-Ph-	67.9 %	37.2 %
x			4-Me-Ph-	92.3 %	56.1 %
y			4-Pr-Ph-	87.4 %	80.3 %
z			4-Adamantyl-	Quant.	76.6 %
aa			4-AcNH-Ph-	45.6 %	48.0 %
ab			4-Ac-Ph-	80.5 %	23.1 %
ac	-$(CH_2)_4$-	-	Ph-	63.6 %	46.1 %
ad			4-Ph-Ph-	44.9 %	45.6 %
ae			2,4,6-Me_3-Ph	45.3 %	29.4 %
af			4-BuO-Ph-	68.1 %	40.3 %
ag			4-*tert*-Bu-Ph-	49.5 %	60.2 %
ah			2-Naph-	19.3 %	37.7 %
ai			4-F-Ph-	54.7 %	33.8 %
aj			4-Me-Ph-	55.6 %	54.6 %

ak	4-Pr-Ph-	Quant.	31.8 %
al	4-Adamantyl-Ph	57.6 %	67.8 %
am	4-AcNH-Ph-	74.0 %	56.5 %

a

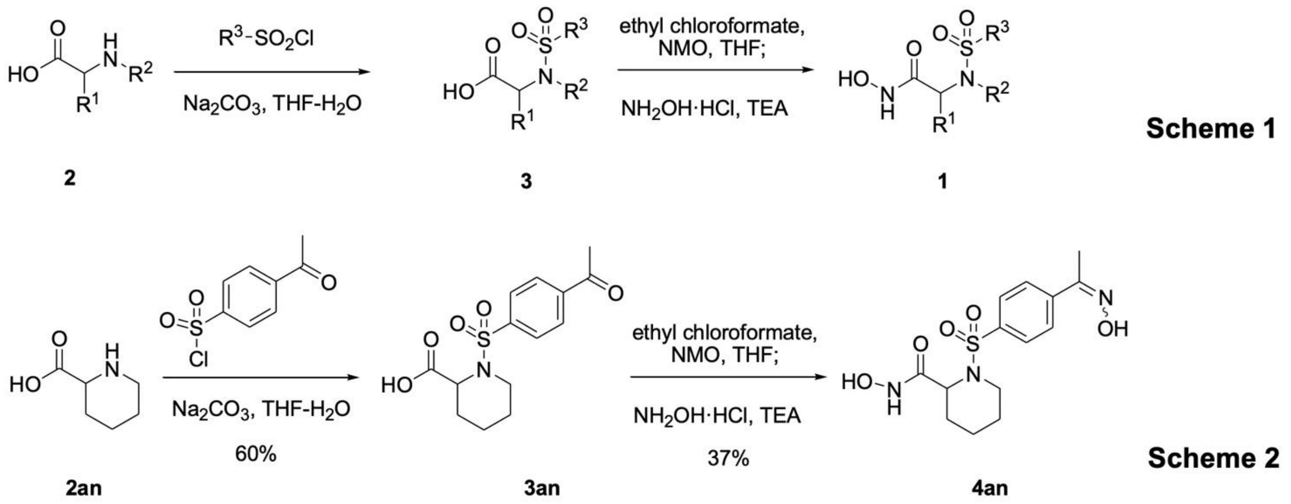

b

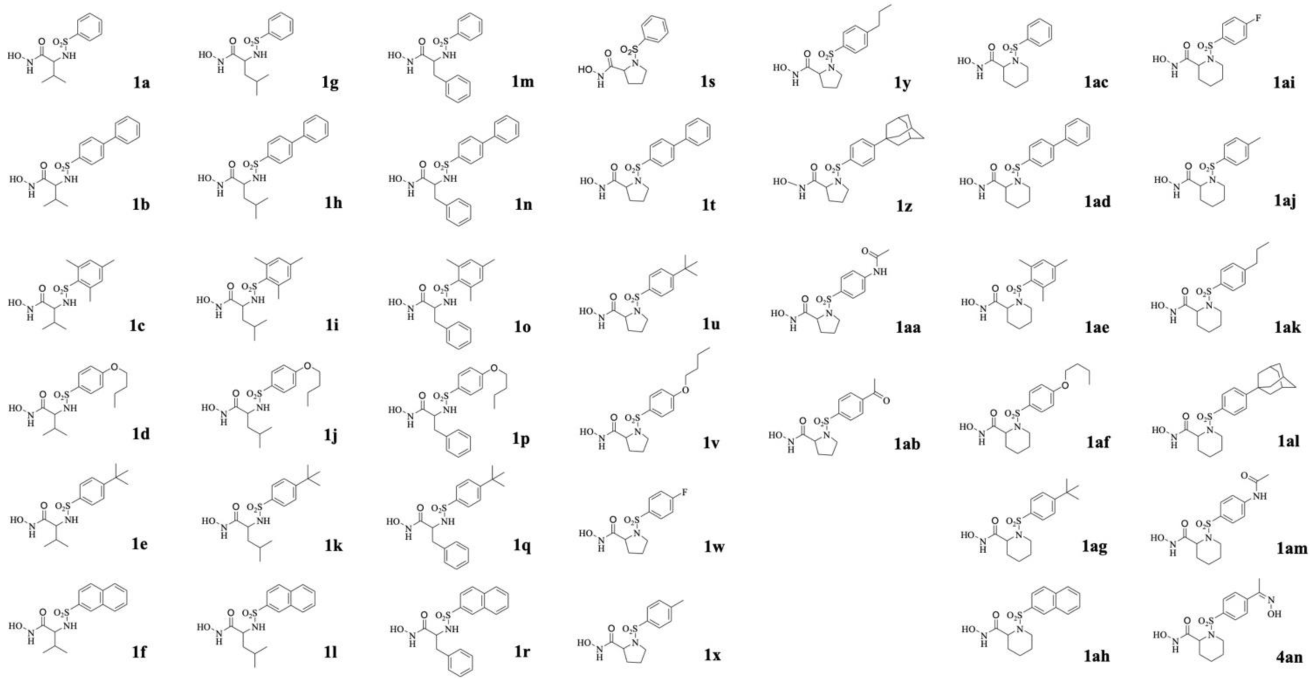

Fig. S1. Synthesis pathway and structures of hydroxamate derivatives of *N*-arylsulfonyl amino acids. (**a**) Synthetic scheme for the preparation of hydroxamate derivatives of *N*-arylsulfonyl amino acids (compounds **1** and **4**). The reaction involves the *N*-sulfonylation of amino acid **2** with *N*-arylsulfonyl chlorides in the presence of sodium carbonate, followed by conversion to hydroxamates **1** via activation with ethyl chloroformate and reaction with hydroxylamine hydrochloride in the presence of *N*-methyl morpholine (NMO) and triethylamine (TEA). Compounds **3an** illustrate typical yields, with oxime **4an** resulting from an unexpected modification in the acetyl group. (**b**) Structures of the 40 synthesized hydroxamate derivatives (**1a**-**1an** and **4an**), showing variations in the *N*-arylsulfonyl and amino acid groups. These modifications aim to optimize selectivity for MMP-2 over MMP-1 by adjusting steric bulk and hydrophobicity to enhance binding within the MMP-2 active site.

I. Characterization data

***N*-hydroxy-3-methyl-2-(phenylsulfonamido)butanamide (1a):** ^{1}H NMR (500 MHz, DMSO-*d6*) δ 10.51 (s, 1H), 8.78 (s, 1H), 7.92 (d, 1H, *J*=6.0Hz), 7.76 (d, 2H, *J*=8.0Hz), 7.58 (m, 1H), 7.52 (d, 2H, *J*=8.0Hz), 4.02 (m, 1H), 1.76 (m, 1H), 0.74 (d, 3H, *J*=6.5Hz), 0.72 (d, 3H, *J*=6.5Hz); ^{13}C NMR (100 MHz, DMSO-*d6*) δ 167.1, 142.0, 132.6, 129.3 (2C), 127.0, 126.8, 60.4, 31.2, 19.5, 18.3; HRMS *m/z* calculated for $C_{11}H_{16}N_2O_4S$ $[M-H]^-$ 271.07581, found 271.07489.

2-([1,1'-biphenyl]-4-sulfonamido)-*N*-hydroxy-3-methylbutanamide (1b): ^{1}H NMR (500 MHz, DMSO-*d6*) δ 10.56 (s, 1H), 8.79 (s, 1H), 8.00 (s, 1H), 7.83 (m, 4H), 7.73 (d, 2H, *J*=7.0Hz), 7.51 (m, 2H), 7.43 (m, 1H), 3.33 (m, 1H), 1.77 (m, 1H), 0.77 (d, 3H, *J*=6.5Hz), 0.75 (d, 3H, *J*=5.5Hz); ^{13}C NMR (100 MHz, DMSO-*d6*) δ 167.12, 143.94, 140.89, 139.03, 129.57 (2C), 128.88, 127.63 (4C), 127.48, 126.76, 60.19, 31.28, 19.39, 19.05; HRMS *m/z* calculated for $C_{17}H_{20}N_2O_4S$ $[M-H]^-$ 347.10711, found 347.11441.

***N*-hydroxy-3-methyl-2-((2,4,6-trimethylphenyl)sulfonamido)butanamide (1c):** ^{1}H NMR (500 MHz, DMSO-*d6*) δ 10.43 (s, 1H), 8.79 (s, 1H), 7.60 (d, 1H, *J*=9.5Hz), 6.98 (s, 2H), 3.16 (m, 1H), 2.55 (s, 3H), 2.50 (s, 3H), 2.24 (s, 3H), 1.76 (m, 1H), 0.70 (m, 6H); ^{13}C NMR (100 MHz, DMSO-*d6*) δ 167.52, 140.99, 138.66, 137.59, 132.08, 132.06, 131.81, 59.64, 31.42, 23.08, 22.96, 20.84, 19.63; HRMS *m/z* calculated for $C_{14}H_{22}N_2O_4S$ $[M-H]^-$ 313.12276, found 313.12277.

2-((4-butoxyphenyl)sulfonamido)-*N*-hydroxy-3-methylbutanamide (1d): ^{1}H NMR (500 MHz, DMSO-*d6*) δ 10.47 (s, 1H), 8.77 (s, 1H), 7.70 (m, 1H), 7.66 (m, 2H), 7.02 (d, 2H, *J*=9.0Hz), 4.03 (t, 2H, *J*=6.5Hz), 3.24 (m, 1H), 1.70 (m, 3H), 1.45 (m, 2H), 0.93 (m, 3H), 0.72 (m, 6H); ^{13}C NMR (100 MHz, DMSO-*d6*) δ 161.52, 136.47, 128.11 (4C), 114.86, 68.07 (2C), 31.00 (2C), 19.12, 14.11 (3C); HRMS *m/z* calculated for $C_{15}H_{24}N_2O_5S$ $[M-H]^-$ 343.13332, found 343.13321.

2-((4-(*tert*-butyl)phenyl)sulfonamido)-*N*-hydroxy-3-methylbutanamide (1e): ^{1}H NMR (500 MHz, DMSO-*d6*) δ 10.50 (s, 1H), 8.80 (s, 1H), 7.81 (m, 1H), 7.68 (d, 2H, *J*=8.0Hz), 7.54 (d, 2H, *J*=8.0Hz), 3.27 (m, 1H), 1.75 (m, 1H), 1.29 (s, 9H), 0.73 (d, 3H, *J*=6.5Hz), 0.70 (d, 3H, *J*=6.5Hz) ; ^{13}C NMR (100 MHz, DMSO-*d6*) δ 167.26, 155.48, 139.31, 126.69 (2C), 126.10, 125.98, 60.13, 35.24, 31.30, 31.22 (2C), 19.34, 18.95 (2C); HRMS *m/z* calculated for $C_{15}H_{24}N_2O_4S$ $[M-H]^-$ 327.13841, found 327.13840.

***N*-hydroxy-3-methyl-2-(naphthalene-2-sulfonamido)butanamide (1f):** ^{1}H NMR (500 MHz, DMSO-*d6*) δ 10.51 (s, 1H), 8.76 (s, 1H), 8.37 (s, 1H), 8.07 (m, 4H), 7.80 (d, 1H, *J*=8.5Hz), 7.67 (m, 2H), 1.77 (m, 1H), 1.16 (m, 1H), 0.72 (m, 6H); ^{13}C NMR (100 MHz, DMSO-*d6*) δ 167.13, 139.11, 134.52, 132.08, 129.61, 129.51, 129.44, 128.96, 127.86, 126.16, 122.9, 60.23, 31.30, 19.38, 19.01; HRMS *m/z* calculated for $C_{15}H_{18}N_2O_4S$ $[M-H]^-$ 321.09146, found 321.09146.

***N*-hydroxy-4-methyl-2-(phenylsulfonamido)pentanamide (1g):** ^{1}H NMR (500 MHz, DMSO-*d6*) δ 10.65 (s, 1H), 8.81 (s, 1H), 8.00 (m, 1H), 7.77 (m, 2H), 7.57 (m, 1H), 7.55 (m, 2H), 3.48 (m, 1H), 1.43 (m, 1H), 1.35-1.20 (m, 2H), 0.72 (d, 3H, *J*=6.5Hz), 0.60 (d, 3H, *J*=7.0Hz) ; ^{13}C NMR (100 MHz, DMSO-*d6*) δ 169.59, 143.93, 130.67, 128.99, 128.79, 119.11 (2C), 60.43, 49.53, 31.15, 24.65, 24.62; HRMS *m/z* calculated for $C_{12}H_{18}N_2O_4S$ $[M-H]^-$ 285.09146, found 285.09146.

2-([1,1'-biphenyl]-4-sulfonamido)-*N*-hydroxy-4-methylpentanamide (1h): ^{1}H NMR (500 MHz, DMSO-*d6*) δ 10.66 (s, 1H), 8.81 (s, 1H), 8.03 (d, 1H, *J*=6.0Hz), 7.83 (m, 4H), 7.73 (d, 2H, *J*=7.0Hz), 7.51

(m, 2H), 7.43 (m, 1H), 3.59 (q, 1H), 1.41 (m, 1H), 1.33-1.21 (m, 2H), 0.75 (d, 3H, *J*=6.5Hz), 0.64 (d, 3H, *J*=7.0Hz) ; ^{13}C NMR (100 MHz, DMSO-*d6*) δ 167.87, 144.10, 143.42, 140.67, 139.19, 129.59, 129.57, 128.90, 128.83, 127.63, 127.55, 127.50, 126.76, 52.78, 42.16, 24.26, 22.89, 22.19; HRMS *m/z* calculated for $C_{18}H_{22}N_2O_4S$ [M-H]$^-$ 361.12276, found 361.12274.

***N*-hydroxy-4-methyl-2-((2,4,6-trimethylphenyl)sulfonamido)pentanamide (1i):** ^{1}H NMR (500 MHz, DMSO-*d6*) δ 10.51 (s, 1H), 8.88 (s, 1H), 7.77 (m, 1H), 6.98 (s, 2H), 3.45 (m, 1H), 2.55 (s, 3H), 2.50 (s, 3H), 2.24 (s, 3H), 1.41 (m, 1H), 1.33-1.20 (m, 2H), 0.73 (d, 3H, *J*=6.5Hz), 0.55 (d, 3H, *J*=6.5Hz) ; ^{13}C NMR (100 MHz, DMSO-*d6*) δ 168.41, 140.98, 138.82, 135.52 (2C), 131.89, 131.80, 52.14, 42.34, 24.20, 23.80, 23.09, 23.03, 20.85, 20.79; HRMS *m/z* calculated for $C_{15}H_{24}N_2O_4S$ [M-H]$^-$ 327.13841, found 327.13840.

2-((4-butoxyphenyl)sulfonamido)-*N*-hydroxy-4-methylpentanamide (1j): ^{1}H NMR (500 MHz, DMSO-*d6*) δ 10.61 (s, 1H), 8.77 (s, 1H), 7.76 (m, 1H), 7.67 (d, 2H, *J*=9.0Hz), 7.04 (d, 2H, *J*=8.5Hz), 4.04 (t, 2H, *J*=6.5Hz), 3.50 (m, 1H), 1.70 (m, 2H), 1.45 (m, 3H), 1.27 (m, 1H), 1.19 (m, 1H), 0.93 (t, 3H, *J*=7.0Hz), 0.77-0.72 (d, 3H, *J*=6.5Hz), 0.63 (d, 3H, *J*=6.0Hz) ; ^{13}C NMR (100 MHz, DMSO-*d6*) δ 170.54, 161.89, 161.67, 129.21, 129.02, 114.87, 114.52, 68.10, 52.69, 42.13, 30.98, 24.22, 22.87, 20.62, 19.12, 14.12; HRMS *m/z* calculated for $C_{16}H_{26}N_2O_5S$ [M-H]$^-$ 357.14897, found 357.14899.

2-((4-(*tert*-butyl)phenyl)sulfonamido)-*N*-hydroxy-4-methylpentanamide (1k): ^{1}H NMR (500 MHz, DMSO-*d6*) δ 10.64 (s, 1H), 8.83 (s, 1H), 7.88 (m, 1H), 7.70 (d, 2H, *J*=9.0Hz), 7.57 (d, 2H, *J*=8.5Hz), 3.49 (m, 1H), 1.32-1.24 (m, 11H), 1.12 (m, 1H), 0.69 (d, 3H, *J*=6.5Hz), 0.55 (d, 3H, *J*=6.5Hz); ^{13}C NMR (100 MHz, DMSO-*d6*) δ 188.22, 155.18, 141.82, 126.17 (2C), 125.98 (2C), 58.22, 37.68, 31.32 (3C), 24.11, 23.93, 22.95, 22.78; HRMS *m/z* calculated for $C_{16}H_{26}N_2O_4S$ [M-H]$^-$ 341.15406, found 341.15405.

***N*-hydroxy-4-methyl-2-(naphthalene-2-sulfonamido)pentanamide (1l):** ^{1}H NMR (500 MHz, DMSO-*d6*) δ 10.64 (s, 1H), 8.76 (s, 1H), 8.38 (s, 1H), 8.07 (m, 4H), 7.80 (d, 1H, *J*=9.0Hz), 7.67 (m, 2H), 3.62 (m, 1H), 1.38 (m, 1H), 1.25 (m, 2H), 0.68 (d, 3H, *J*=6.5Hz), 0.54 (d, 3H, *J*=6.5Hz); ^{13}C NMR (100 MHz, DMSO-*d6*) δ 167.92, 138.88, 134.55, 132.08, 129.59, 129.54, 129.01, 128.28, 127.91, 127.51, 122.82, 52.81, 42.21, 24.20, 22.86, 22.08; HRMS *m/z* calculated for $C_{16}H_{20}N_2O_4S$ [M-H]$^-$ 335.10711, found 335.10715.

***N*-hydroxy-3-phenyl-2-(phenylsulfonamido)propanamide (1m):** ^{1}H NMR (500 MHz, DMSO-*d6*) δ 10.60 (s, 1H), 8.85 (s, 1H), 8.21 (m, 1H), 7.55 (m, 3H), 7.43 (m, 2H), 7.16 (m, 3H), 7.06 (m, 2H), 3.78 (m, 1H), 2.71 (m, 1H), 2.57 (m, 1H); ^{13}C NMR (100 MHz, DMSO-*d6*) δ 167.21, 144.61, 141.80, 137.34, 132.49, 132.25, 129.59, 129.40, 129.09, 128.53, 126.87, 126.58, 126.04, 55.93; HRMS *m/z* calculated for $C_{15}H_{16}N_2O_4S$ [M-H]$^-$ 319.07581, found 319.07727.

2-([1,1'-biphenyl]-4-sulfonamido)-*N*-hydroxy-3-phenylpropanamide (1n): ^{1}H NMR (500 MHz, DMSO-*d6*) δ 10.07 (s, 1H), 8.88 (s, 1H), 8.27 (d, 2H, *J*=9.0Hz), 7.69 (m, 3H), 7.59 (m, 2H), 7.53 (m, 2H), 7.43 (m, 1H), 7.16 (m, 3H), 7.07 (m, 2H), 3.82 (m, 1H), 2.80 (m, 1H), 2.63 (m, 1H); ^{13}C NMR (100 MHz, DMSO-*d6*) δ 163.11, 140.55, 139.18, 137.38, 129.63, 129.58, 128.84 (3C), 128.50 (2C), 127.65 (2C), 127.48 (2C), 127.22 (3C), 126.75, 54.03; HRMS *m/z* calculated for $C_{21}H_{20}N_2O_4S$ [M-H]$^-$ 395.10711, found 395.10715.

***N*-hydroxy-3-phenyl-2-((2,4,6-trimethylphenyl)sulfonamido)propanamide (1o):** ^{1}H NMR (500 MHz,

DMSO-*d6*) δ 10.59 (s, 1H), 8.87 (s, 1H), 7.89 (d, 1H, *J*=9.5Hz), 7.07 (m, 3H), 6.95 (m, 2H), 6.82 (m, 2H), 3.67 (m, 1H), 2.76 (m, 1H), 2.60 (m, 1H), 2.49 (s, 6H), 2.26 (s, 3H) ; ^{13}C NMR (100 MHz, DMSO-*d6*) δ 160.11, 147.05, 140.99, 138.70, 138.66, 137.69, 131.81, 129.59, 129.37, 129.10, 128.84, 128.75, 127.66, 54.11, 23.03, 22.85, 20.84, 20.79; HRMS *m/z* calculated for $C_{18}H_{22}N_2O_4S$ $[M-H]^-$ 361.12276, found 361.13016.

2-((4-butoxyphenyl)sulfonamido)-*N*-hydroxy-3-phenylpropanamide (1p): ^{1}H NMR (500 MHz, DMSO-*d6*) δ 10.58 (s, 1H), 8.83 (s, 1H), 8.00 (d, 1H, *J*=9.0Hz), 7.47 (m, 1H), 7.15 (m, 4H), 7.04 (m, 2H), 6.88 (m, 2H), 4.03 (t, 2H, *J*=6.5Hz), 3.73 (m, 1H), 2.77 (m, 1H), 2.53 (m, 1H), 1.72 (m, 2H), 1.44 (m, 2H), 0.92 (m, 3H); ^{13}C NMR (100 MHz, DMSO-*d6*) δ 161.9, 147.05, 136.49, 136.17, 132.62, 131.13, 130.43, 129.84, 129.71, 127.98, 114.98, 114.87, 114.14, 68.09, 40.62, 38.53, 19.12, 14.26, 14.12; HRMS *m/z* calculated for $C_{19}H_{24}N_2O_5S$ $[M-H]^-$ 391.13332, found 391.13300.

2-((4-(*tert*-butyl)phenyl)sulfonamido)-*N*-hydroxy-3-phenylpropanamide (1q): ^{1}H NMR (500 MHz, DMSO-*d6*) δ 10.61 (s, 1H), 8.88 (s, 1H), 8.13 (d, 1H, *J*=8.5Hz), 7.40 (m, 4H), 7.12 (m, 3H), 7.00 (m, 2H), 3.71 (m, 1H), 2.76 (m, 1H), 2.55 (m, 1H), 1.26 (s, 9H); ^{13}C NMR (100 MHz, DMSO-*d6*) δ 155.18, 141.82, 130.18, 129.92, 129.69, 129.59, 128.93, 128.36, 128.14, 127.66, 126.80, 126.57, 125.98, 58.22, 35.23, 31.32, 31.28, 31.19, 14.14; HRMS *m/z* calculated for $C_{19}H_{24}N_2O_4S$ $[M-H]^-$ 375.13841, found 375.13803.

***N*-hydroxy-2-(naphthalene-2-sulfonamido)-3-phenylpropanamide (1r):** ^{1}H NMR (500 MHz, DMSO-*d6*) δ 10.63 (s, 1H), 8.84 (s, 1H), 8.32 (d, 1H, *J*=9.5Hz), 8.19 (s, 1H), 8.19 (m, 2H), 7.92 (d, 1H, *J*=9.5Hz), 7.65 (m, 2H), 7.56 (m, 1H), 7.28-7.00 (m, 5H), 3.84 (q, 1H), 2.78 (m, 1H), 2.59 (m, 1H); ^{13}C NMR (100 MHz, DMSO-*d6*) δ 141.66, 138.84, 137.76, 134.54, 134.35, 132.17, 130.36, 129.87, 129.59, 129.02, 128.86, 128.26, 127.99, 127.84, 126.17, 122.95, 122.57, 58.50, 14.19; HRMS *m/z* calculated for $C_{19}H_{18}N_2O_4S$ $[M-H]^-$ 369.09146, found 369.09277.

***N*-hydroxy-1-(phenylsulfonyl)pyrrolidine-2-carboxamide (1s):** ^{1}H NMR (500 MHz, DMSO-*d6*) δ 10.68 (s, 1H), 8.95 (s, 1H), 7.85 (m, 2H), 7.70 (m, H), 7.62 (m, 2H), 3.90 (m, 1H), 3.39 (m, 1H), 3.13 (m, 1H), 1.81 (m, 1H) 1.73 (m, 1H) 1.64 (m, 1H), 1.43 (m, 1H); ^{13}C NMR (100 MHz, DMSO-*d6*) δ 168.33, 158.34, 137.29, 133.63, 129.89, 127.74, 127.48, 60.43, 49.52, 31.18, 24.66; HRMS *m/z* calculated for $C_{11}H_{14}N_2O_4S$ $[M-H]^-$ 269.06016, found 269.06018.

1-([1,1'-biphenyl]-4-ylsulfonyl)-*N*-hydroxypyrrolidine-2-carboxamide (1t): ^{1}H NMR (500 MHz, DMSO-*d6*) δ 10.72 (s, 1H), 8.98 (s, 1H), 7.93 (m, 4H), 7.78 (m, 2H), 7.53 (m,2H), 7.45 (m, H), 3.97 (m, 1H), 3.44 (m, 1H), 3.20 (m, 1H), 1.86 (m, 1H) 1.73 (m, 2H) 1.50 (m, 1H); ^{13}C NMR (100 MHz, DMSO-*d6*) δ 168.37, 144.94, 138.77, 136.17, 129.64 (2C), 129.11, 128.49 (2C), 127.98 (2C), 127.59 (2C), 59.85, 49.54, 31.25, 24.73; HRMS *m/z* calculated for $C_{17}H_{18}N_2O_4S$ $[M-H]^-$ 345.09146, found 345.09143.

1-((4-(*tert*-butyl)phenyl)sulfonyl)-*N*-hydroxypyrrolidine-2-carboxamide (1u): ^{1}H NMR (500 MHz, DMSO-*d6*) δ 10.66 (s, 1H), 8.94 (s, 1H), 7.78 (m, 2H), 7.64 (m, 2H), 3.91 (m, 1H), 3.30 (m, 1H), 3.13 (m, 1H), 1.88 (m, 1H) 1.75 (m, 1H) 1.66 (m, 1H), 1.46 (m, 1H), 1.31 (s, 9H) ; ^{13}C NMR (100 MHz, DMSO-*d6*) δ 168.41, 156.55, 134.61, 127.72 (2C), 126.66 (2C), 59.81, 49.48, 35.37 (2C), 31.26 (3C), 24.69; HRMS *m/z* calculated for $C_{15}H_{22}N_2O_4S$ $[M-H]^-$ 325.12276, found 325.12277.

1-((4-butoxyphenyl)sulfonyl)-*N*-hydroxypyrrolidine-2-carboxamide (1v): ^{1}H NMR (500 MHz, DMSO-*d6*) δ 10.65 (s, 1H), 8.93 (s, 1H), 7.76 (m, 2H), 7.12 (m, 2H), 4.07 (t, 2H, *J*=6.5Hz), 3.88 (m, 1H),

3.39 (m, 1H), 3.11 (m, 1H), 1.83 (m, 1H), 1.74 (m, 3H), 1.63 (m, 1H), 1.45 (m, 3H), 0.94 (t, 3H, *J*=8.5Hz) ; ^{13}C NMR (100 MHz, DMSO-*d6*) δ 168.44, 162.65, 130.00, 129.71, 128.73, 115.35, 68.23, 59.87, 49.48, 31.15, 31.01, 24.67, 19.13, 14.12; HRMS *m/z* calculated for $C_{15}H_{22}N_2O_5S$ $[M-H]^-$ 341.11767, found 341.11765.

1-((4-fluorophenyl)sulfonyl)-*N*-hydroxypyrrolidine-2-carboxamide (1w): ^{1}H NMR (500 MHz, DMSO-*d6*) δ 10.69 (s, 1H), 8.96 (s, 1H), 7.93 (m, 2H), 7.38 (m, 2H), 3.90 (m, 1H), 3.39 (m, 1H), 3.16 (m, 1H), 1.85 (m, 1H), 1.73 (m, 2H), 1.49 (m, 1H); ^{13}C NMR (100 MHz, DMSO-*d6*) δ 173.62, 168.24, 166.34, 163.84, 133.85, 130.79, 116.93, 61.06, 49.48, 31.20, 24.69; HRMS *m/z* calculated for $C_{11}H_{13}FN_2O_4S$ $[M-H]^-$ 287.05074, found 287.05075.

***N*-hydroxy-1-tosylpyrrolidine-2-carboxamide (1x):** ^{1}H NMR (500 MHz, DMSO-*d6*) δ 10.66 (s, 1H), 8.94 (s, 1H), 7.73 (m, 2H), 7.45 (m, 2H), 3.89 (m, 1H), 3.38 (m, 1H), 3.13 (m, 1H), 2.41 (s, 3H), 1.82 (m, 1H), 1.73 (m, 1H), 1.63 (m, 1H), 1.44 (m, 1H); ^{13}C NMR (100 MHz, DMSO-*d6*) δ 168.39, 144.00, 134.41, 130.33 (2C), 127.72, 127.56, 59.85, 49.50, 31.16, 24.65, 21.47; HRMS *m/z* calculated for $C_{12}H_{16}N_2O_4S$ $[M-H]^-$ 283.07581, found 283.07587.

***N*-hydroxy-1-((4-propylphenyl)sulfonyl)pyrrolidine-2-carboxamide (1y):** ^{1}H NMR (500 MHz, DMSO-*d6*) δ 10.66 (s, 1H), 8.94 (s, 1H), 7.76 (m, 2H), 7.46 (m, 2H), 3.90 (m, 1H), 3.38 (m, 1H), 3.14 (m, 1H), 2.66 (t, 2H, *J*=9Hz), 1.83 (m, 1H), 1.75 (m, 1H), 1.65 (m, 3H), 1.44 (m, H), 0.89 (t, 3H, *J*=7.5Hz) ; ^{13}C NMR (100 MHz, DMSO-*d6*) δ 168.39, 148.37, 134.68, 129.73 (2C), 127.84, 127.56, 59.84, 49.51, 37.36, 31.17, 24.66, 24.12, 14.00; HRMS *m/z* calculated for $C_{14}H_{20}N_2O_4S$ $[M-H]^-$ 311.10711, found 311.10715.

1-((4-((3*r*,5*r*,7*r*)-adamantan-1-yl)phenyl)sulfonyl)-*N*-hydroxypyrrolidine-2-carboxamide (1z): ^{1}H NMR (500 MHz, DMSO-*d6*) δ 10.66 (s, 1H), 8.95 (s, 1H), 7.77 (m, 2H), 7.62 (m, 2H), 3.91 (m, 1H), 3.39 (m, 1H), 3.12 (m, 1H), 2.15 (s, 3H), 1.89-1.75 (m, 13H), 1.65 (m, 1H), 1.50 (m, 1H), 1.16 (m, 1H) ; ^{13}C NMR (100 MHz, DMSO-*d6*) δ 168.41, 156.56, 134.59, 127.77 (2C), 126.27 (2C), 60.42, 59.82, 49.49, 42.63, 40.62, 40.41, 40.21, 40.00, 39.79, 39.58, 31.22, 28.64, 24.68, 15.10; HRMS *m/z* calculated for $C_{21}H_{28}N_2O_4S$ $[M-H]^-$ 403.16971, found 403.16974.

1-((4-acetamidophenyl)sulfonyl)-*N*-hydroxypyrrolidine-2-carboxamide (1aa): ^{1}H NMR (500 MHz, DMSO-*d6*) δ 10.64 (s, 1H), 10.38 (s, 1H), 8.91 (s, 1H), 7.79 (m, 4H), 3.90 (m, 1H), 3.41 (m, 1H), 3.11 (m, 1H), 2.09 (s, 3H), 1.82 (m, 1H), 1.70 (m, 2H), 1.42 (m, 1H); ^{13}C NMR (100 MHz, DMSO-*d6*) δ 169.58, 168.41, 158.34, 143.90, 130.69, 129.00 (2C), 119.11 (2C), 60.44, 59.91, 49.53, 31.15, 24.65; HRMS *m/z* calculated for $C_{13}H_{17}N_3O_5S$ $[M-H]^-$ 326.08162, found 326.08163.

1-((4-acetylphenyl)sulfonyl)-*N*-hydroxypyrrolidine-2-carboxamide (1ab): ^{1}H NMR (500 MHz, DMSO-*d6*) δ 10.72 (s, 1H), 8.97 (s, 1H), 8.15 (m, 2H), 7.97 (m, 2H), 3.95 (m, 1H), 3.43 (m, 1H), 3.13 (m, 1H), 2.65 (s, 3H), 1.86 (m, 1H), 1.72 (m, 2H), 1.48 (m, 1H); ^{13}C NMR (100 MHz, DMSO-*d6*) δ 197.86, 168.17, 141.05, 140.34, 129.56, 128.11, 127.83 (2C), 59.84, 49.51, 31.23, 27.53, 24.69; HRMS *m/z* calculated for $C_{13}H_{16}N_2O_5S$ $[M-H]^-$ 311.07072, found 311.07062.

***N*-hydroxy-1-(phenylsulfonyl)piperidine-2-carboxamide (1ac):** ^{1}H NMR (500 MHz, DMSO-*d6*) δ 10.62 (s, 1H), 8.79 (s, 1H), 7.78 (m, 2H), 7.66 (m, 1H), 7.57 (m, 2H), 4.34 (m, 1H), 3.63 (m, 1H), 3.47 (m, 1H), 1.78 (m, 1H), 1.55 (m, 1H) 1.45 – 1.40 (m, 3H), 1.17 (m, 1H); ^{13}C NMR (100 MHz, DMSO-*d6*)

δ 167.38, 140.43, 133.14, 129.72 (2C), 127.18 (2C), 52.66, 43.11, 28.15, 24.22, 19.37; HRMS *m/z* calculated for $C_{12}H_{16}N_2O_4S$ $[M-H]^-$ 283.07581, found 283.07581.

1-([1,1'-biphenyl]-4-ylsulfonyl)-*N*-hydroxypiperidine-2-carboxamide (1ad): ^1H NMR (500 MHz, DMSO-*d6*) δ 10.67 (s, 1H), 8.80 (s, 1H), 7.87 (m, 4H), 7.77 (m, 2H), 7.52 (m, 2H), 7.45 (m, 1H), 4.38 (m, 1H), 3.66 (m, 1H), 3.50 (m, 1H), 1.81 (m, 1H), 1.59 (m, 1H), 1.47-1.43 (m, 3H), 1.26-1.15 (m, 1H); ^{13}C NMR (100 MHz, DMSO-*d6*) δ 167.41, 144.42, 139.13, 138.87, 129.62, 129.01 (2C), 127.93 (2C), 127.80, 127.65 (2C), 127.53, 52.65, 43.15, 29.33, 24.36, 19.39; HRMS *m/z* calculated for $C_{18}H_{20}N_2O_4S$ $[M-H]^-$ 359.10711, found 359.10718.

***N*-hydroxy-1-(mesitylsulfonyl)piperidine-2-carboxamide (1ae):** ^1H NMR (500 MHz, DMSO-*d6*) δ 10.48 (s, 1H), 8.86 (s, 1H), 7.05 (s, 2H), 3.69 (t, 1H, *J*=10.5Hz), 3.32 (s, 3H), 3.43 (s, 3H) 1.79 (m, 2H), 1.65-1.50 (m, 6H), 1.30-1.23 (m, 3H); ^{13}C NMR (100 MHz, DMSO-*d6*) δ 168.32, 142.52, 139.86 (2C), 133.45, 132.25 (2C), 51.35, 42.06, 29.10, 24.67, 22.65, 20.88, 19.76, 15.10; HRMS *m/z* calculated for $C_{15}H_{22}N_2O_4S$ $[M-H]^-$ 325.12276, found 325.12277.

1-((4-butoxyphenyl)sulfonyl)-*N*-hydroxypiperidine-2-carboxamide (1af): ^1H NMR (500 MHz, DMSO-*d6*) δ 10.61 (s, 1H), 8.77 (s, 1H), 7.67 (d, 2H, *J*=8.5Hz), 7.06 (d, 2H, *J*=9Hz), 4.30 (m, 1H), 4.05 (t, 2H, *J*=7Hz), 3.59 (m, 1H), 3.44 (m, 1H) 1.77-1.69 (m, 3H), 1.54-1.42 (m, 6H), 1.18 (m, 1H), 0.94 (t, 3H, *J*=7.5Hz); ^{13}C NMR (100 MHz, DMSO-*d6*) δ 167.47, 162.23, 131.84, 129.42 (2C), 115.17 (2C), 68.16, 52.57, 42.97, 31.03, 28.08, 24.21, 19.42, 19.14, 14.14; HRMS *m/z* calculated for $C_{16}H_{24}N_2O_5S$ $[M-H]^-$ 355.13332, found 355.13327.

1-((4-(*tert*-butyl)phenyl)sulfonyl)-*N*-hydroxypiperidine-2-carboxamide (1ag): ^1H NMR (500 MHz, DMSO-*d6*) δ 10.62 (s, 1H), 8.81 (s, 1H), 7.69 (m, 2H), 7.59 (m, 2H), 4.34 (m, 1H), 3.59 (m, 1H), 3.43 (m, 1H), 1.78 (m, 1H), 1.54 (m, 1H), 1.44 (m, 3H), 1.31 (s, 9H), 1.16 (m, 1H); ^{13}C NMR (100 MHz, DMSO-*d6*) δ 167.56, 156.03, 137.75, 127.18, 126.48, 52.65, 43.04, 35.31, 31.29 (3C), 28.21, 24.28, 19.44; HRMS *m/z* calculated for $C_{16}H_{24}N_2O_4S$ $[M-H]^-$ 339.13841, found 339.13844.

***N*-hydroxy-1-(naphthalen-2-ylsulfonyl)piperidine-2-carboxamide (1ah):** ^1H NMR (500 MHz, DMSO-*d6*) δ 10.64 (s, 1H), 8.78 (s, 1H), 8.44 (s, 1H), 8.14 (m, 1H), 8.10 (m, 1H), 8.04 (m, 1H), 7.76 (m, 1H), 7.68 (m, 2H), 4.42 (m, 1H), 3.72 (m, 1H), 3.48 (m, 1H), 1.77 (m, 1H), 1.55 (m, 1H), 1.42 (m, 2H), 1.16 (m, 2H),; ^{13}C NMR (100 MHz, DMSO-*d6*) δ 167.38, 137.51, 134.71, 132.26, 129.78, 129.77, 129.25, 128.28, 128.24, 127.98, 122.92, 52.79, 43.20, 28.21, 24.33, 19.39; HRMS *m/z* calculated for $C_{16}H_{18}N_2O_4S$ $[M-H]^-$ 333.09146, found 333.09131.

1-((4-fluorophenyl)sulfonyl)-*N*-hydroxypiperidine-2-carboxamide (1ai): ^1H NMR (500 MHz, DMSO-*d6*) δ 10.63 (s, 1H), 8.76 (s, 1H), 7.82 (m, 2H), 7.42 (m, 2H), 4.32 (m, 1H), 3.62 (m, 1H), 3.46 (m, 1H), 1.80 (m, 1H), 1.77 (m, 1H), 1.51-1.39 (m, 3H), 1.19 (m, 1H); ^{13}C NMR (100 MHz, DMSO-*d6*) δ 167.25, 165.97, 163.48, 136.54, 130.32, 116.91, 116.68, 52.61, 43.13, 28.38, 24.37, 19.32; HRMS *m/z* calculated for $C_{12}H_{15}FN_2O_4S$ $[M-H]^-$ 301.06639, found 301.06628.

***N*-hydroxy-1-tosylpiperidine-2-carboxamide (1aj):** ^1H NMR (500 MHz, DMSO-*d6*) δ 10.61 (s, 1H), 8.78 (s, 1H), 7.65 (d, 2H, *J*=8.5Hz), 7.37 (d, 2H, *J*=9Hz), 4.32 (m, 1H), 3.62 (m, 1H), 3.44 (m, 1H), 2.39 (s, 3H), 1.77 (m, 1H), 1.53 (m, 1H), 1.42 (m, 3H), 1.15 (m, 1H); ^{13}C NMR (100 MHz, DMSO-*d6*) δ 167.42, 143.36, 137.63, 130.15 (2C), 127.27 (2C), 52.63, 43.05, 28.08, 24.19, 21.46, 19.40; HRMS *m/z* calculated

for $C_{13}H_{18}N_2O_4S$ $[M-H]^-$ 297.09146, found 297.09146.

***N*-hydroxy-1-((4-propylphenyl)sulfonyl)piperidine-2-carboxamide (1ak):** ^1H NMR (500 MHz, DMSO-*d6*) δ 10.61 (s, 1H), 8.79 (s, 1H), 7.65 (m, 2H), 7.38 (m, 2H), 4.33 (m, 1H), 3.61 (m, 1H), 3.45 (m, 1H), 2.43 (t, 2H, *J*=7.5Hz), 1.76 (m, 1H), 1.59-1.38 (m, 6H), 1.16 (m, 1H), 0.90 (t, 3H, *J*=7.5Hz); ^{13}C NMR (100 MHz, DMSO-*d6*) δ 167.46, 147.78, 137.91, 129.55, 129.39, 127.28, 127.15, 52.66, 43.06, 37.36, 27.04, 24.18, 24.12, 19.41, 15.09; HRMS *m/z* calculated for $C_{15}H_{22}N_2O_4S$ $[M-H]^-$ 325.12276, found 325.12268.

1-((4-((3*r*,5*r*,7*r*)-adamantan-1-yl)phenyl)sulfonyl)-*N*-hydroxypiperidine-2-carboxamide (1al): ^1H NMR (500 MHz, DMSO-*d6*) δ 10.61 (s, 1H), 8.81 (s, 1H), 7.70 (m, 2H), 7.55 (m, 2H), 4.33 (m, 1H), 3.59 (m, 1H), 3.45 (m, 1H), 2.07 (s, 3H), 1.89 (m, 7H), 1.77 (m, 7H), 1.52 (m, 1H), 1.43 (m, 2H), 1.19 (m, 1H); ^{13}C NMR (100 MHz, DMSO-*d6*) δ 167.54, 156.07, 137.74, 129.57, 127.22, 126.10 (2C), 52.65, 43.05, 42.67, 40.63, 40.42, 40.21, 40.00, 39.79, 39.58, 36.69, 36.46, 28.64, 28.16, 24.25, 19.44; HRMS *m/z* calculated for $C_{22}H_{30}N_2O_4S$ $[M-H]^-$ 417.18536, found 417.18533.

1-((4-acetamidophenyl)sulfonyl)-*N*-hydroxypiperidine-2-carboxamide (1am): ^1H NMR (500 MHz, DMSO-*d6*) δ 10.60 (s, 1H), 10.32 (s, 1H), 8.78 (s, 1H), 7.74 (m, 2H), 7.68 (m, 2H), 4.30 (m, 1H), 3.61 (m, 1H), 3.45 (m, 1H), 2.09 (s, 3H), 1.76 (m, 1H), 1.51 (m, 1H), 1.39 (m, 3H), 1.16 (m, 1H); ^{13}C NMR (100 MHz, DMSO-*d6*) δ 169.49, 167.43, 143.44, 133.88, 128.43, 128.27, 119.01 (2C), 52.62, 43.02, 28.05, 24.62, 24.18, 19.39; HRMS *m/z* calculated for $C_{14}H_{19}N_3O_5S$ $[M-H]^-$ 340.09727, found 340.09729.

***N*-hydroxy-1-((4-(1-(hydroxyimino)ethyl)phenyl)sulfonyl)piperidine-2-carboxamide (4an):** ^1H NMR (500 MHz, DMSO-*d6*) δ 11.55 (s, 1H), 10.63 (s, 1H), 8.79 (s, 1H), 7.82 (m, 2H), 7.75 (m, 2H), 4.35 (m, 1H), 3.63 (m, 1H), 3.47 (m, 1H), 3.32 (s, 3H), 1.74 (m, 1H), 1.54 (m, 1H), 1.45 (m, 3H), 1.20 (m, 1H); ^{13}C NMR (100 MHz, DMSO-*d6*) δ 167.34, 152.49, 141.10, 140.04, 127.36 (2C), 126.60 (2C), 52.66, 43.13, 28.26, 24.30, 19.36, 11.84; HRMS *m/z* calculated for $C_{14}H_{19}N_3O_5S$ $[M-H]^-$ 340.09727, found 340.09729.

1H-, 13C-NMR spectra and mass spectra of the target compounds

N-hydroxy-3-methyl-2-(phenylsulfonamido)butanamide (1a)

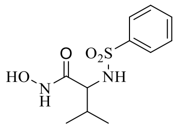

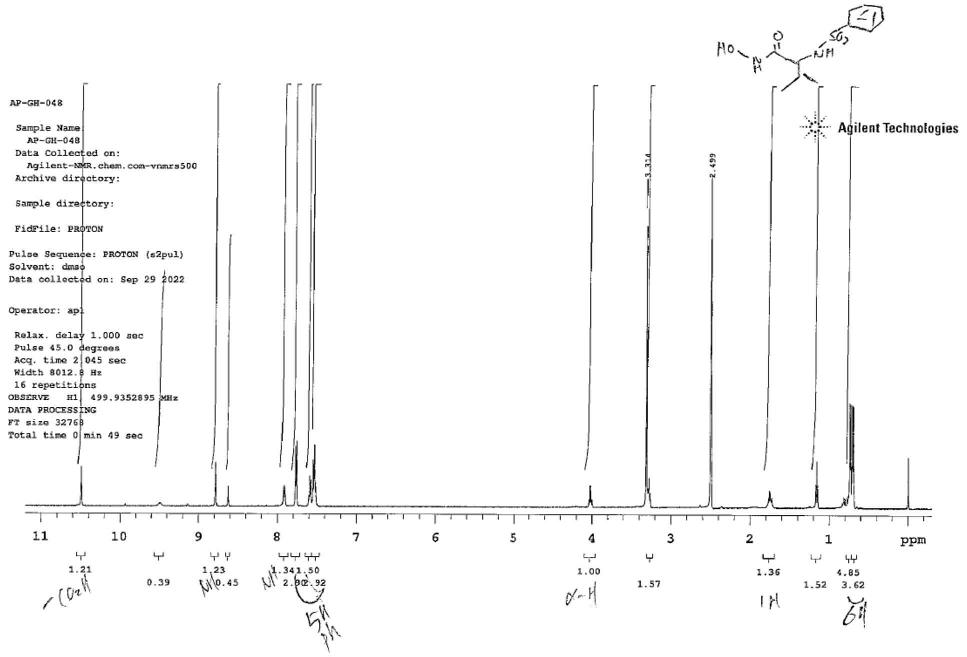

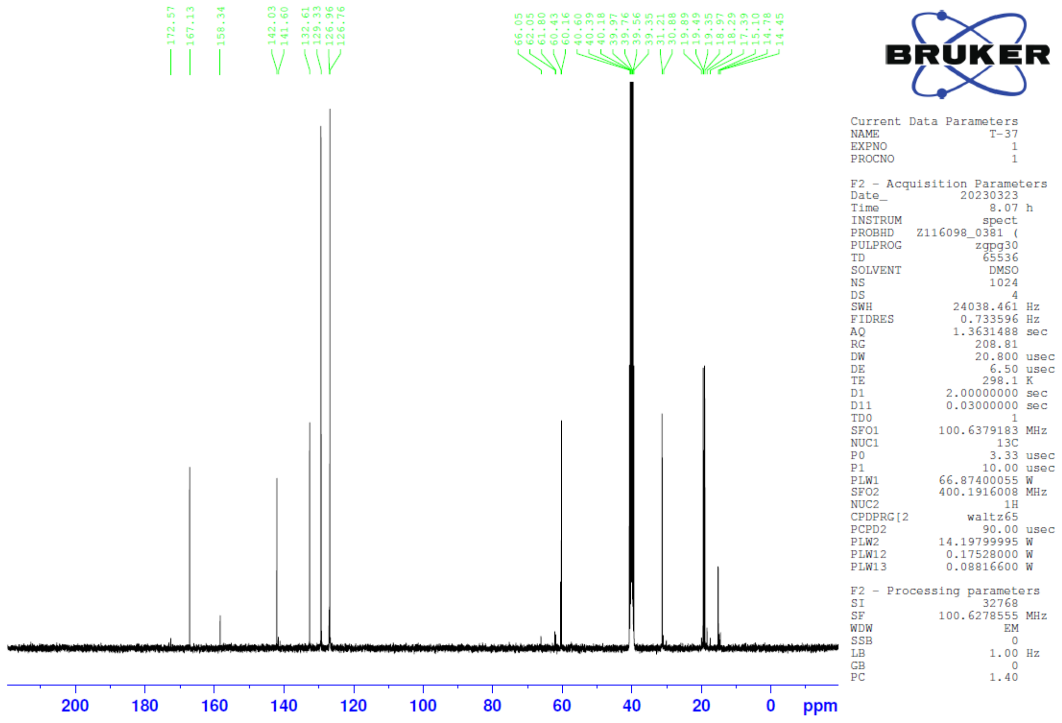

2-([1,1'-biphenyl]-4-sulfonamido)-*N*-hydroxy-3-methylbutanamide (1b)

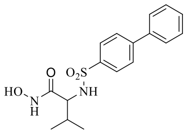

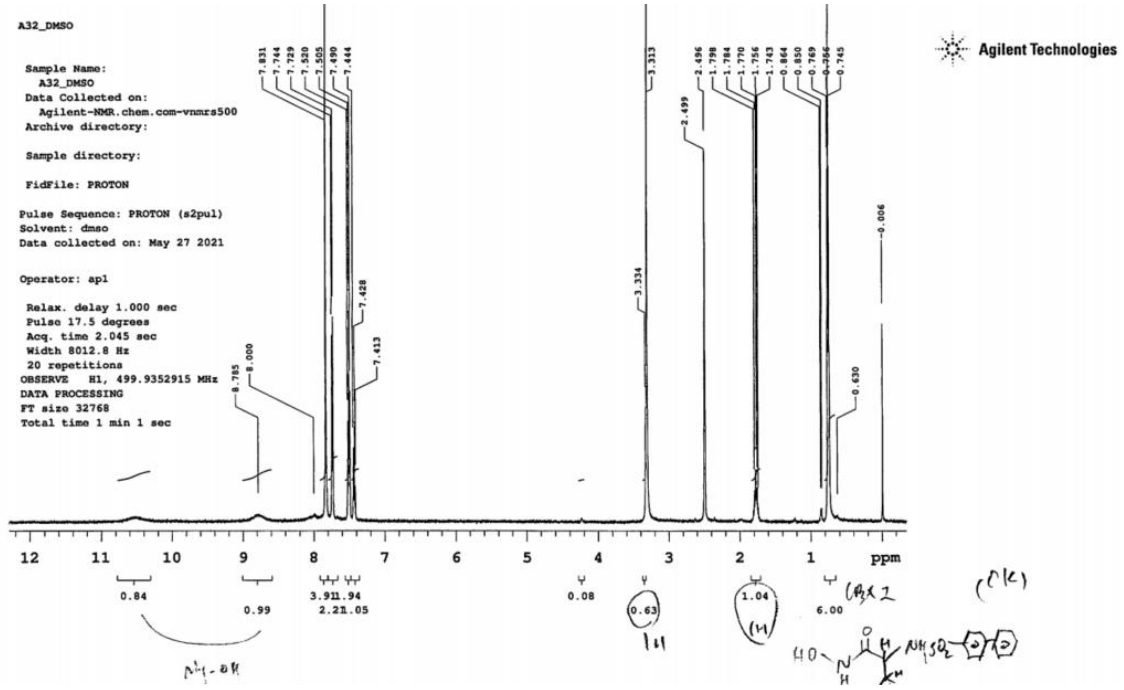

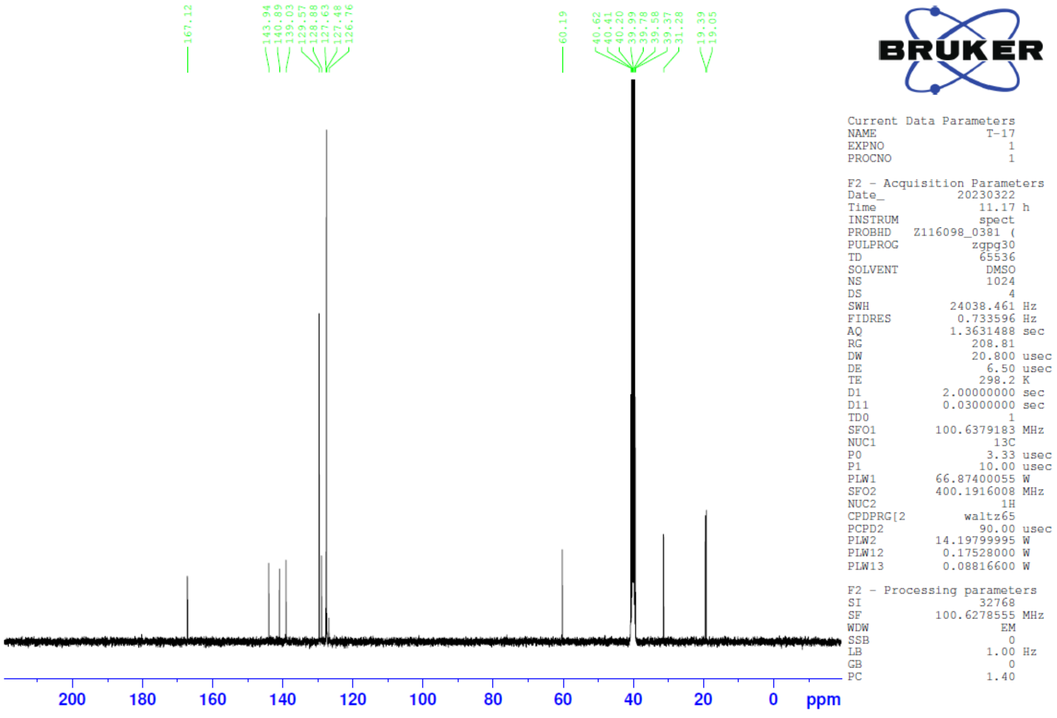

N-hydroxy-3-methyl-2-((2,4,6-trimethylphenyl)sulfonamido)butanamide (1c)

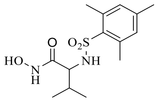

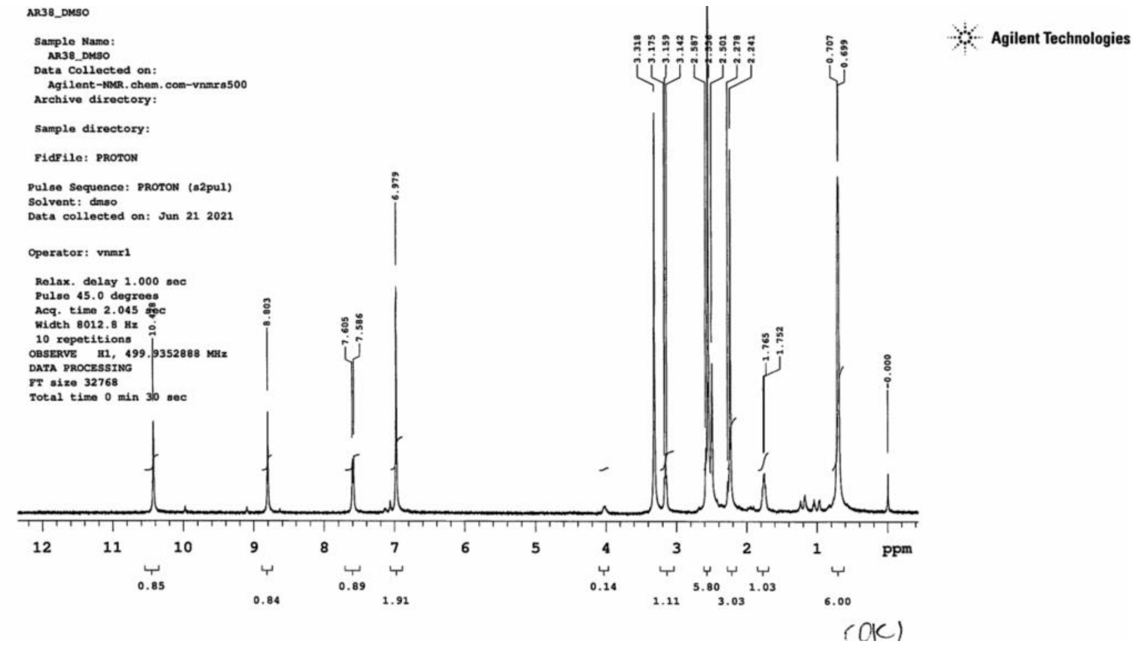

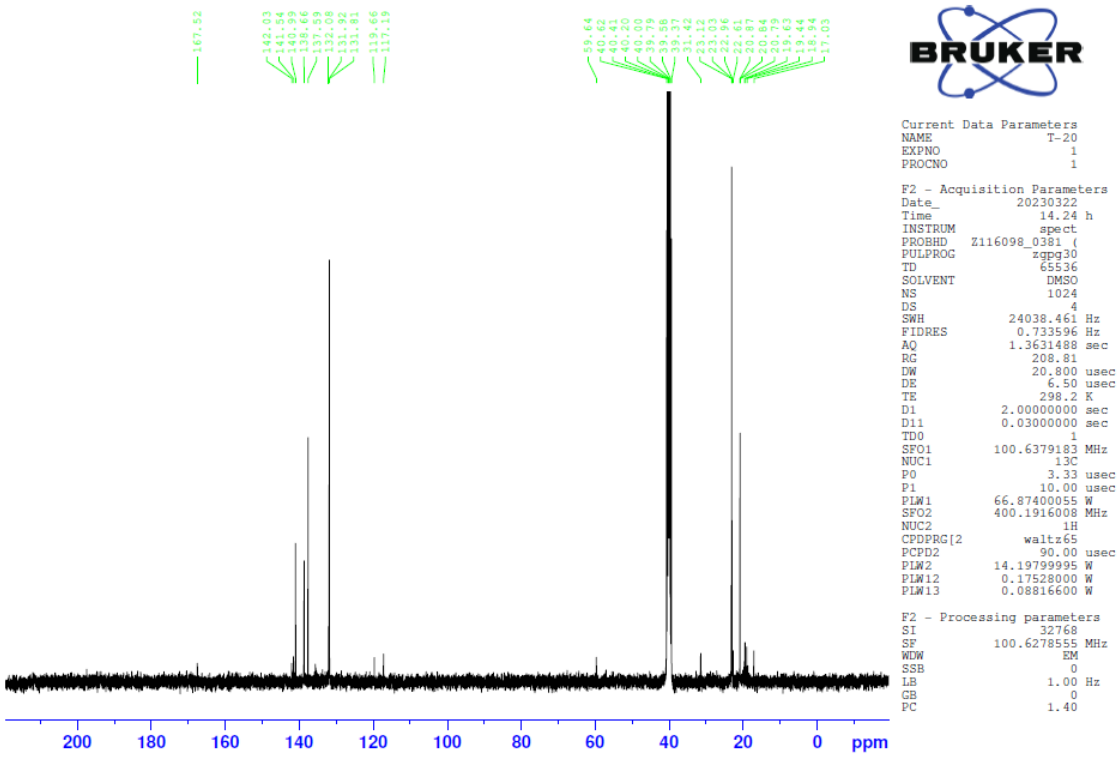

2-((4-butoxyphenyl)sulfonamido)-*N*-hydroxy-3-methylbutanamide (1d)

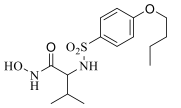

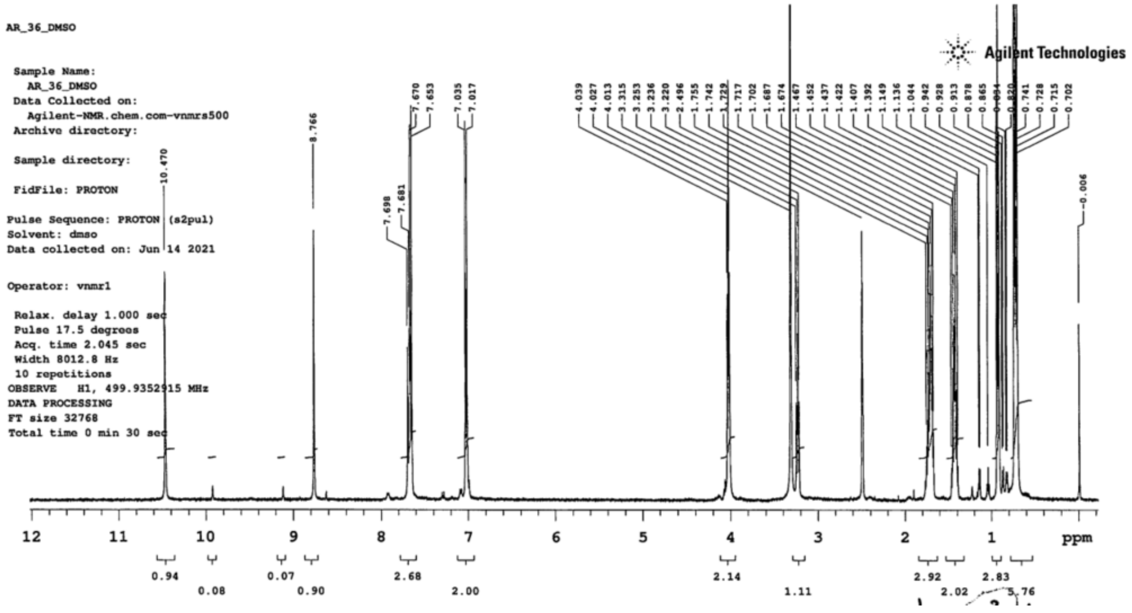

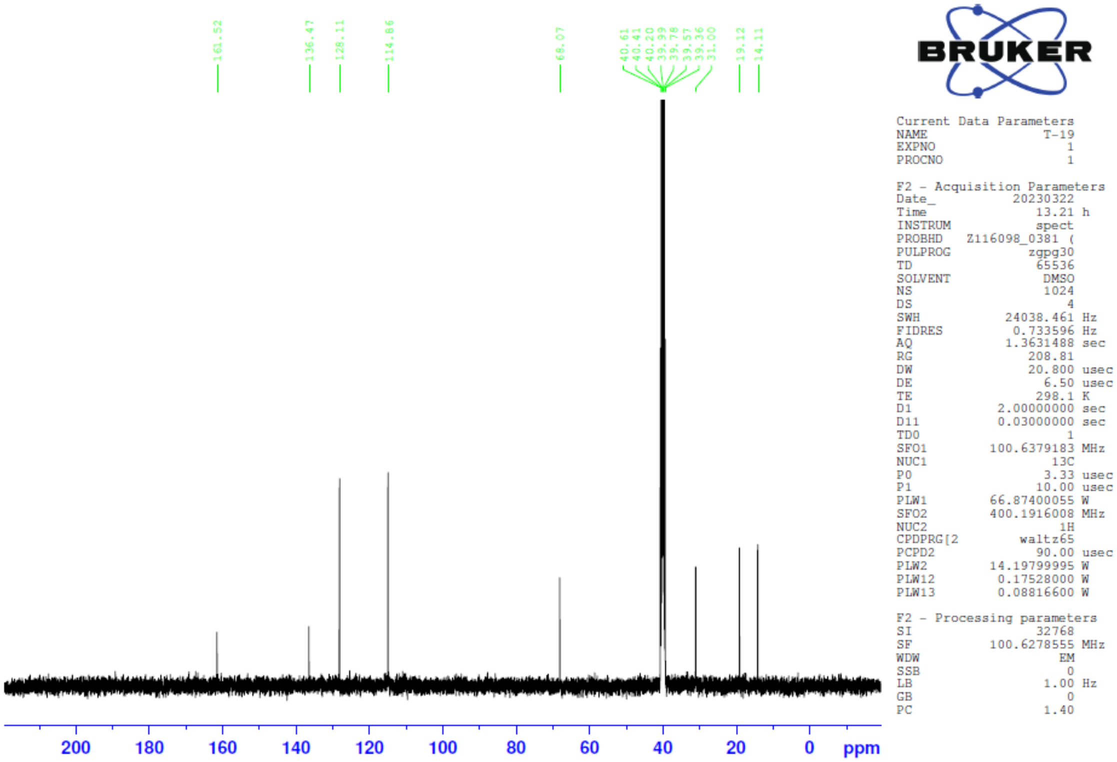

2-((4-(*tert*-butyl)phenyl)sulfonamido)-*N*-hydroxy-3-methylbutanamide (1e)

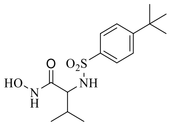

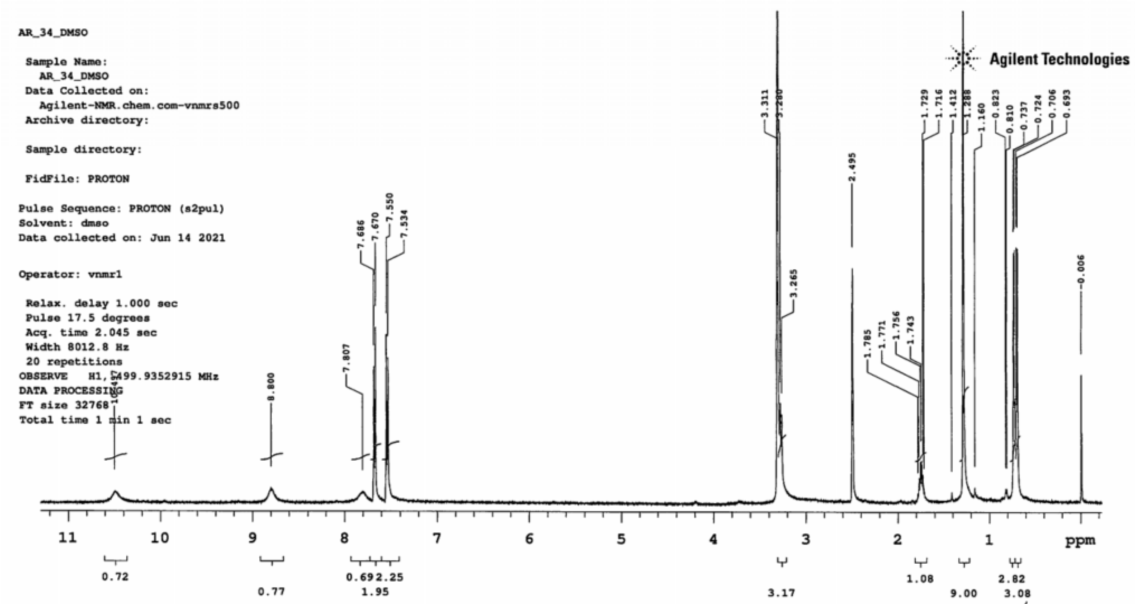

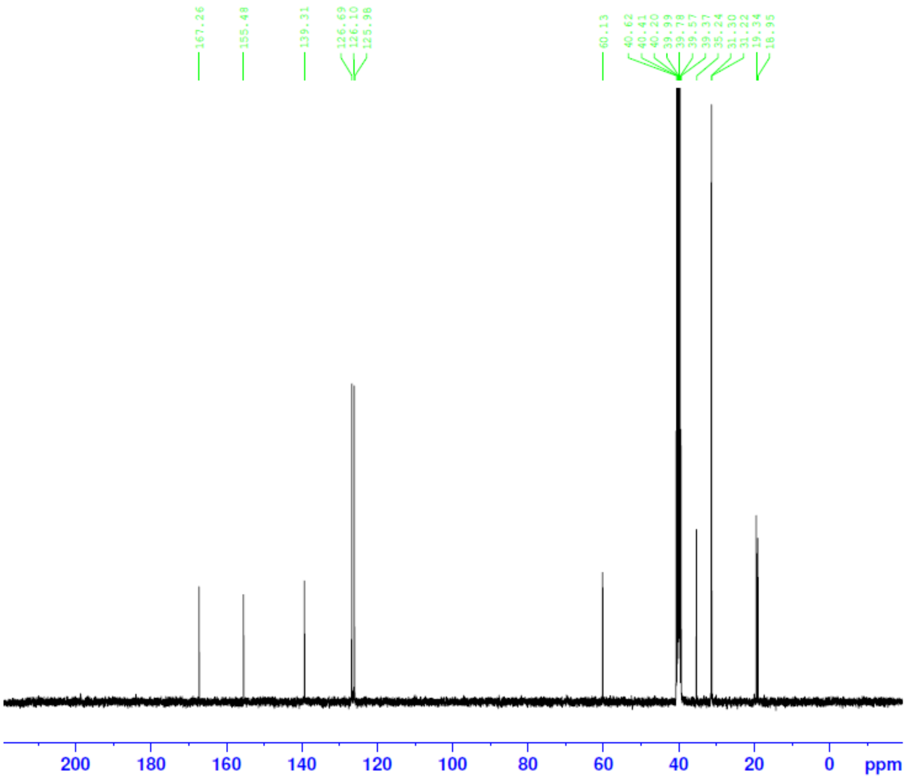

N-hydroxy-3-methyl-2-(naphthalene-2-sulfonamido)butanamide (1f)

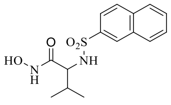

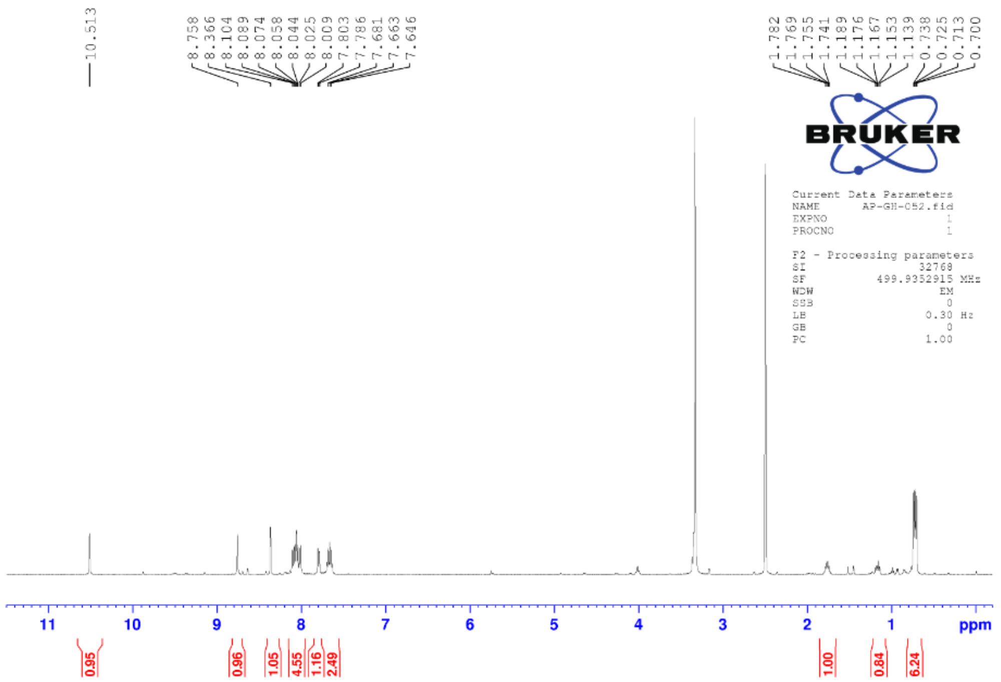

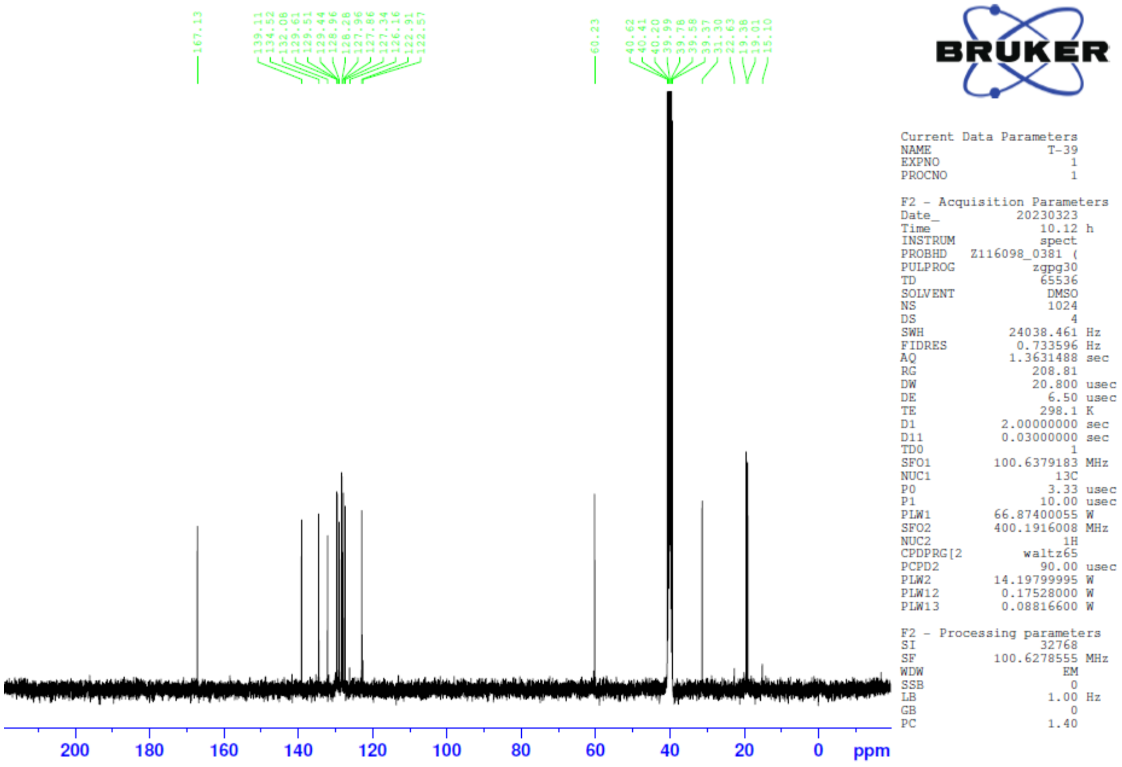

N-hydroxy-4-methyl-2-(phenylsulfonamido)pentanamide (1g)

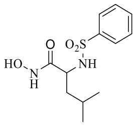

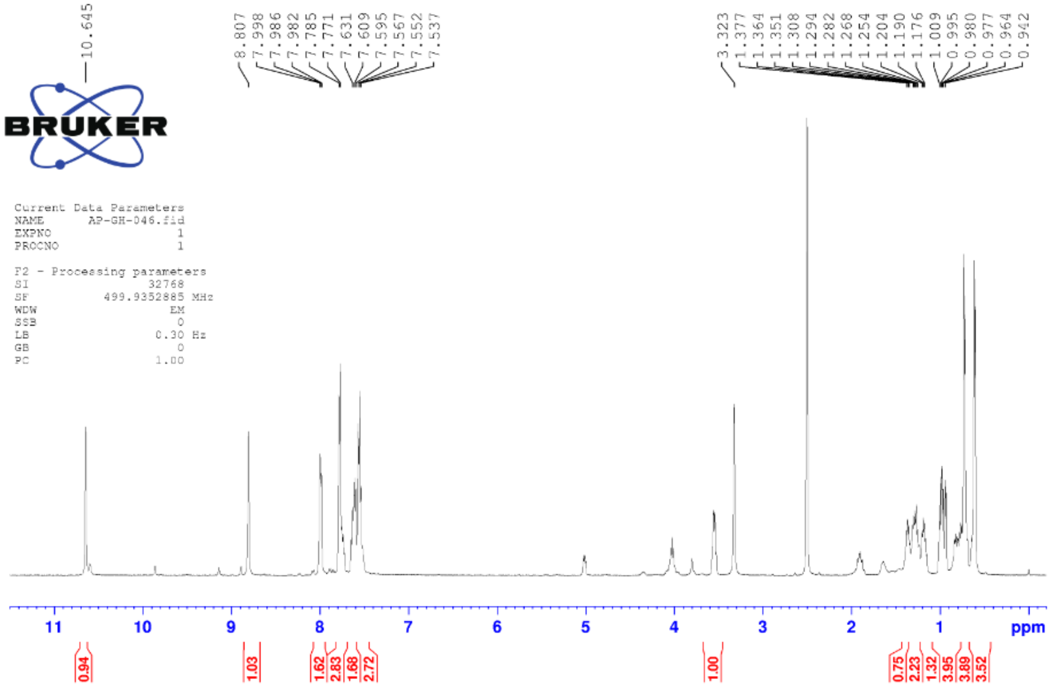

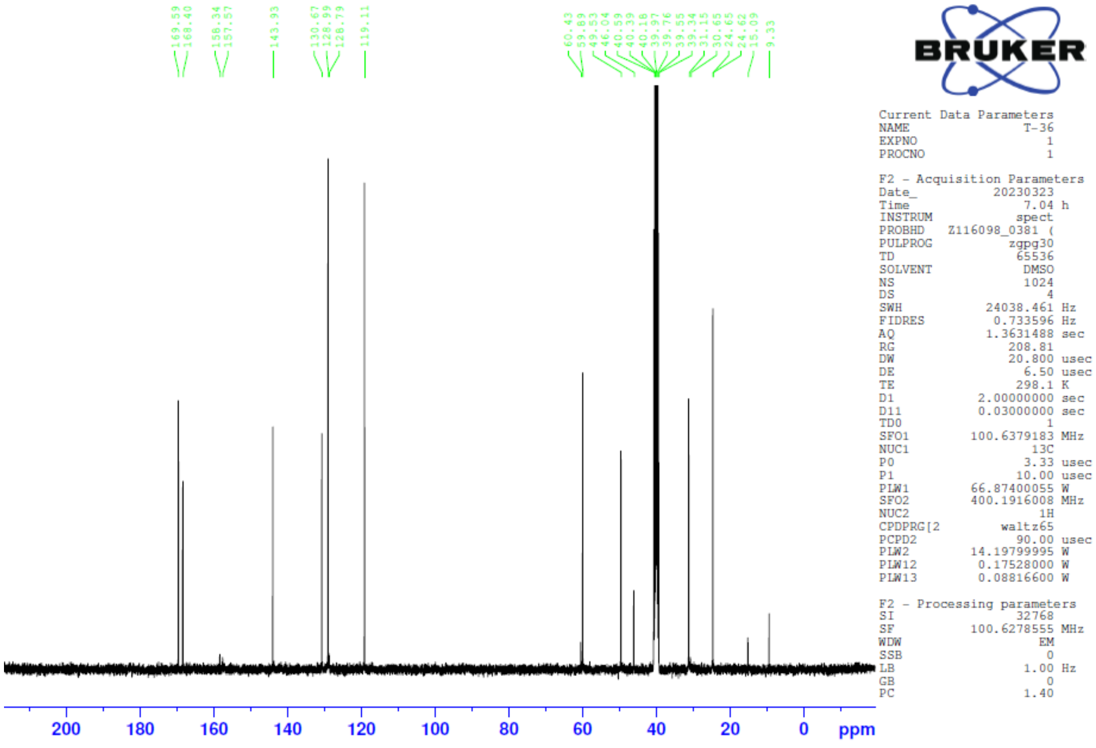

2-([1,1'-biphenyl]-4-sulfonamido)-*N*-hydroxy-4-methylpentanamide (1h)

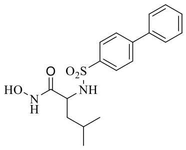

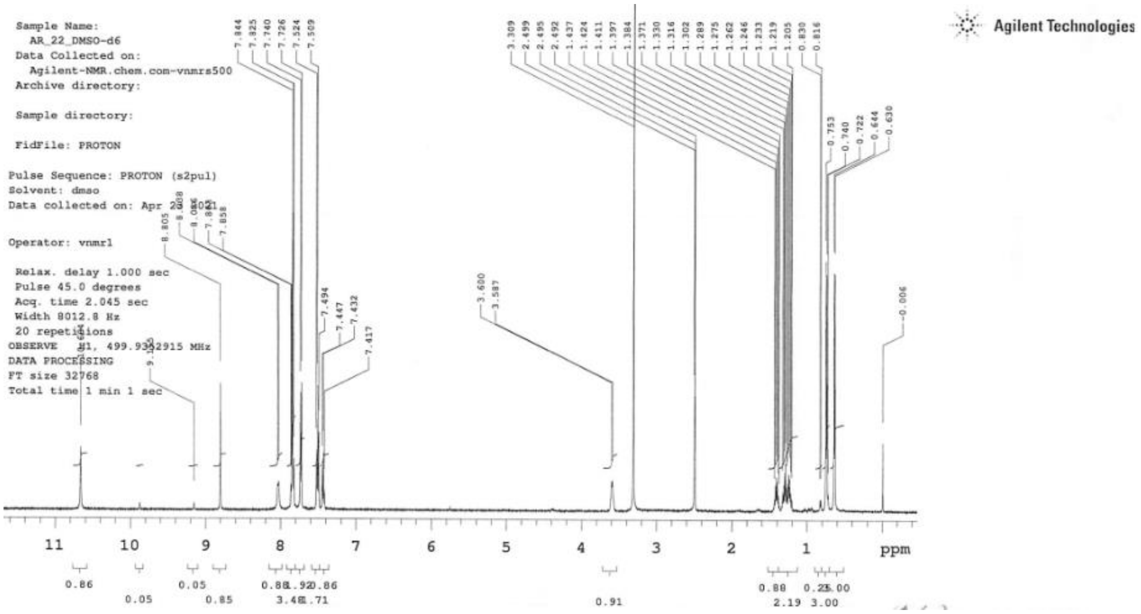

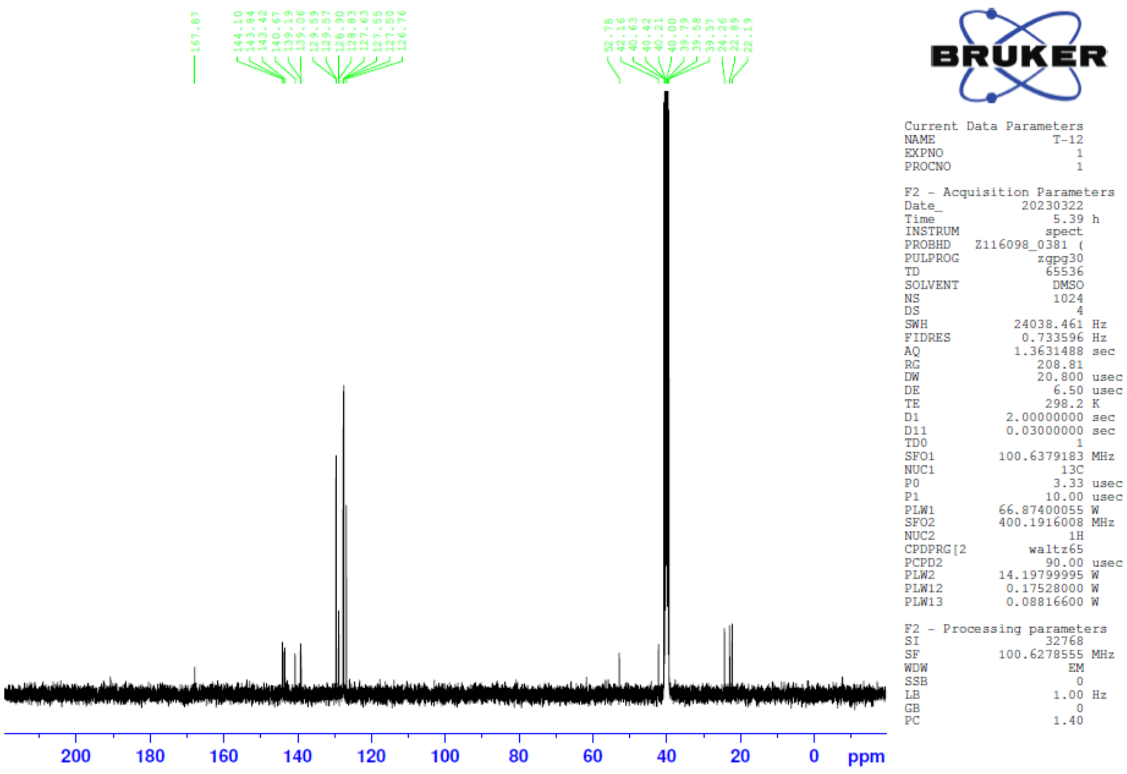

N-hydroxy-4-methyl-2-((2,4,6-trimethylphenyl)sulfonamido)pentanamide (1i)

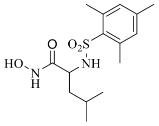

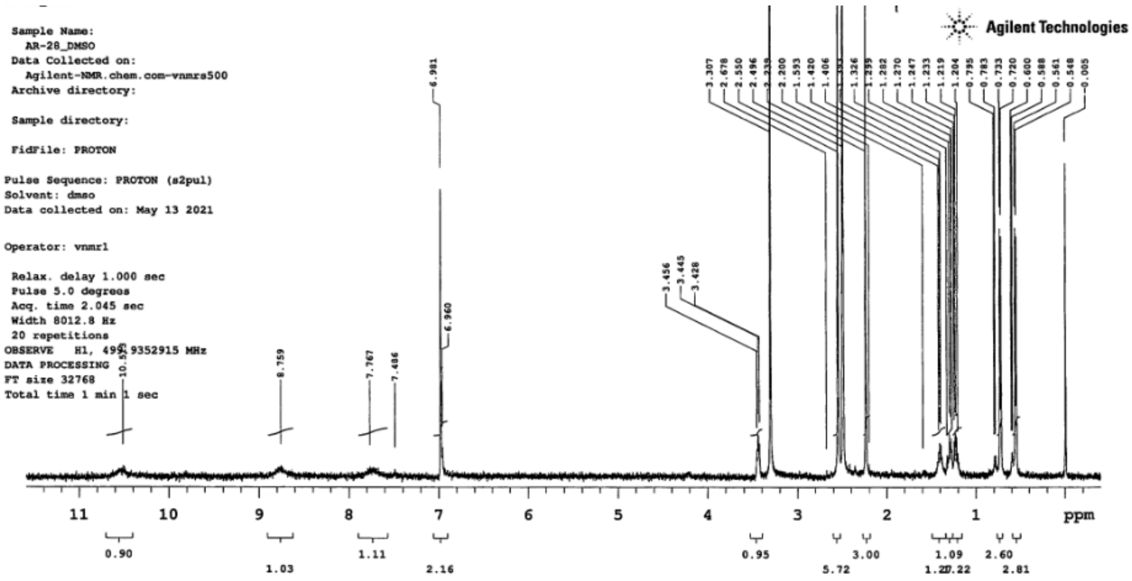

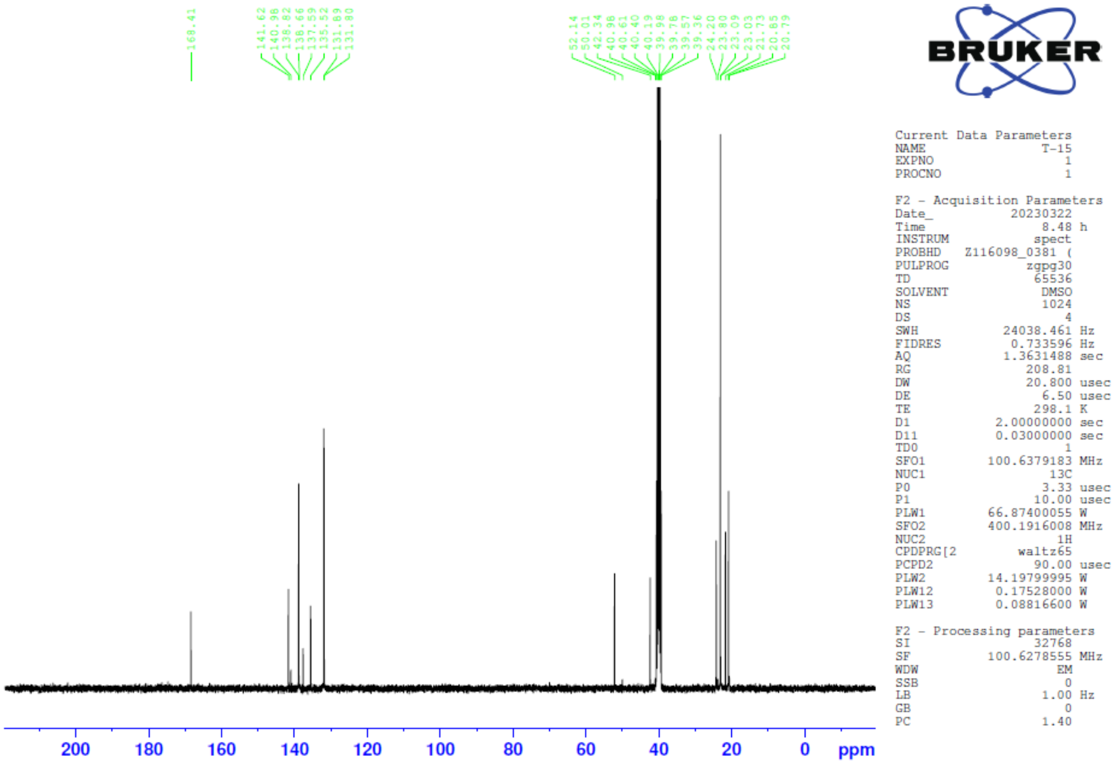

2-((4-butoxyphenyl)sulfonamido)-*N*-hydroxy-4-methylpentanamide (1j)

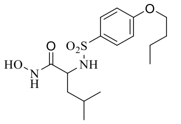

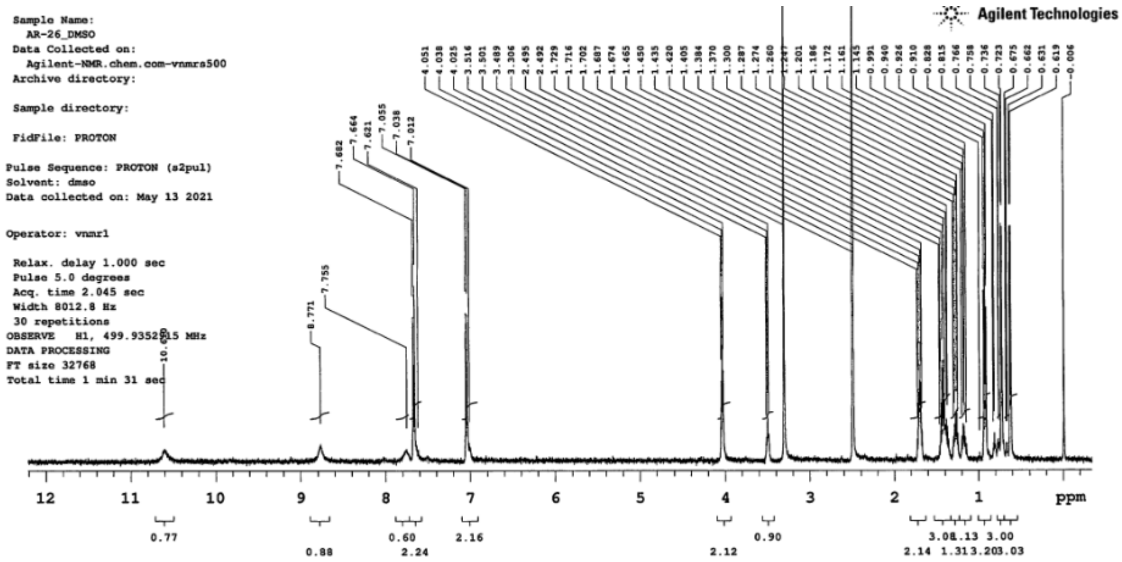

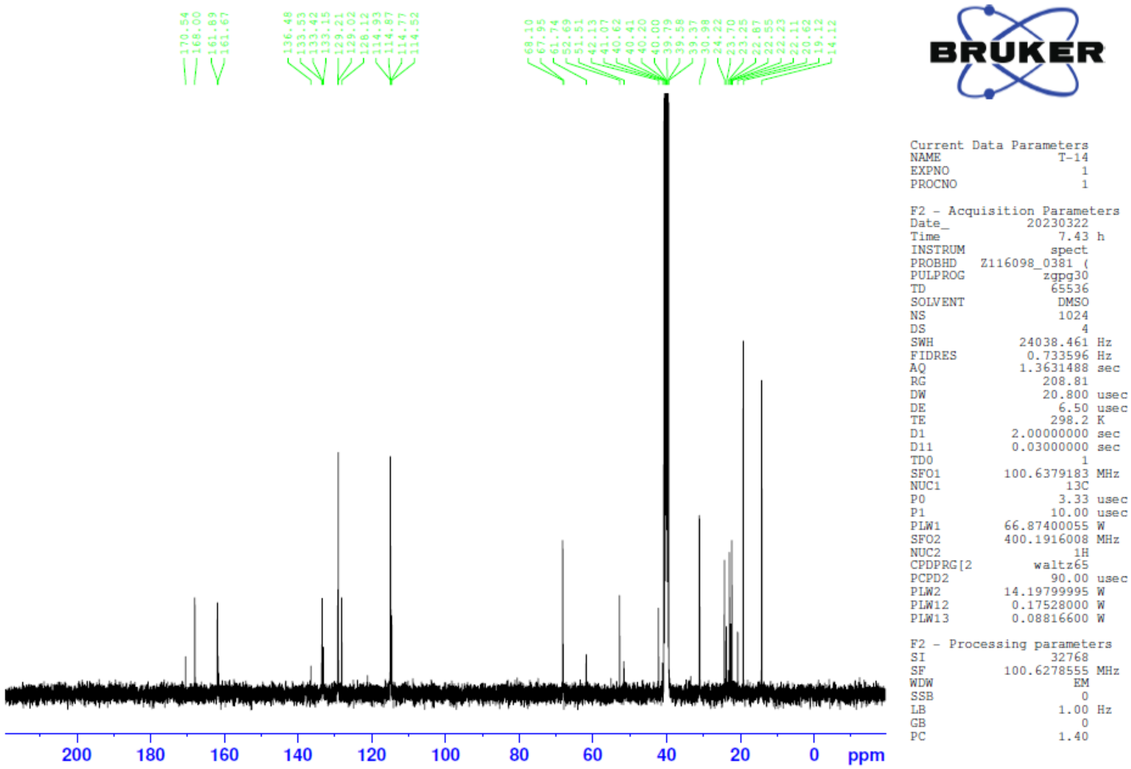

2-((4-(*tert*-butyl)phenyl)sulfonamido)-*N*-hydroxy-4-methylpentanamide (1k)

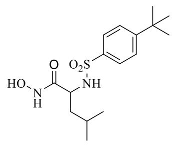

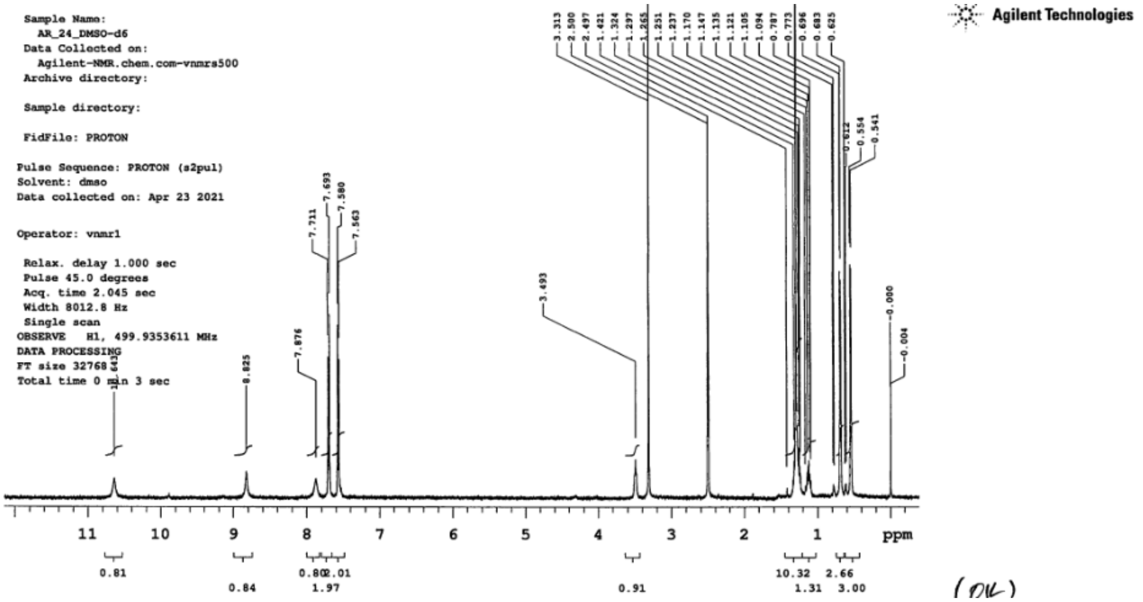

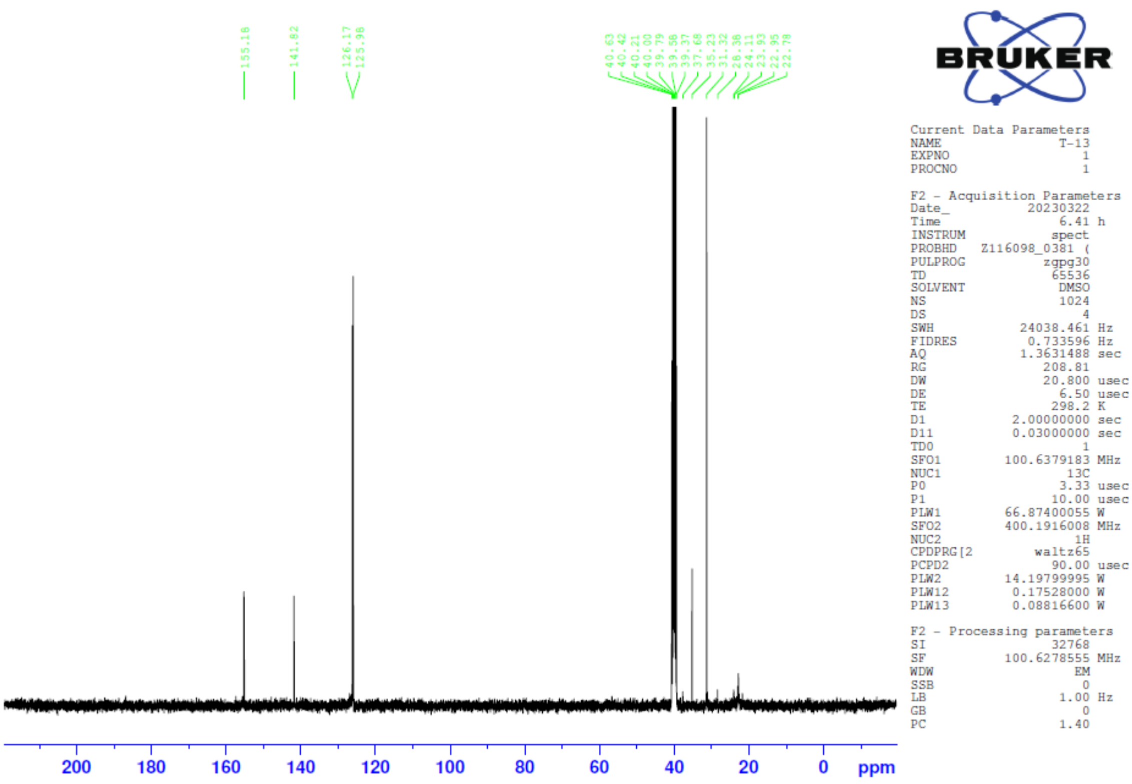

N-hydroxy-4-methyl-2-(naphthalene-2-sulfonamido)pentanamide (1l)

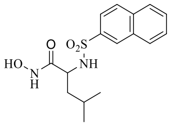

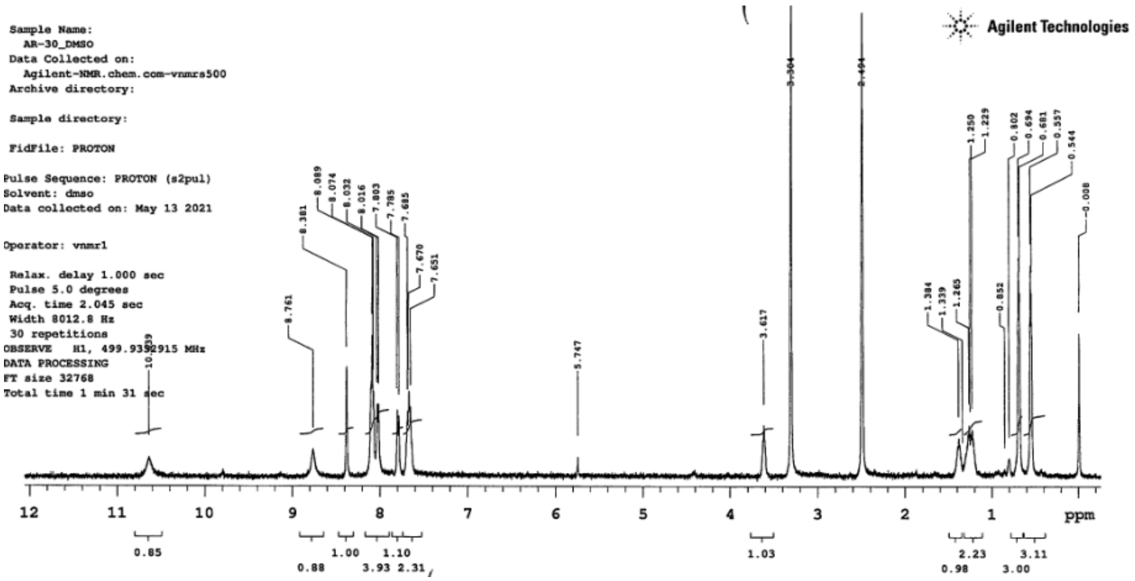

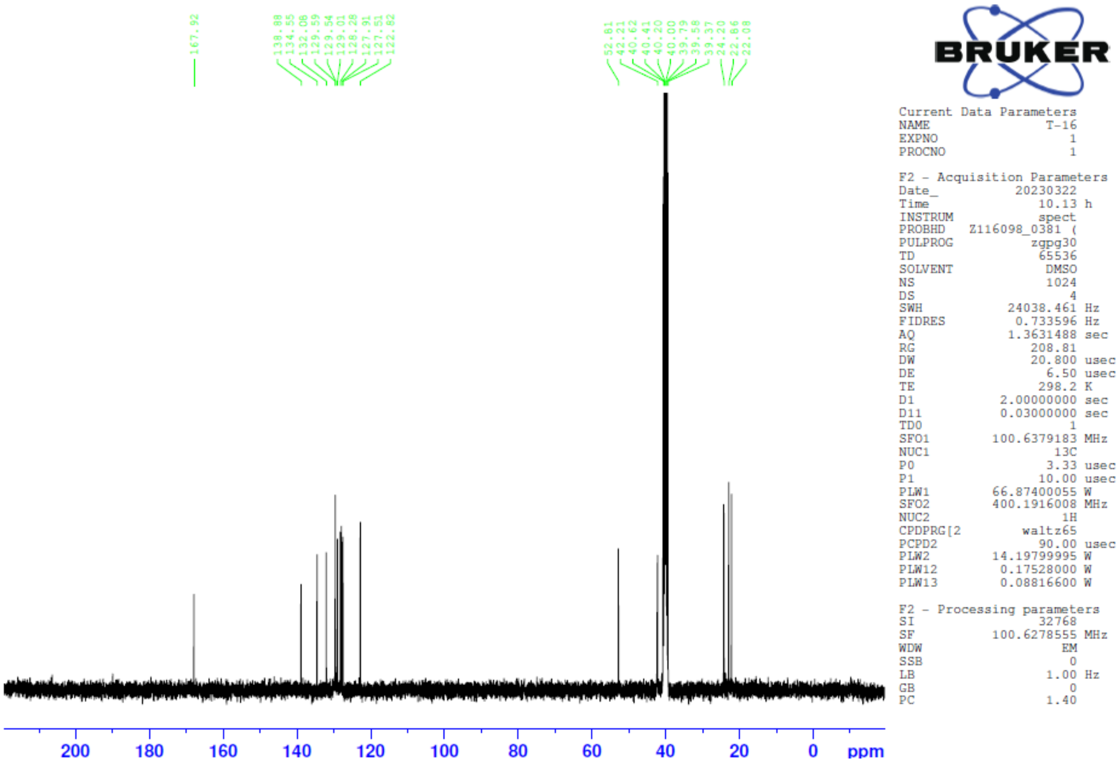

N-hydroxy-3-phenyl-2-(phenylsulfonamido)propanamide (1m)

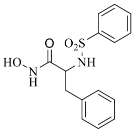

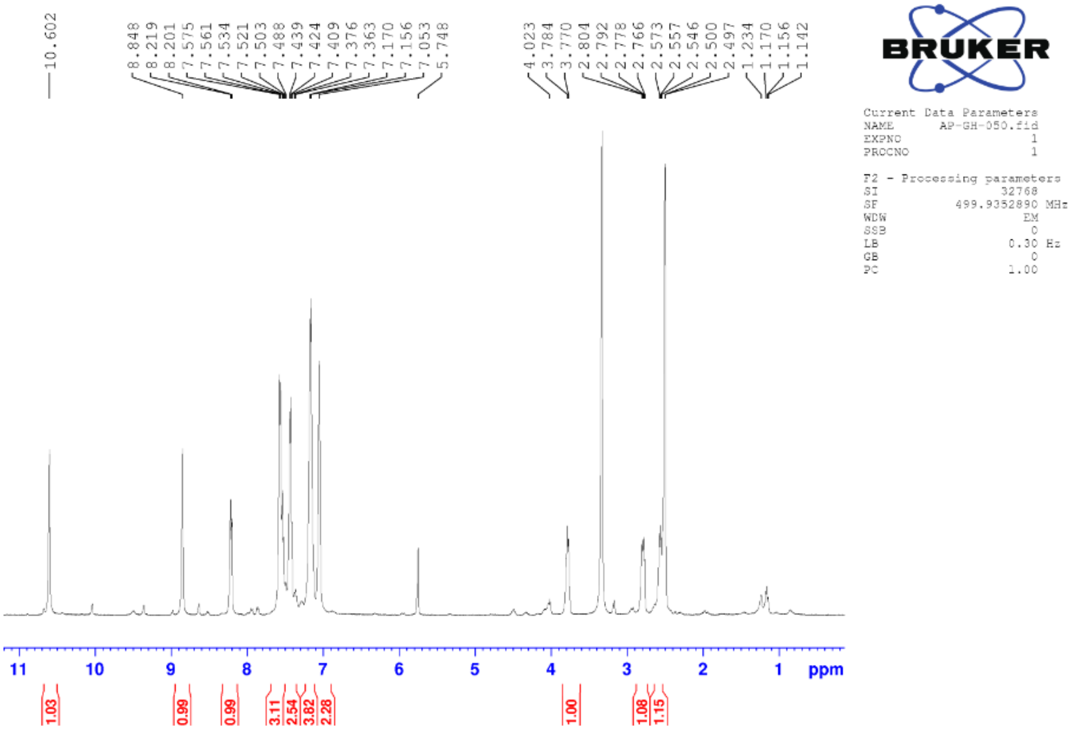

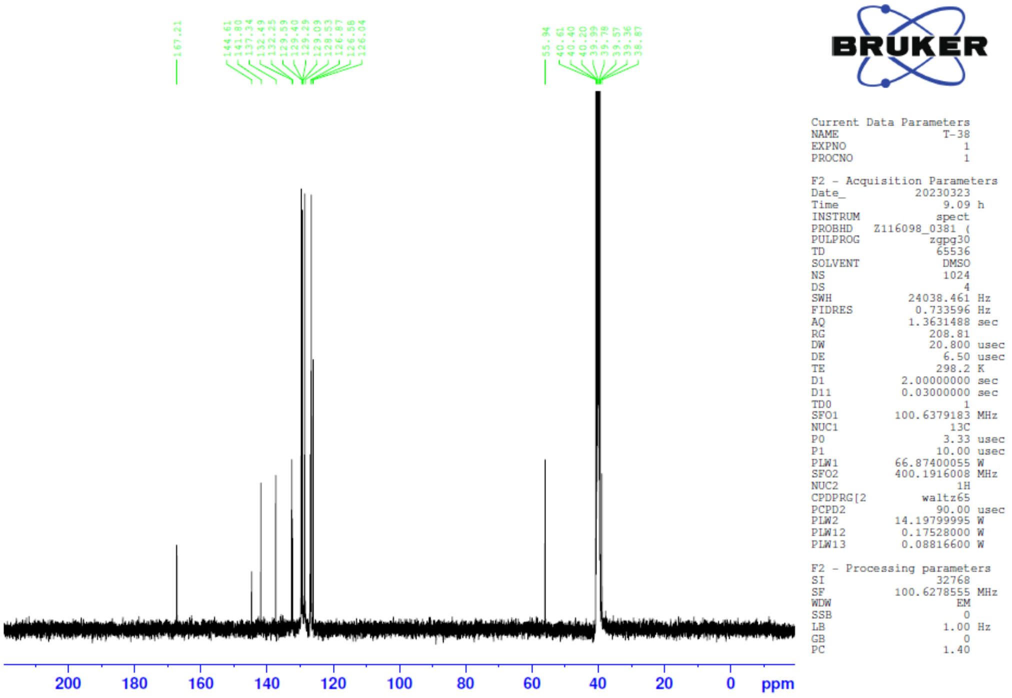

2-([1,1'-biphenyl]-4-sulfonamido)-*N*-hydroxy-3-phenylpropanamide (1n)

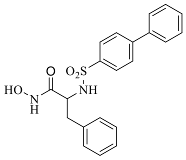

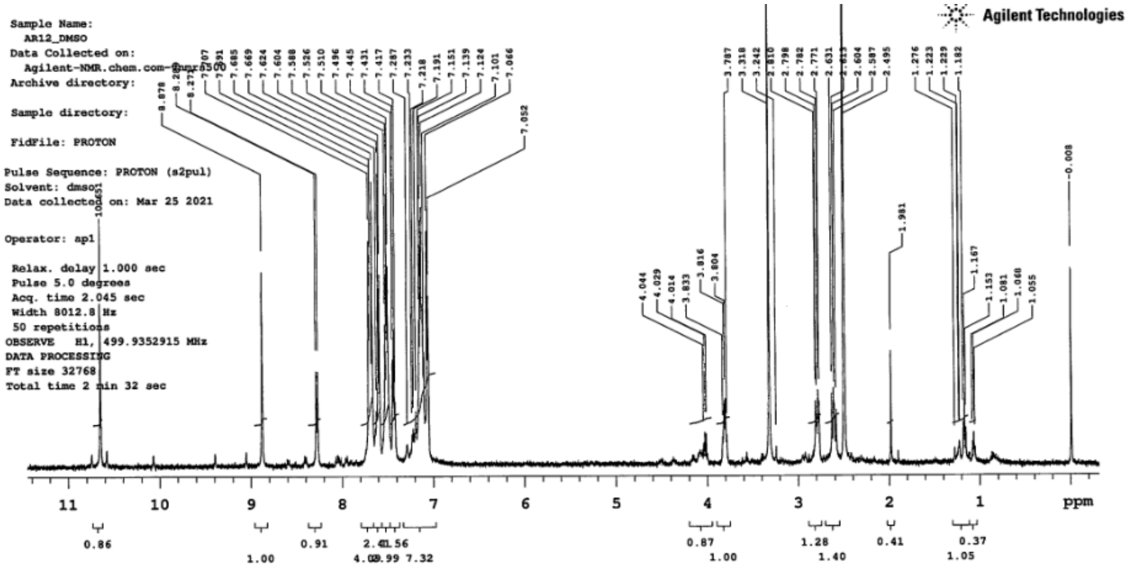

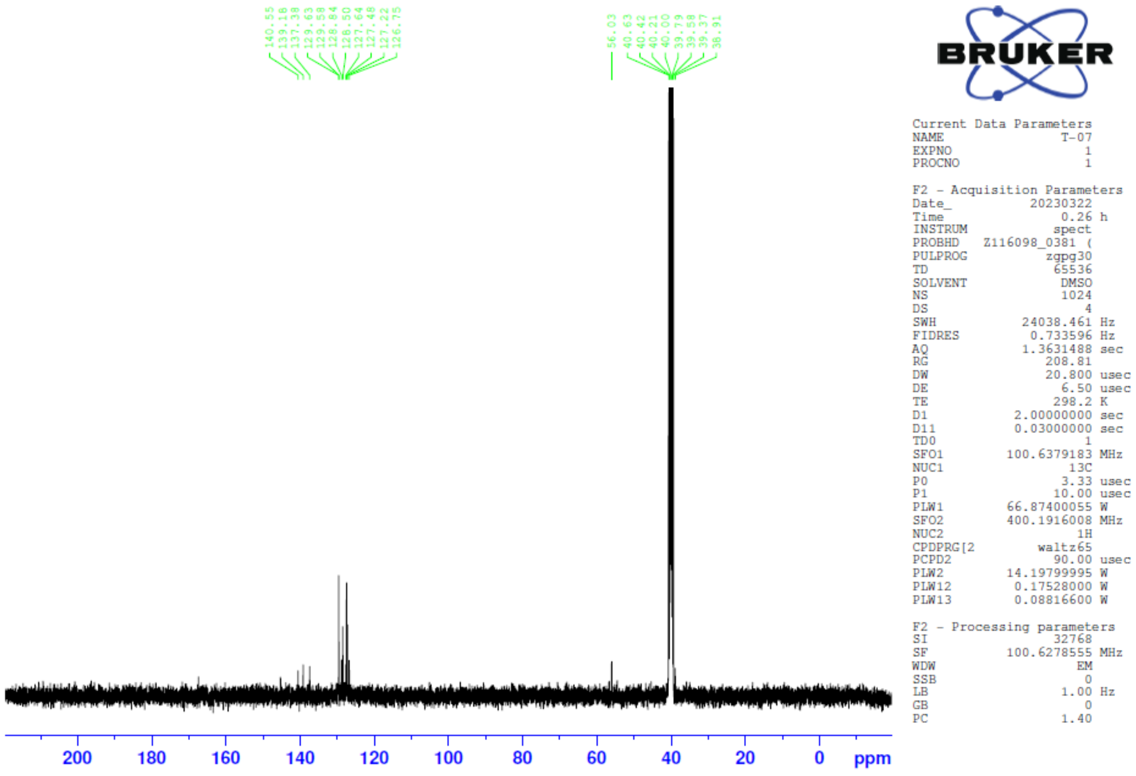

N-hydroxy-3-phenyl-2-((2,4,6-trimethylphenyl)sulfonamido)propanamide (1o)

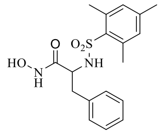

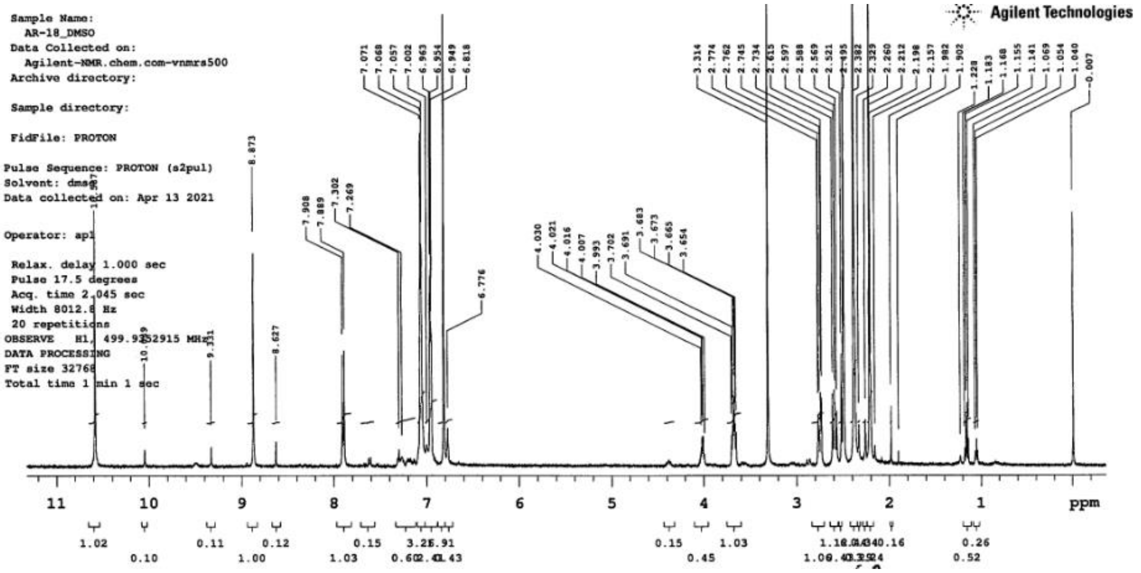

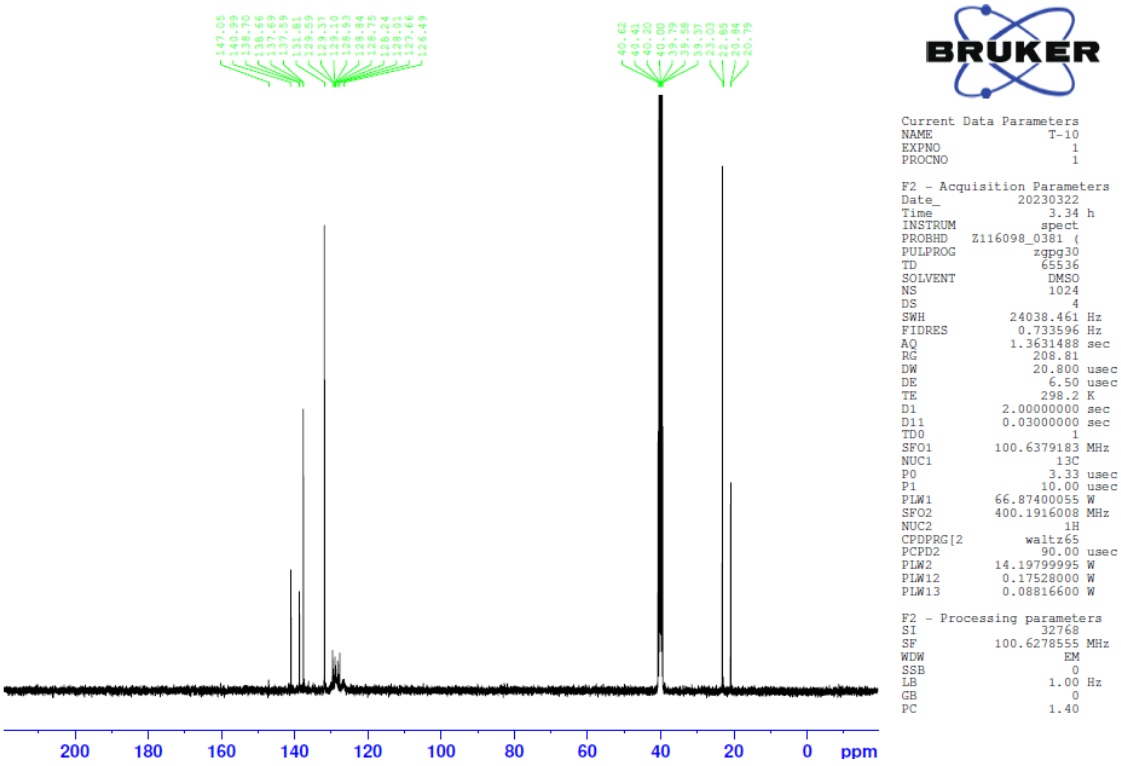

2-((4-butoxyphenyl)sulfonamido)-*N*-hydroxy-3-phenylpropanamide (1p)

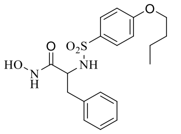

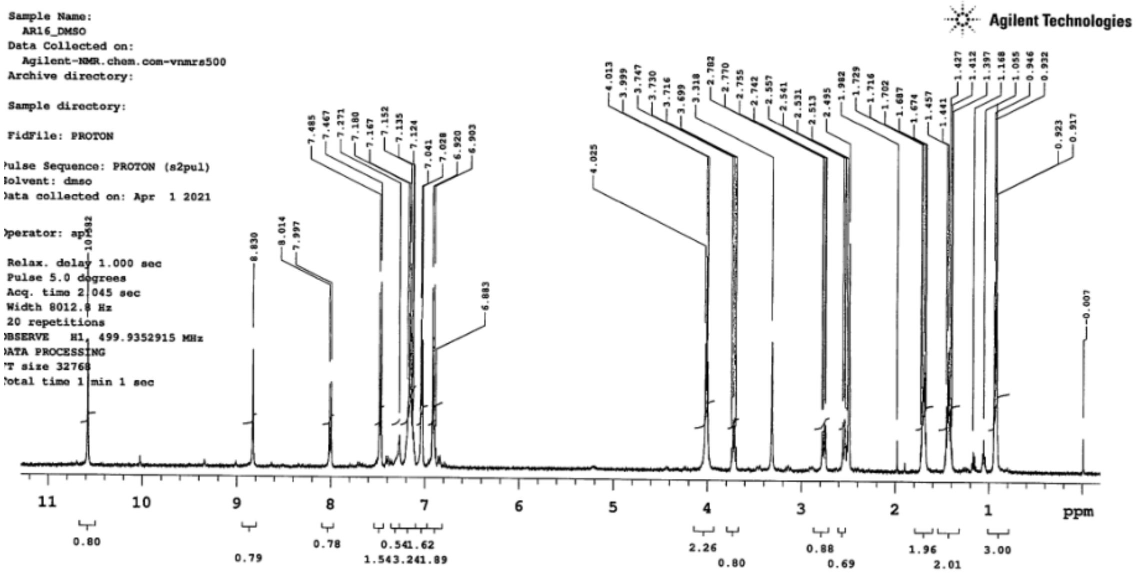

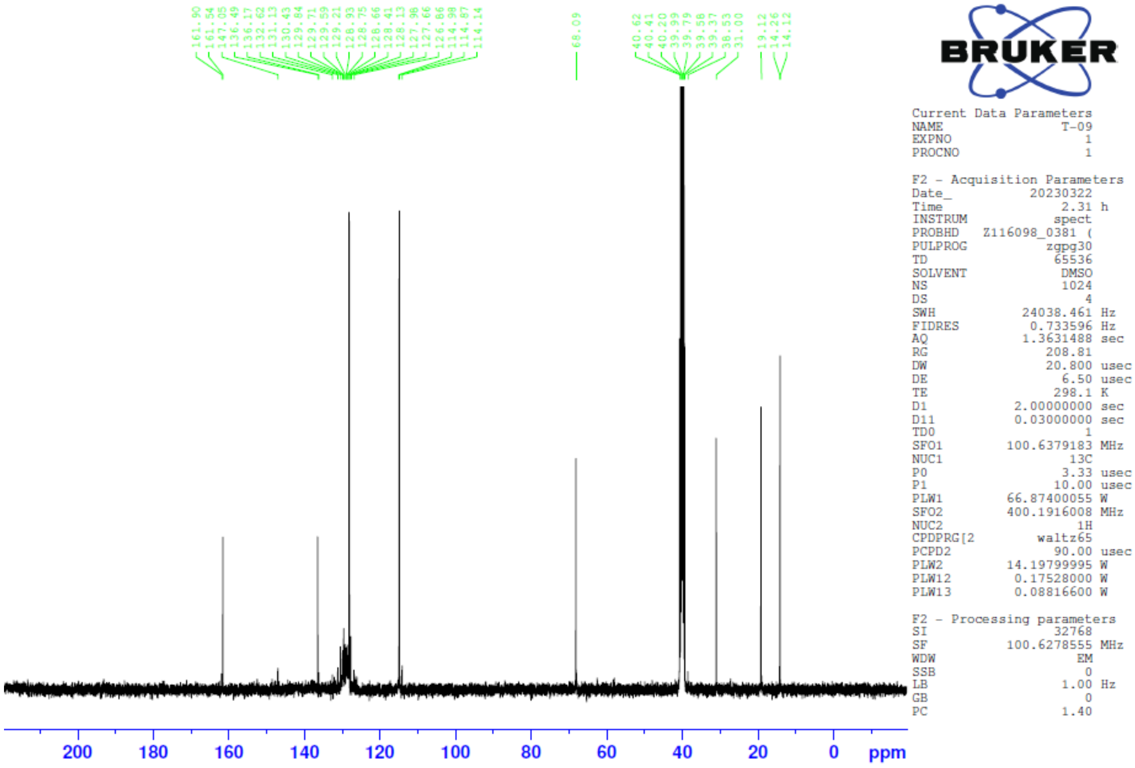

2-((4-(*tert*-butyl)phenyl)sulfonamido)-*N*-hydroxy-3-phenylpropanamide (1q)

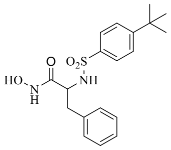

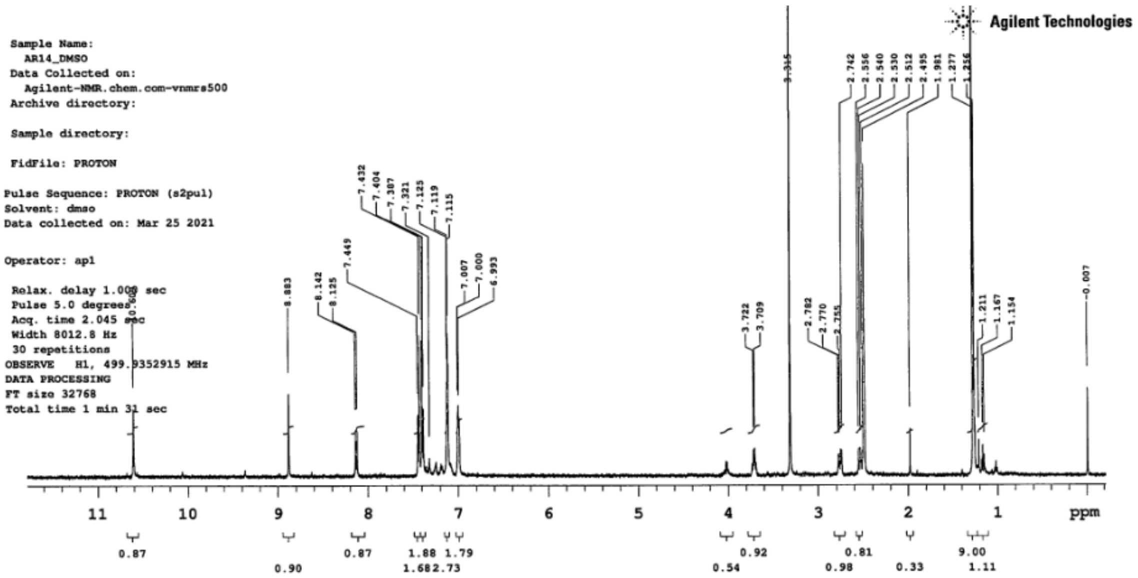

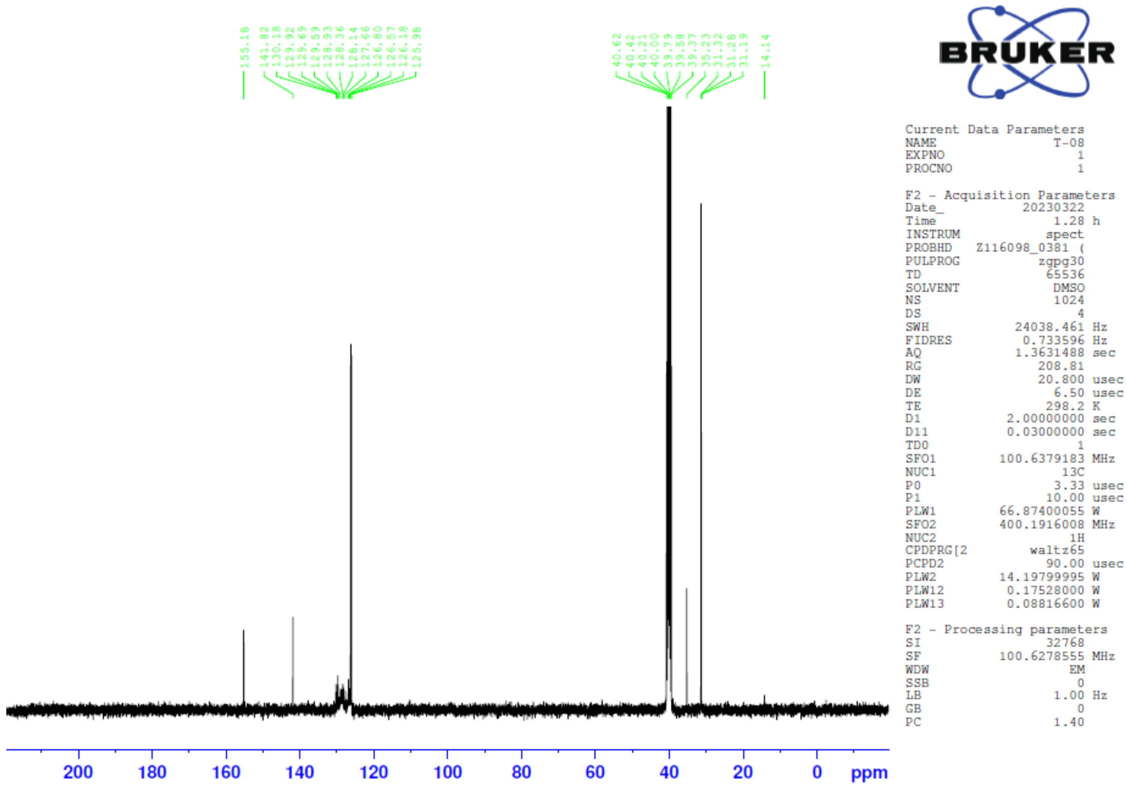

N-hydroxy-2-(naphthalene-2-sulfonamido)-3-phenylpropanamide (1r)

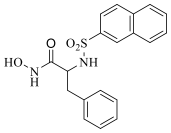

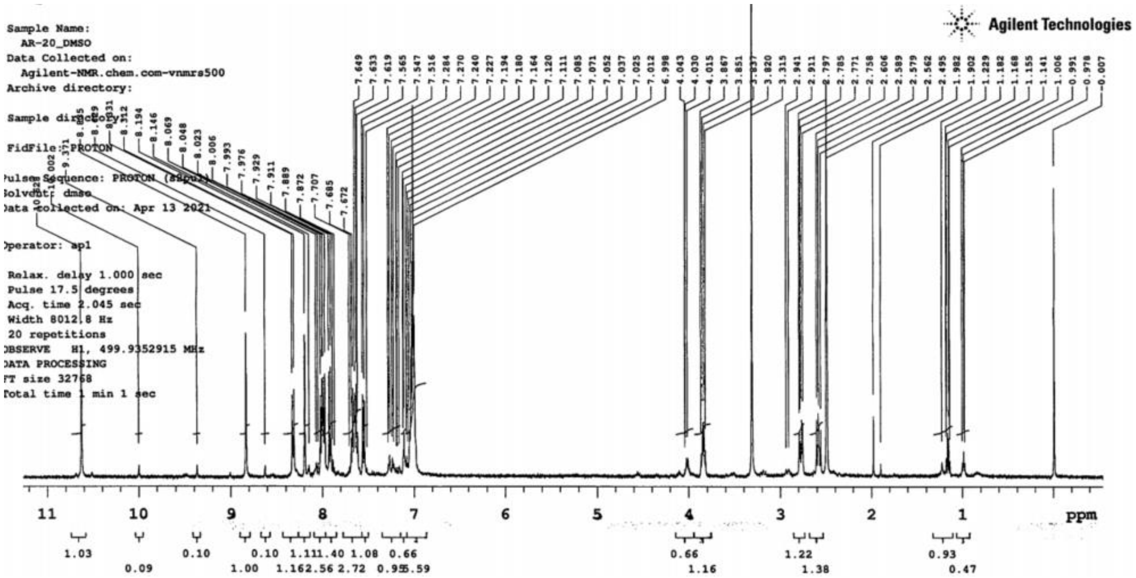

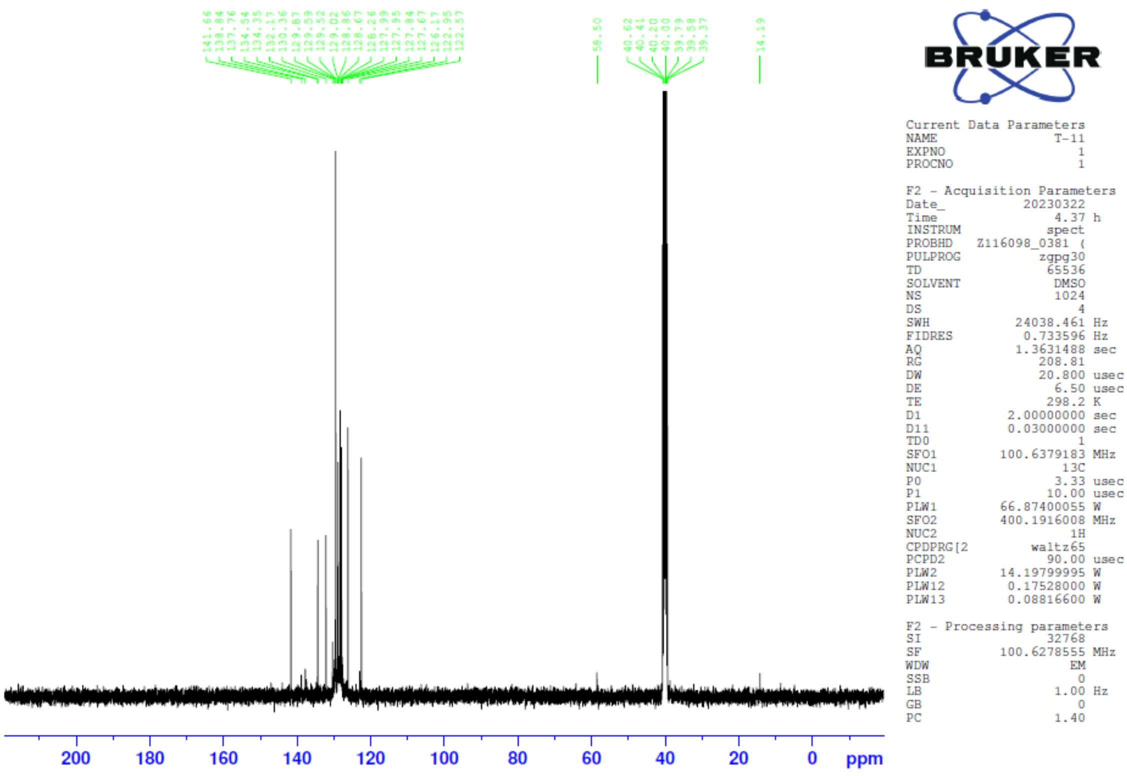

N-hydroxy-1-(phenylsulfonyl)pyrrolidine-2-carboxamide (1s)

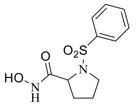

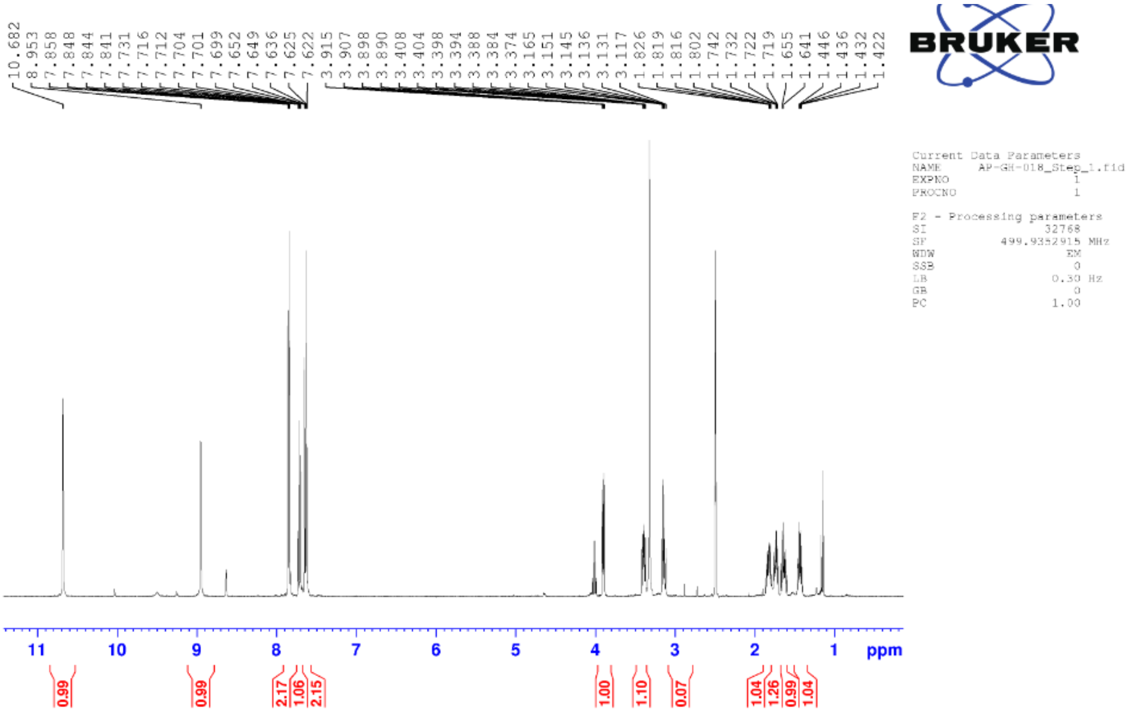

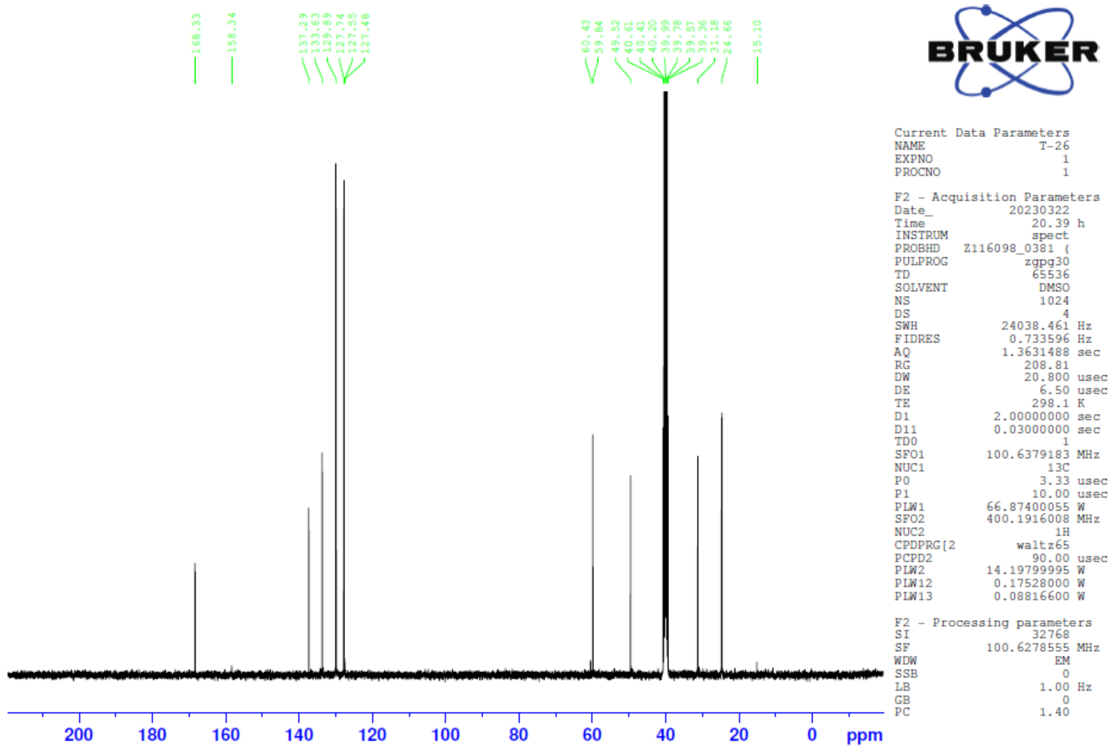

1-([1,1'-biphenyl]-4-ylsulfonyl)-*N*-hydroxypyrrolidine-2-carboxamide (1t)

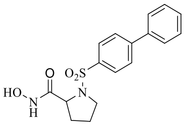

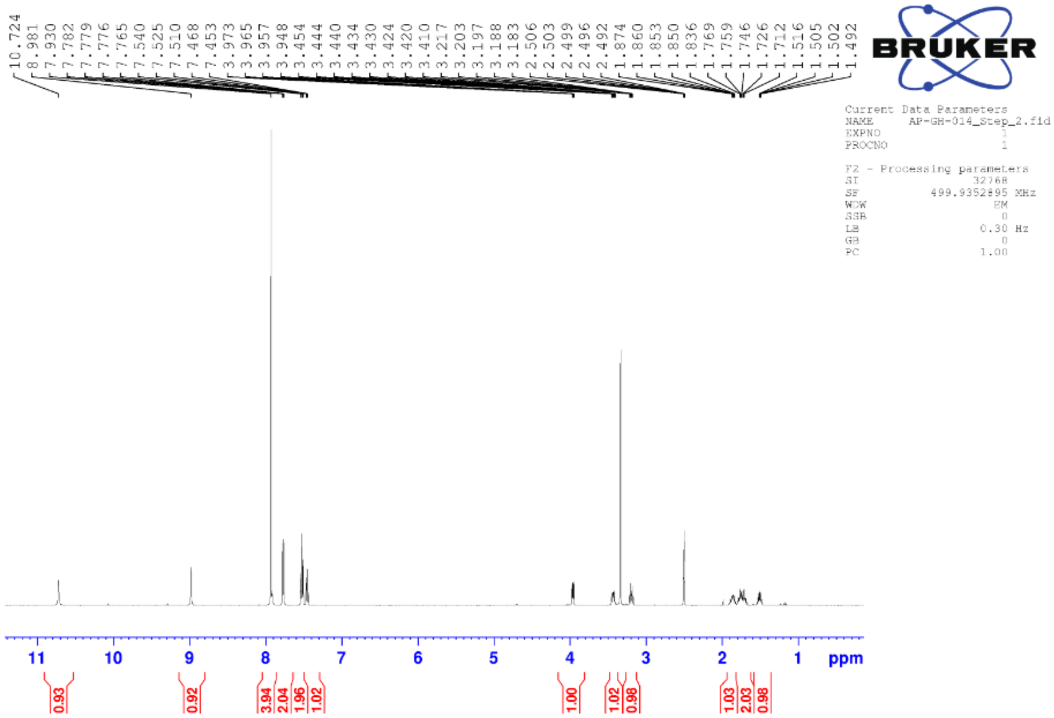

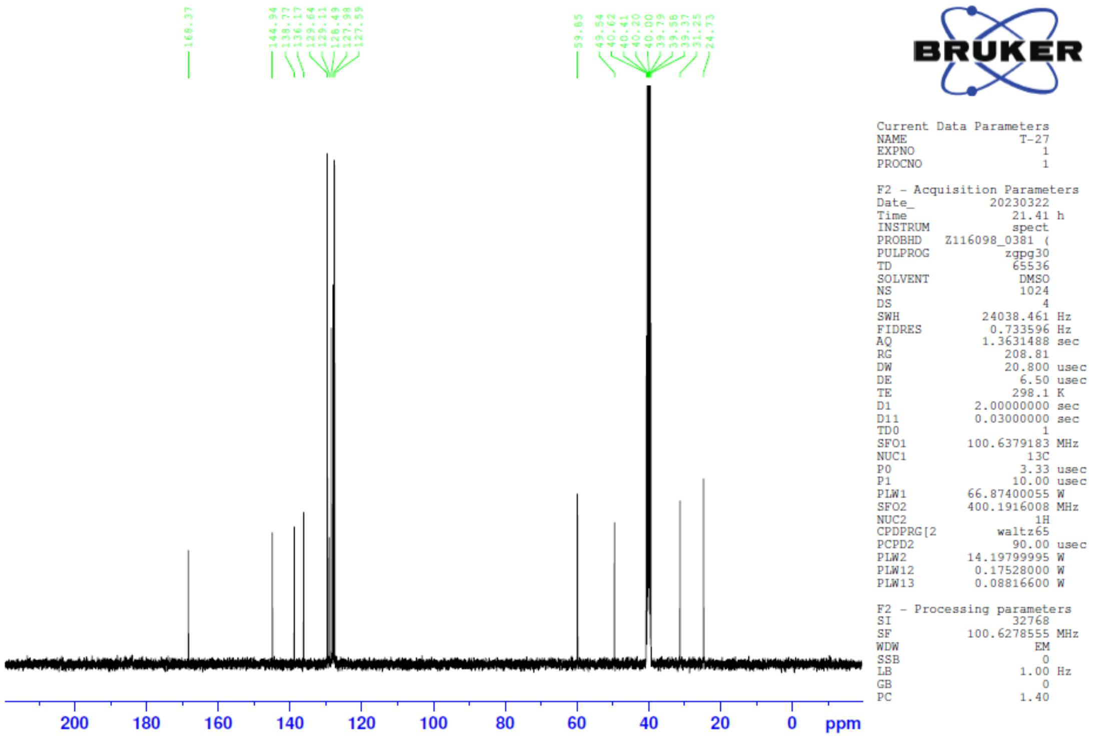

1-((4-(*tert*-butyl)phenyl)sulfonyl)-*N*-hydroxypyrrolidine-2-carboxamide (1u)

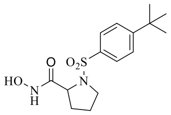

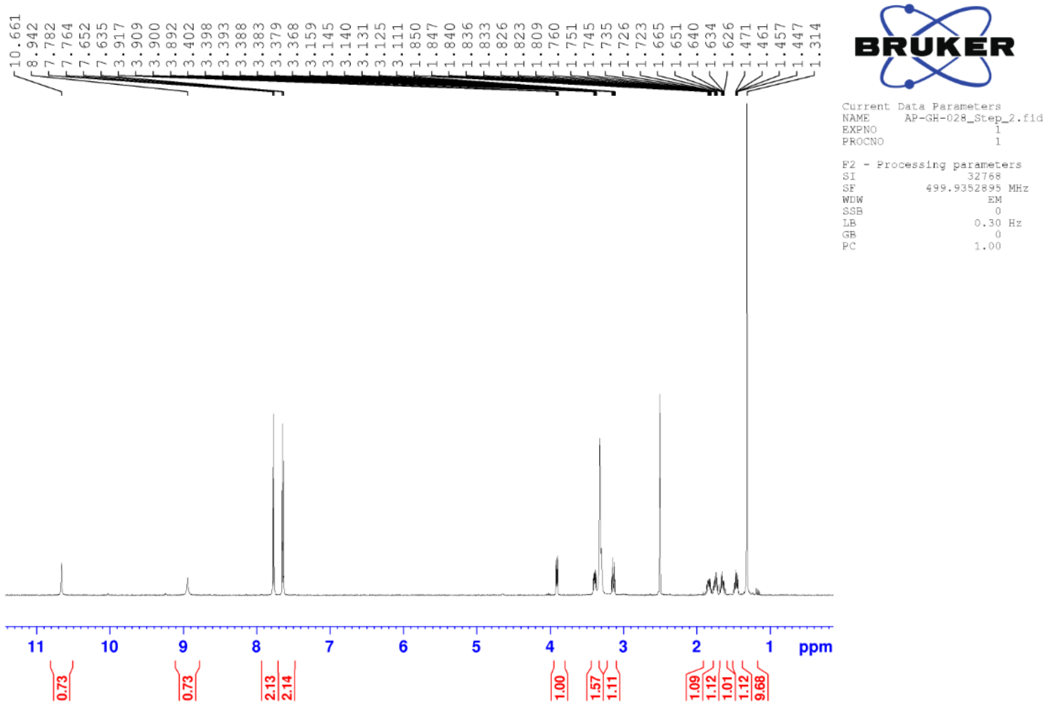

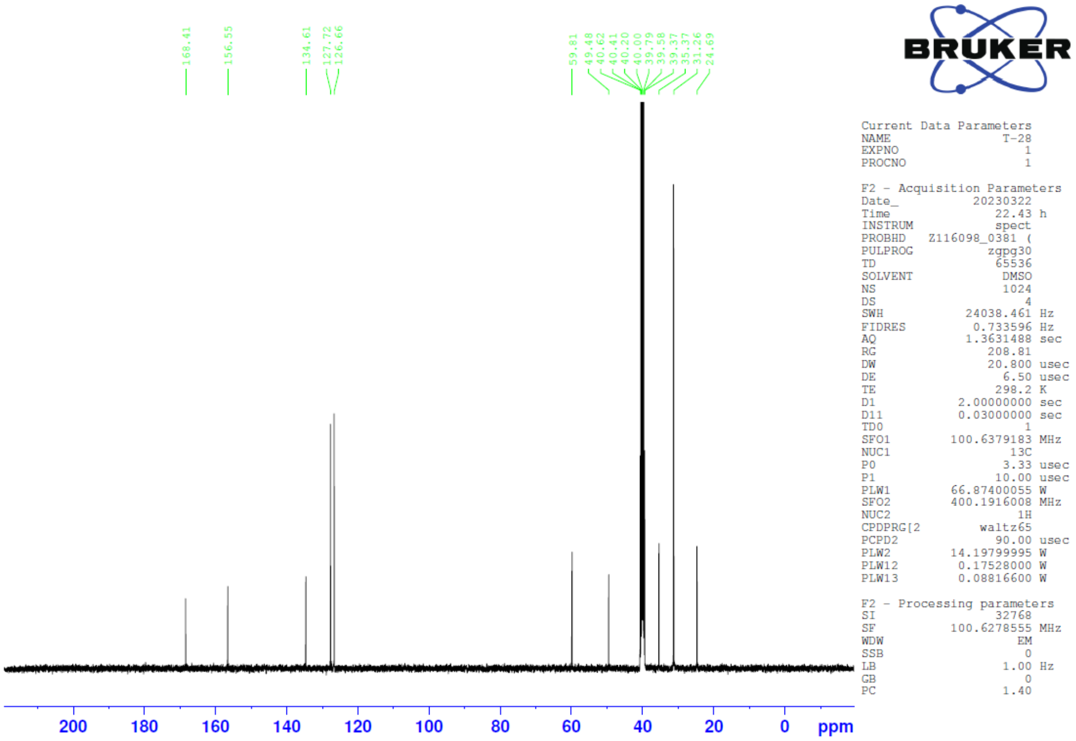

1-((4-butoxyphenyl)sulfonyl)-*N*-hydroxypyrrolidine-2-carboxamide (1v)

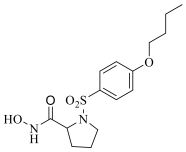

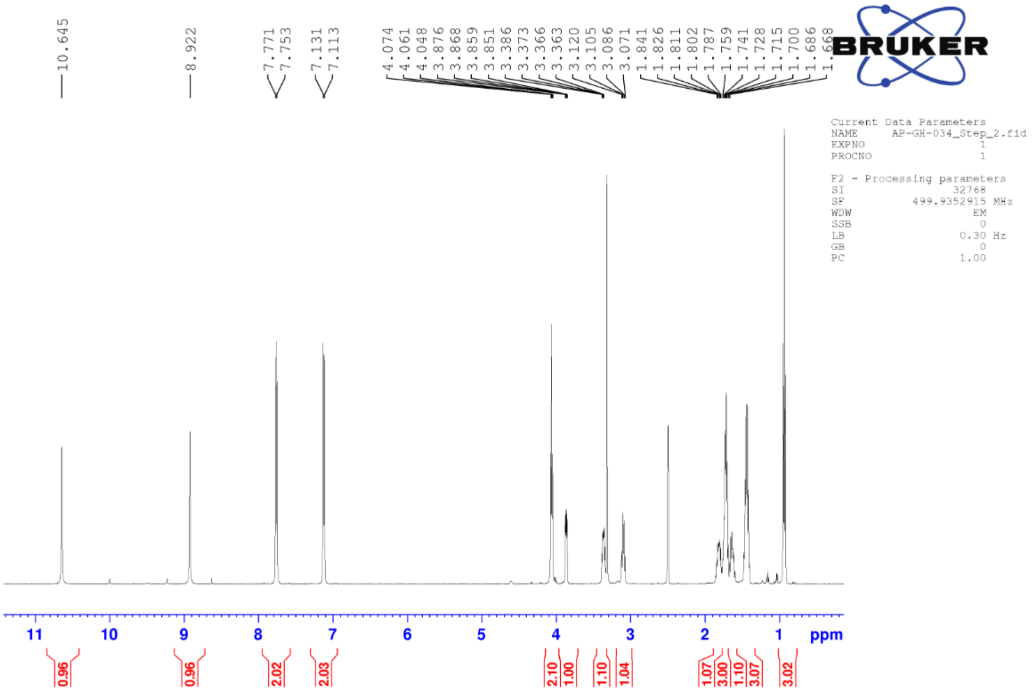

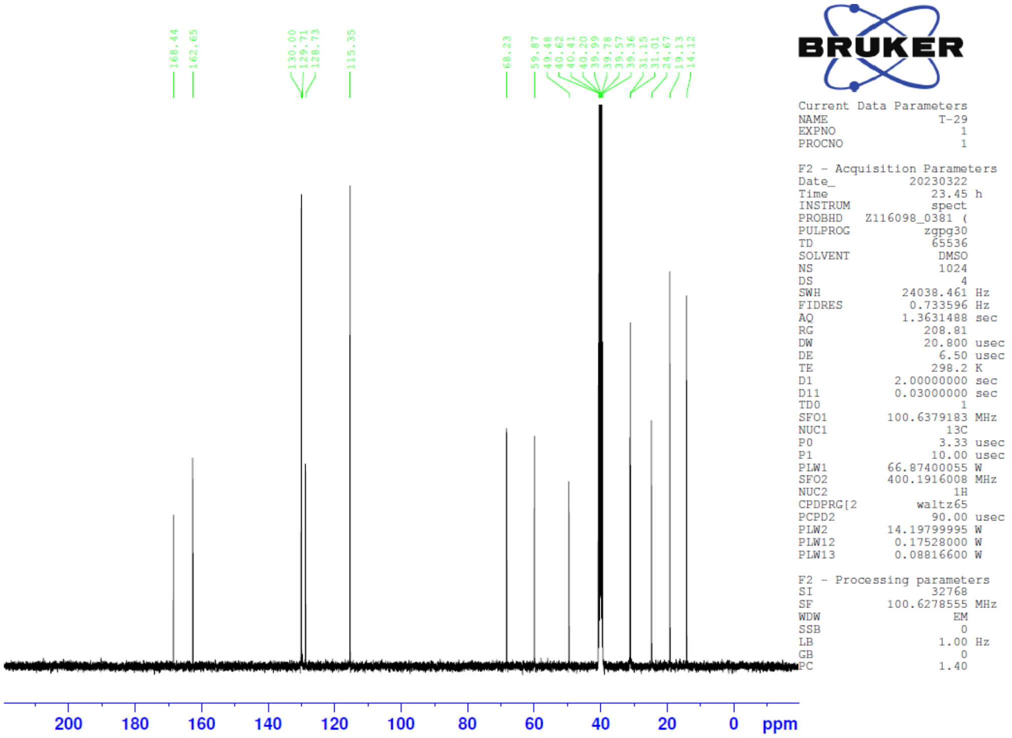

1-((4-fluorophenyl)sulfonyl)-*N*-hydroxypyrrolidine-2-carboxamide (1w)

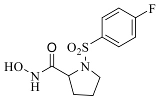

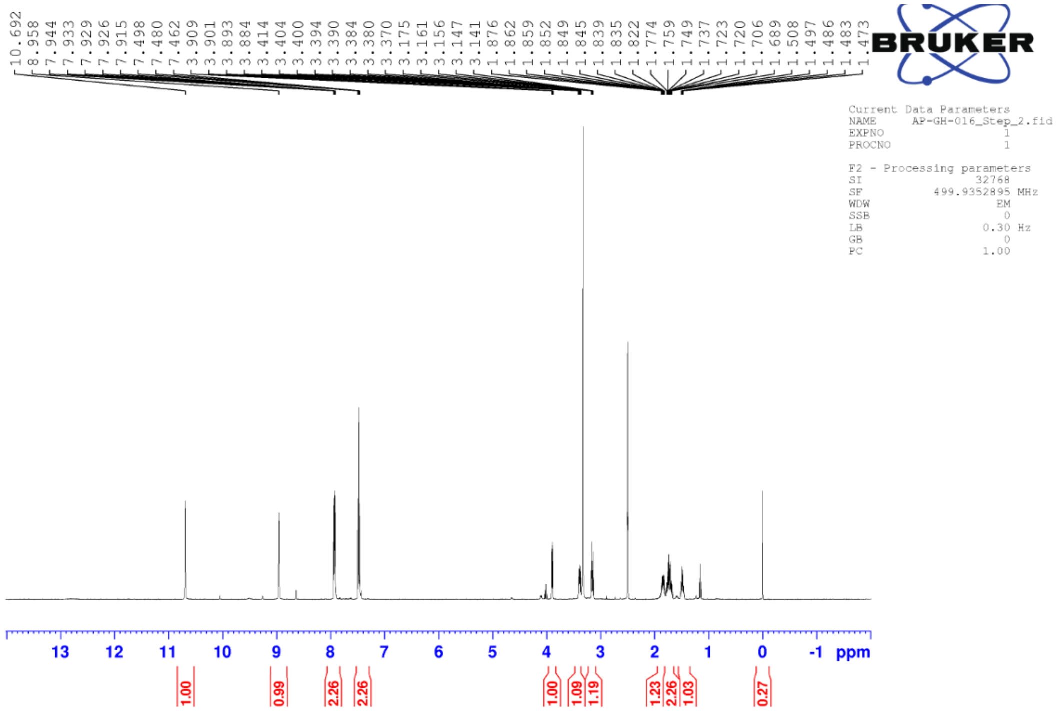

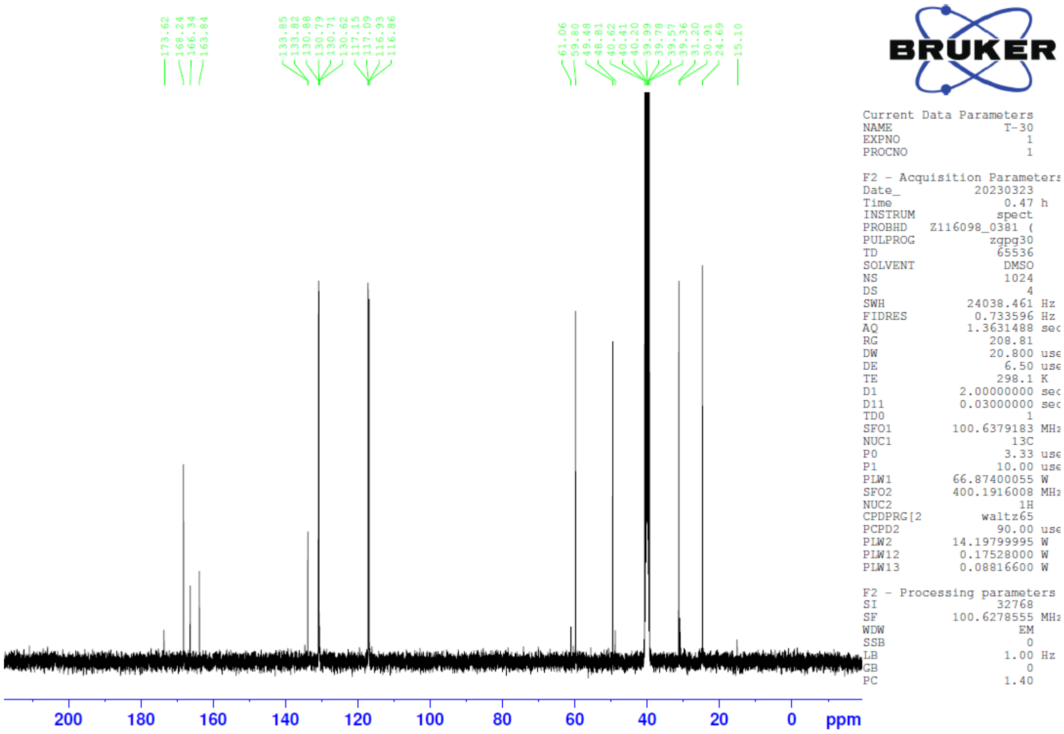

N-hydroxy-1-tosylpyrrolidine-2-carboxamide (1x):

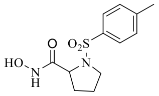

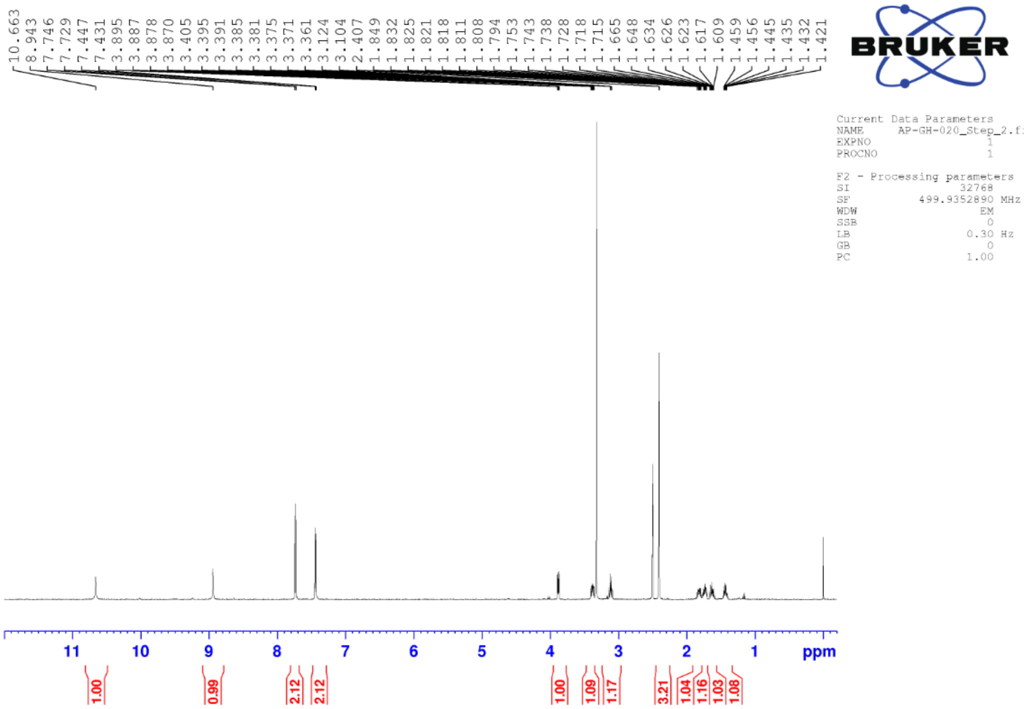

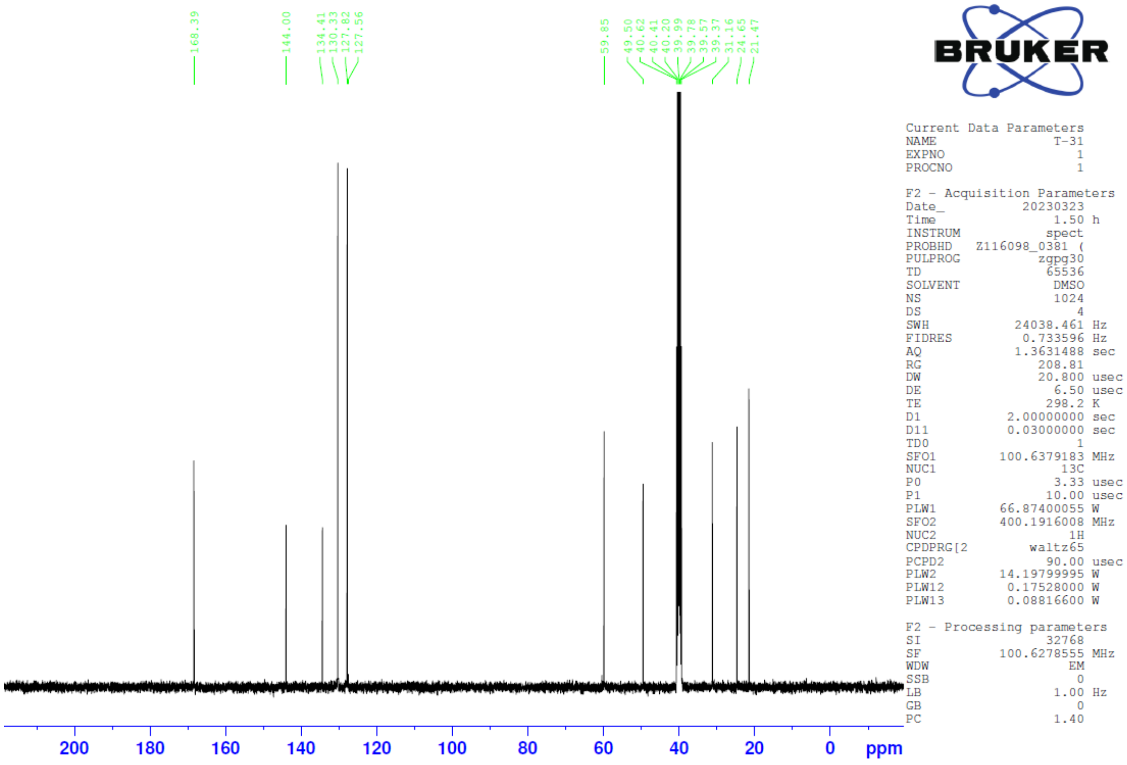

N-hydroxy-1-((4-propylphenyl)sulfonyl)pyrrolidine-2-carboxamide (1y)

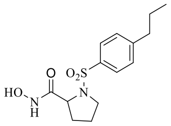

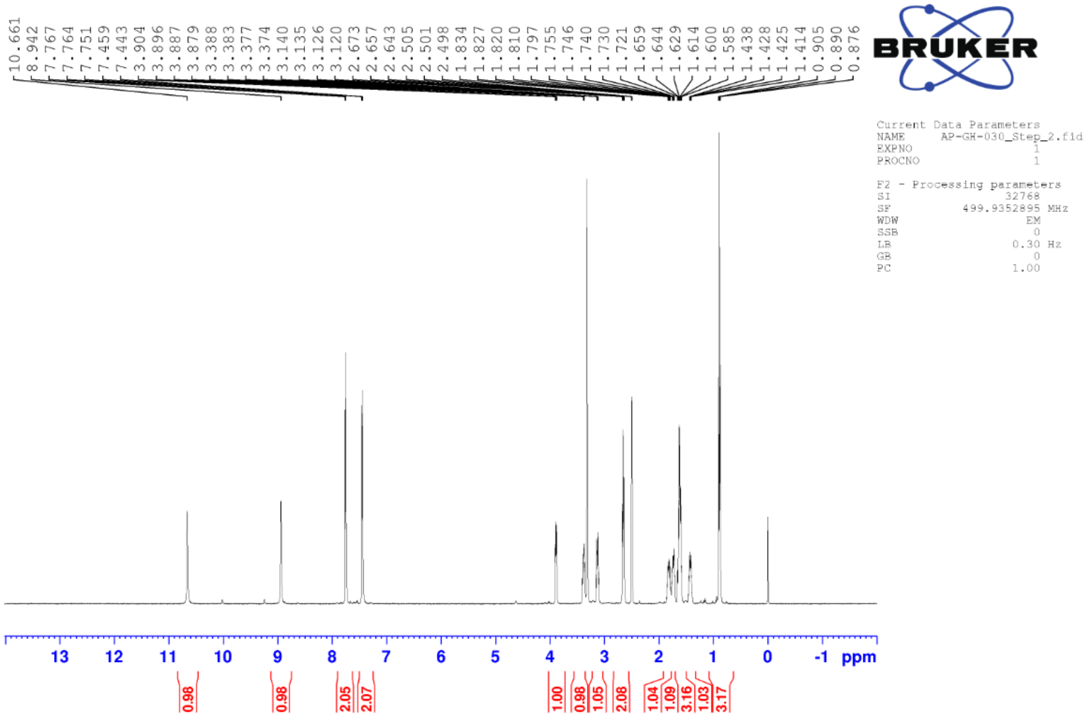

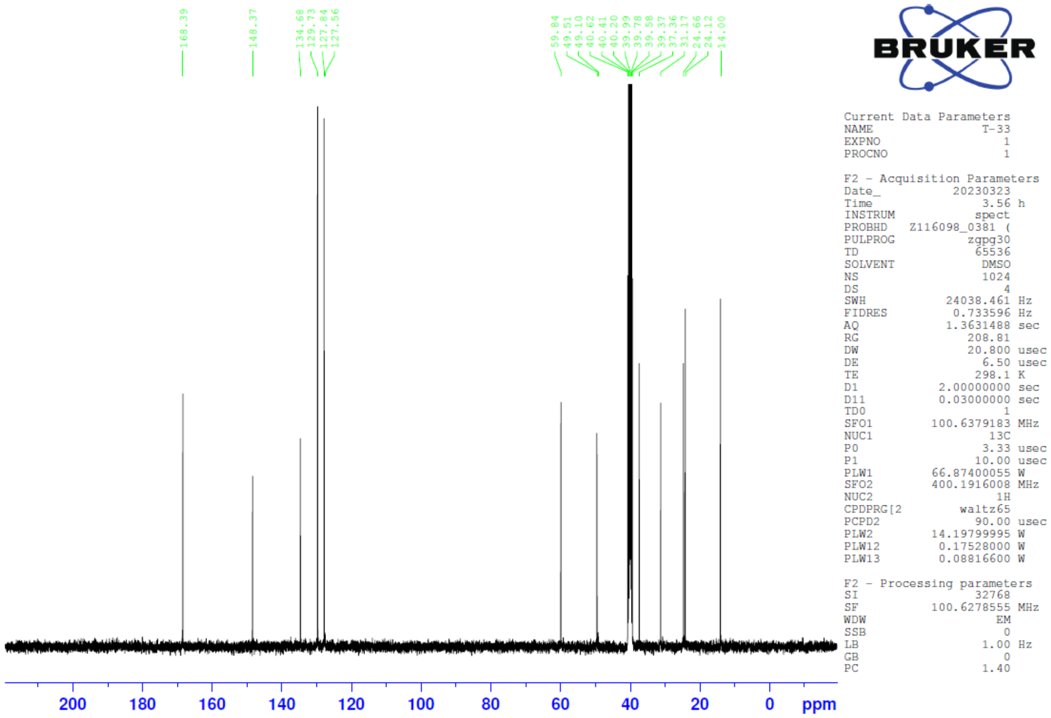

1-((4-((3*r*,5*r*,7*r*)-adamantan-1-yl)phenyl)sulfonyl)-*N*-hydroxypyrrolidine-2-carboxamide (1z)

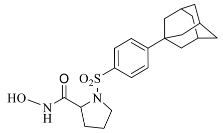

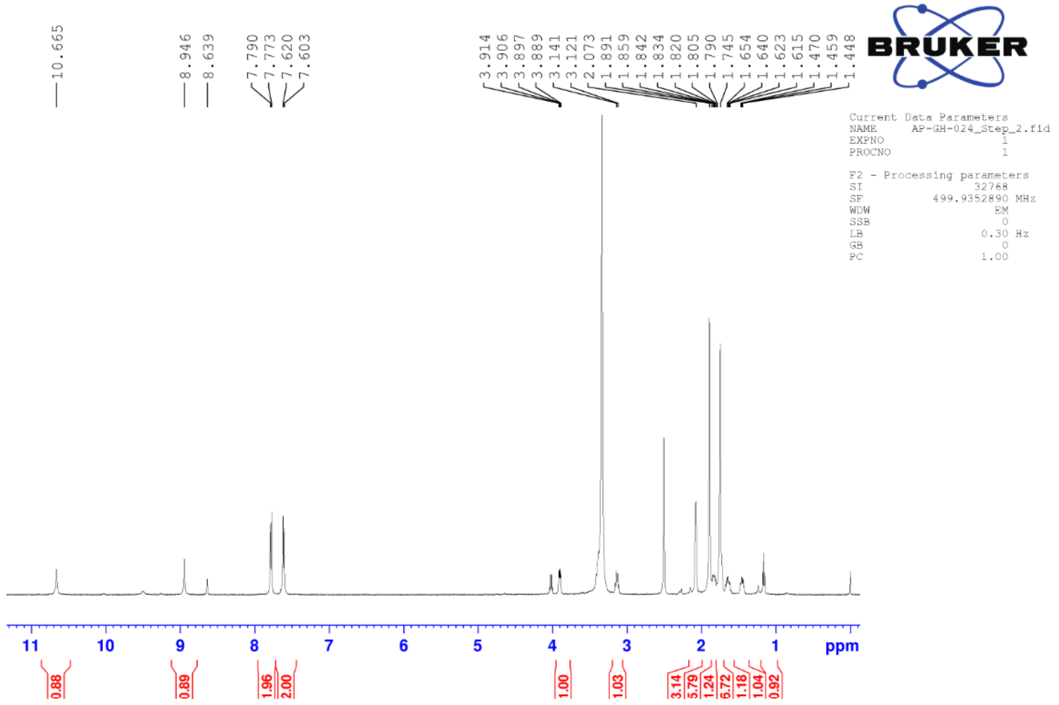

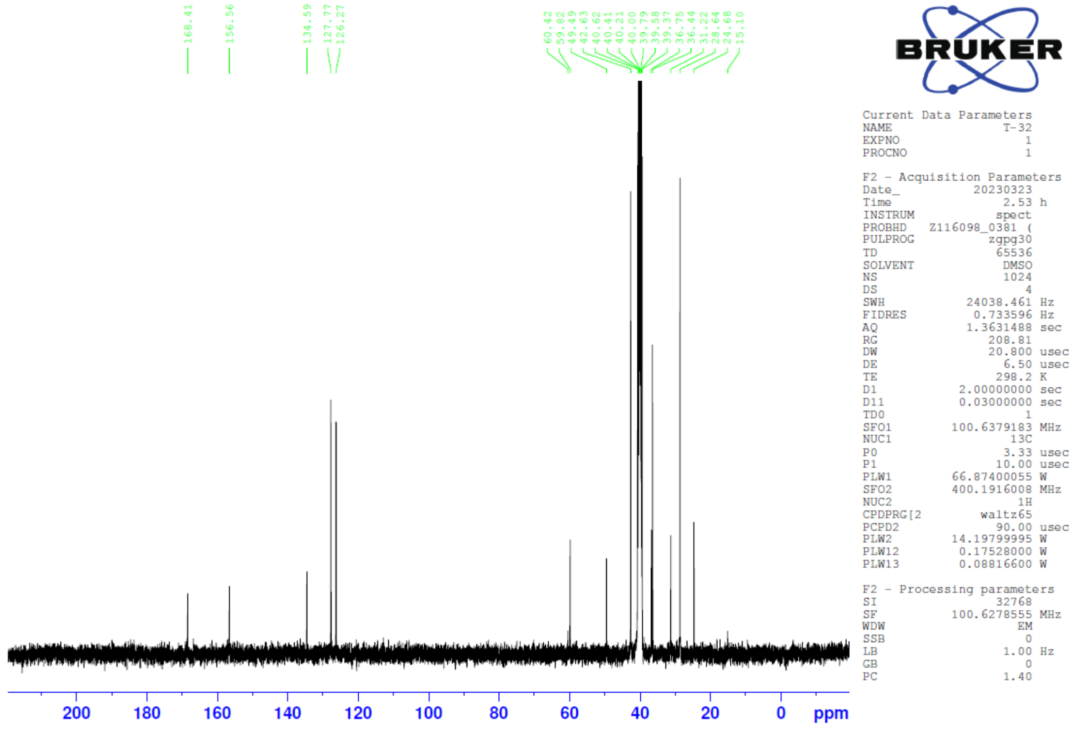

1-((4-acetamidophenyl)sulfonyl)-*N*-hydroxypyrrolidine-2-carboxamide (1aa)

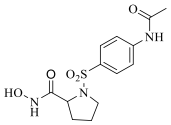

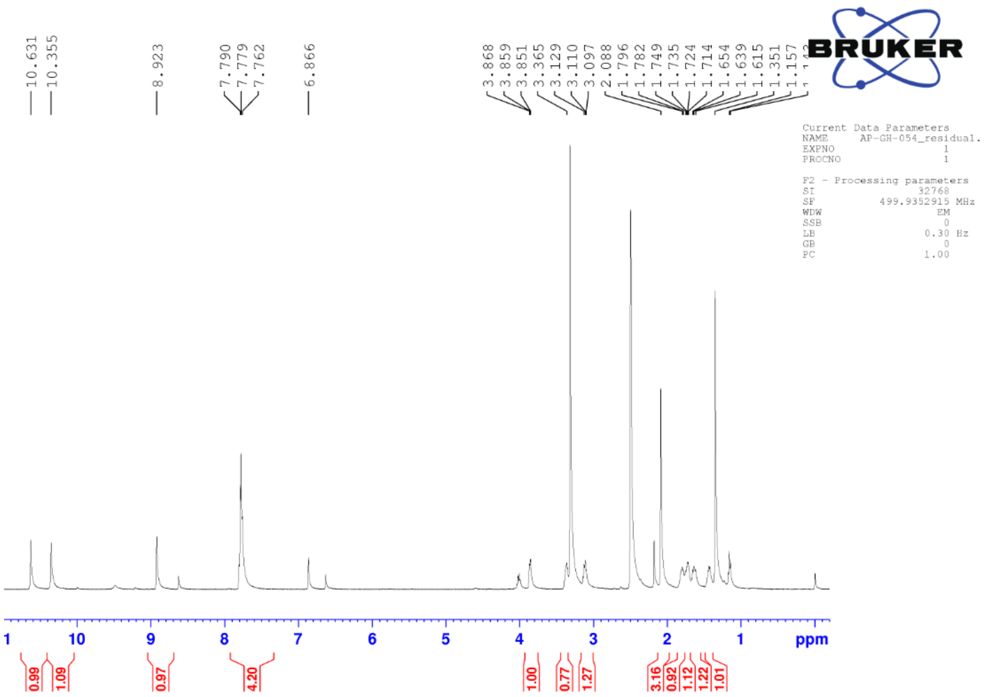

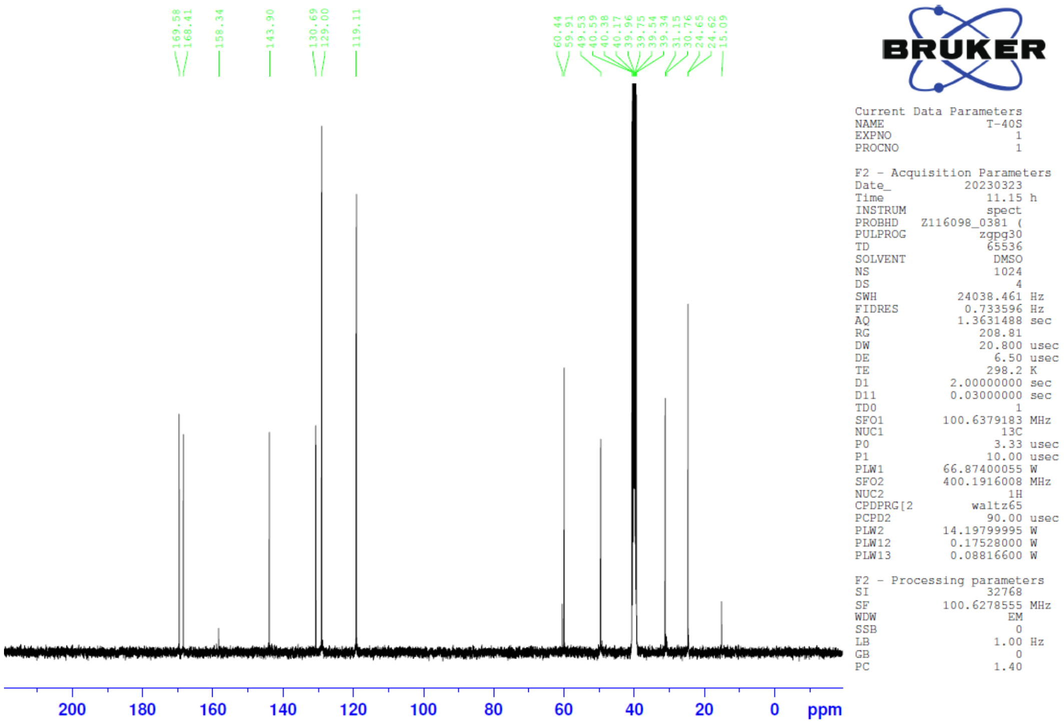

1-((4-acetylphenyl)sulfonyl)-*N*-hydroxypyrrolidine-2-carboxamide (1ab)

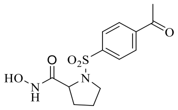

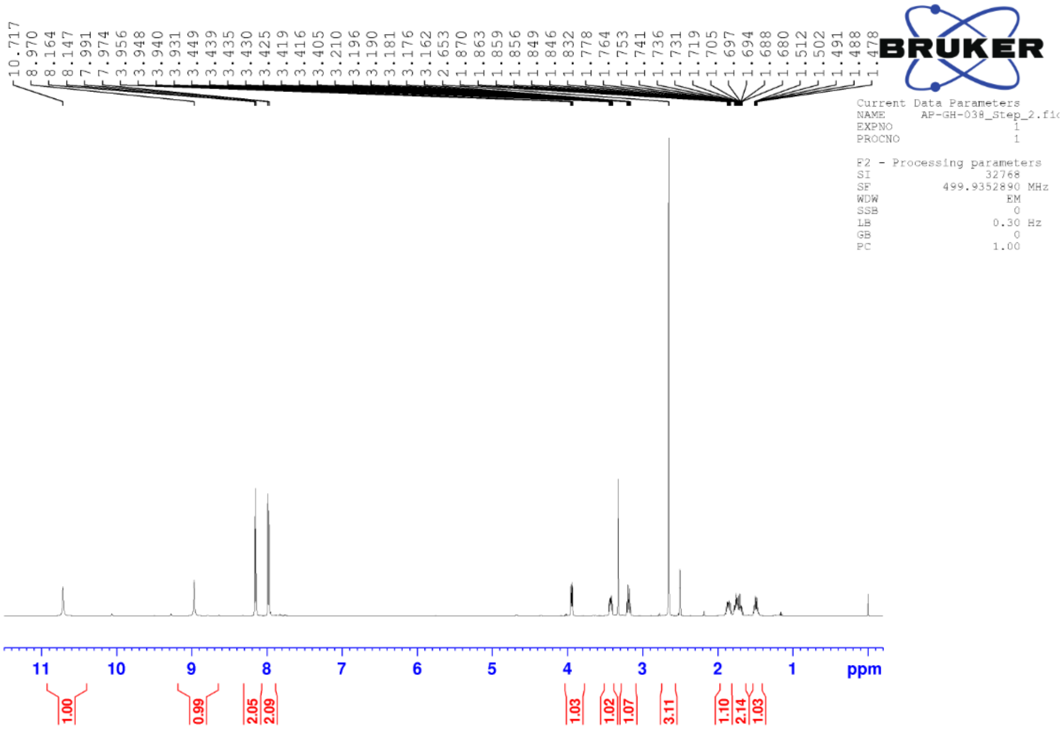

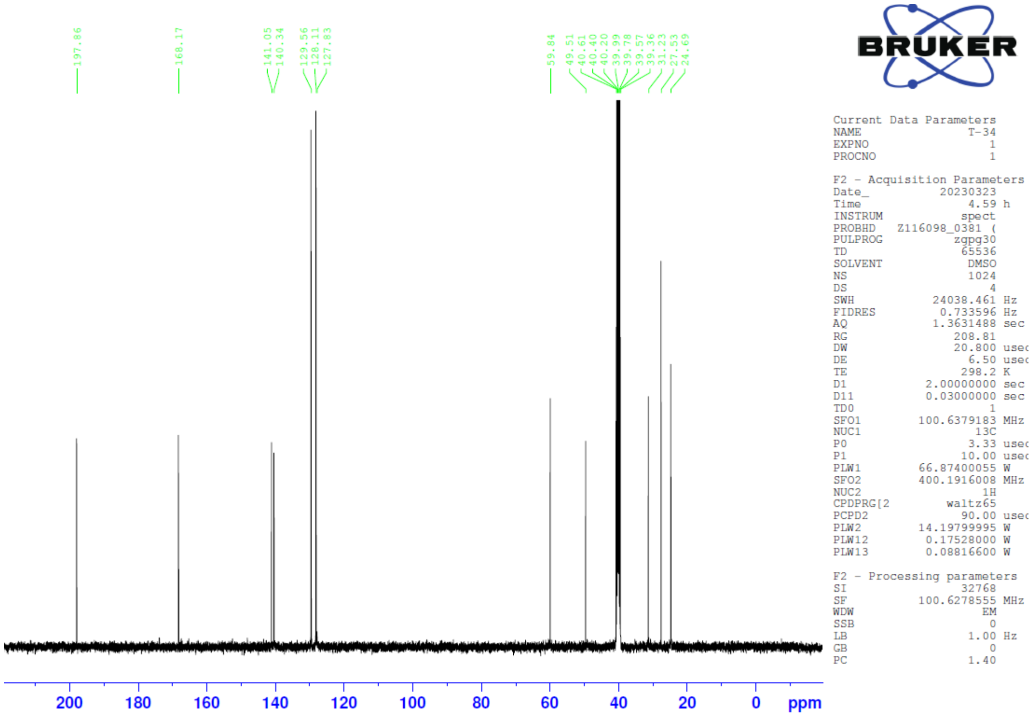

N-hydroxy-1-(phenylsulfonyl)piperidine-2-carboxamide (1ac)

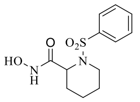

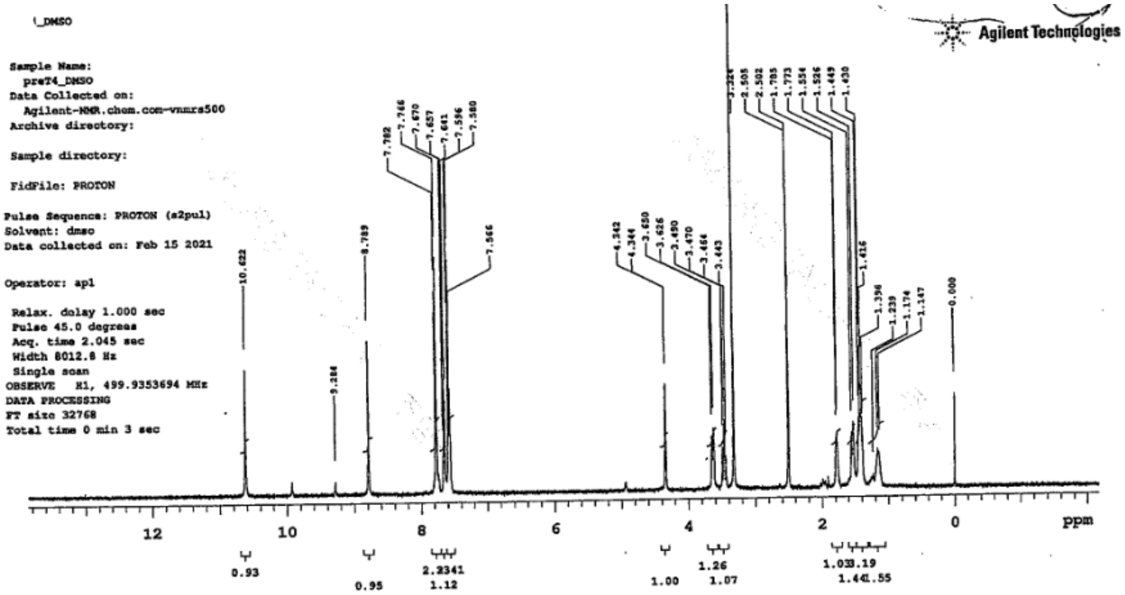

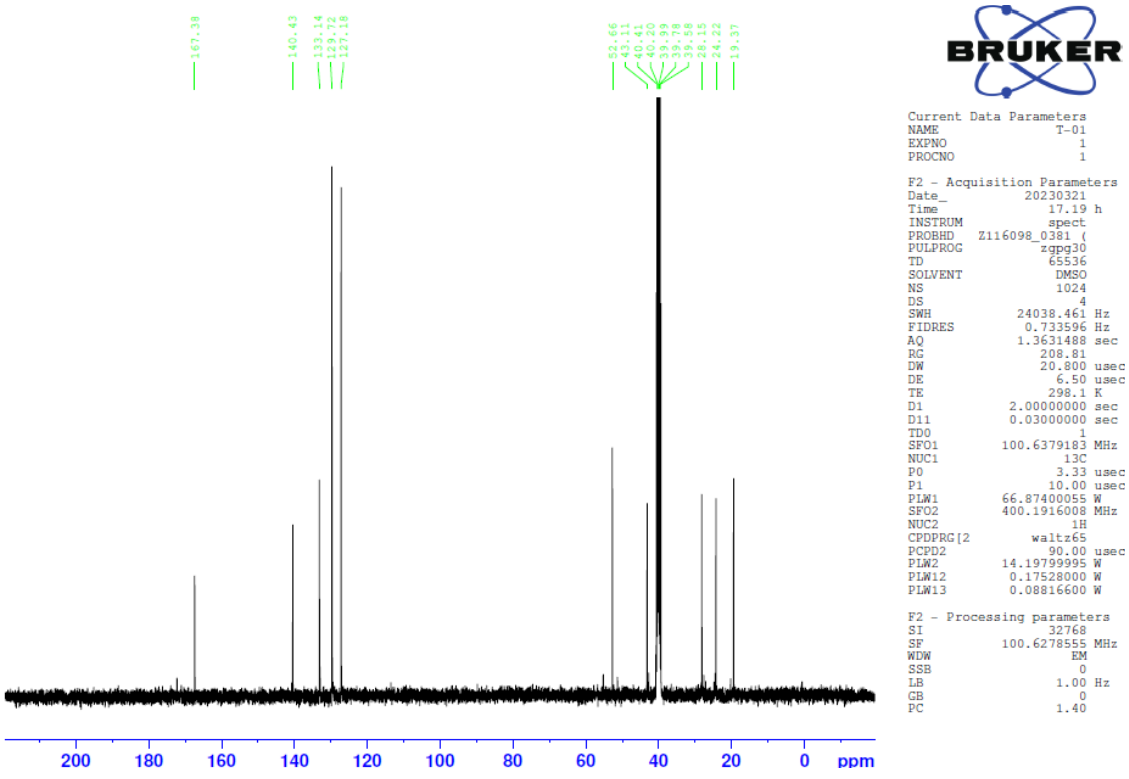

1-([1,1'-biphenyl]-4-ylsulfonyl)-*N*-hydroxypiperidine-2-carboxamide (1ad)

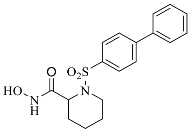

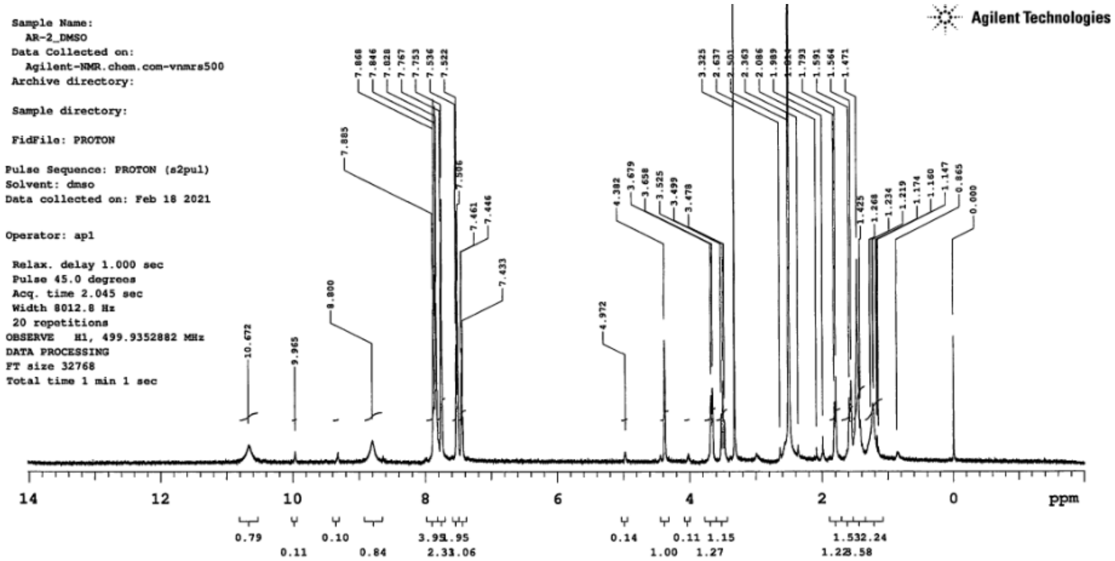

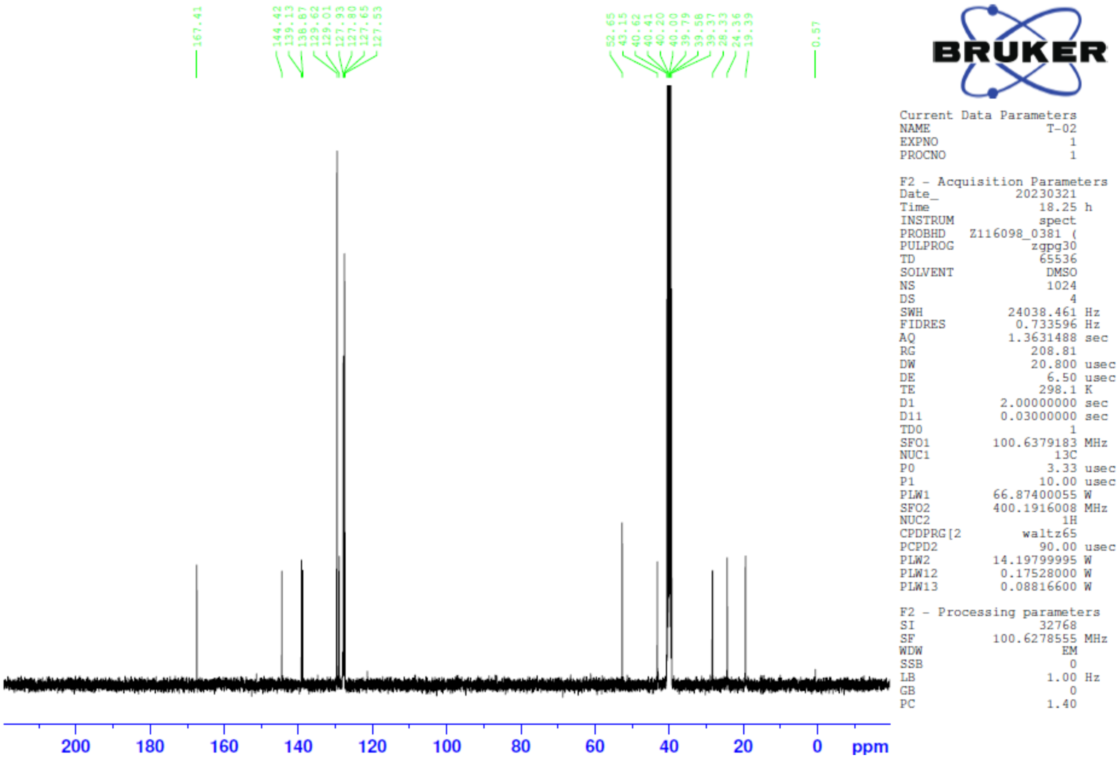

N-hydroxy-1-(mesitylsulfonyl)piperidine-2-carboxamide (1ae)

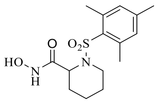

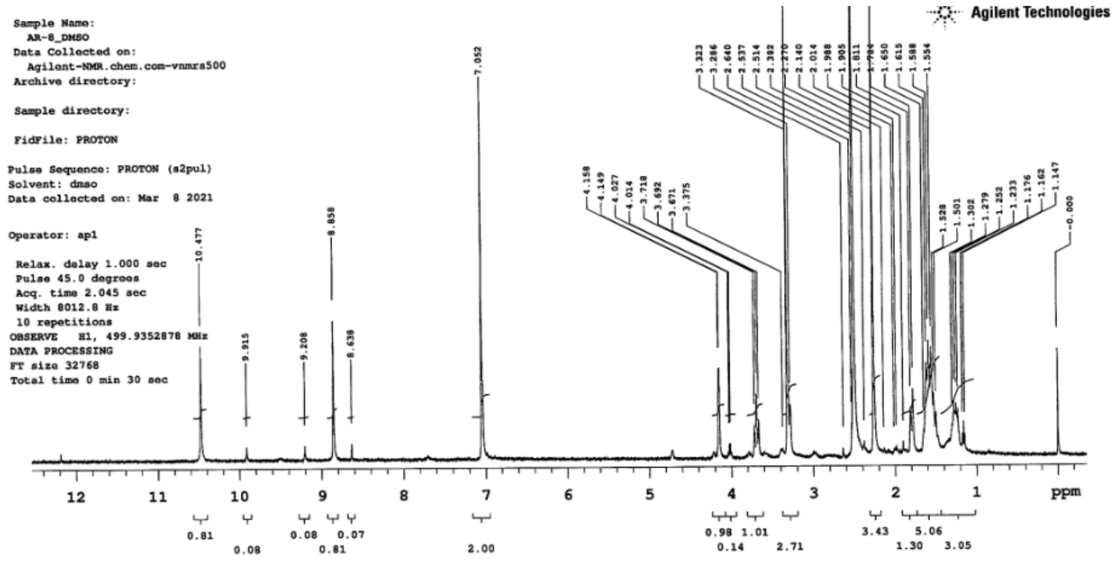

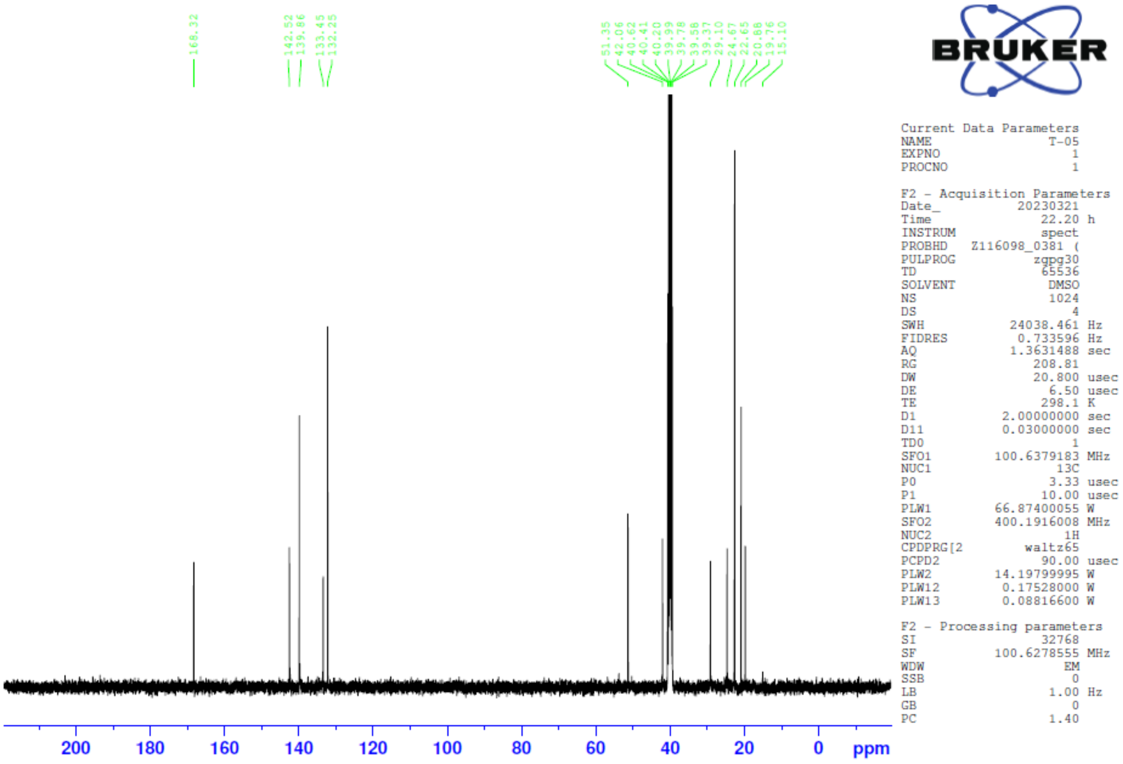

1-((4-butoxyphenyl)sulfonyl)-*N*-hydroxypiperidine-2-carboxamide (1af)

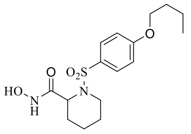

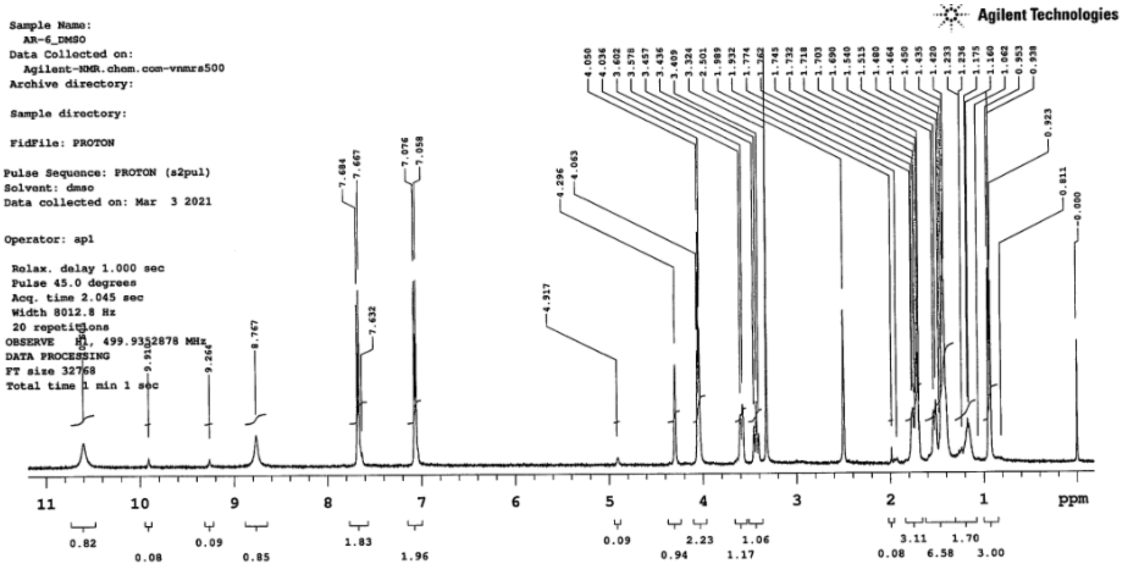

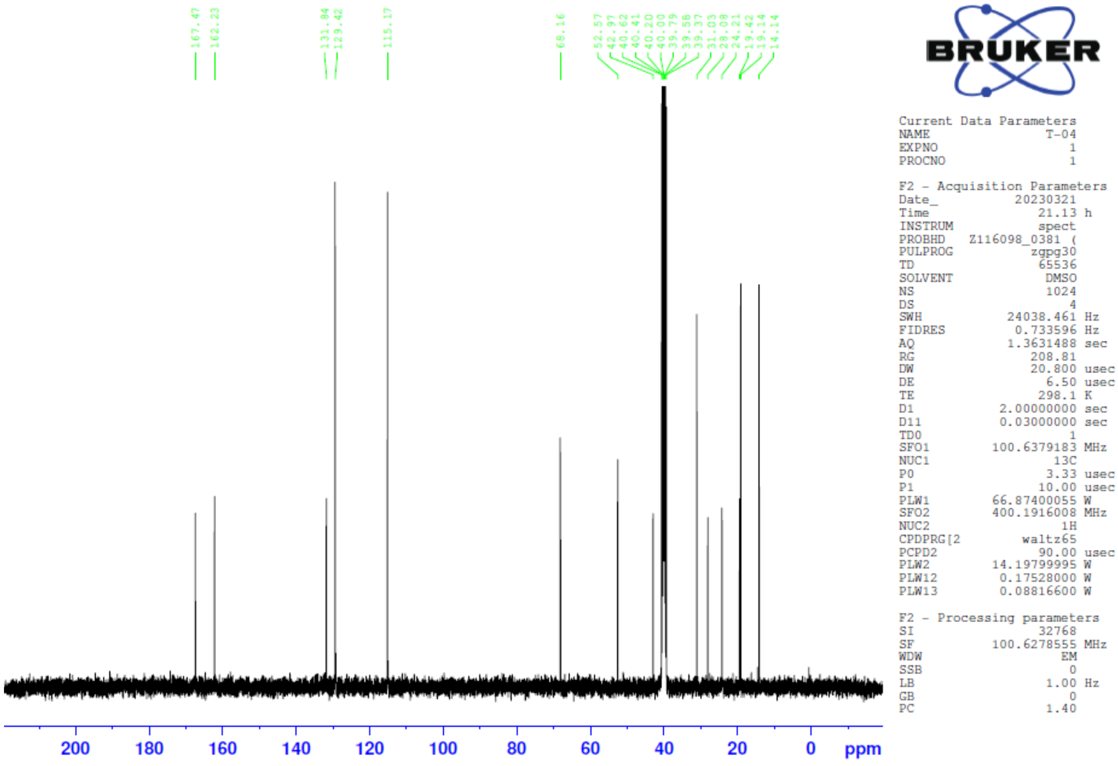

1-((4-(*tert*-butyl)phenyl)sulfonyl)-*N*-hydroxypiperidine-2-carboxamide (1ag)

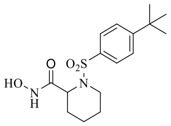

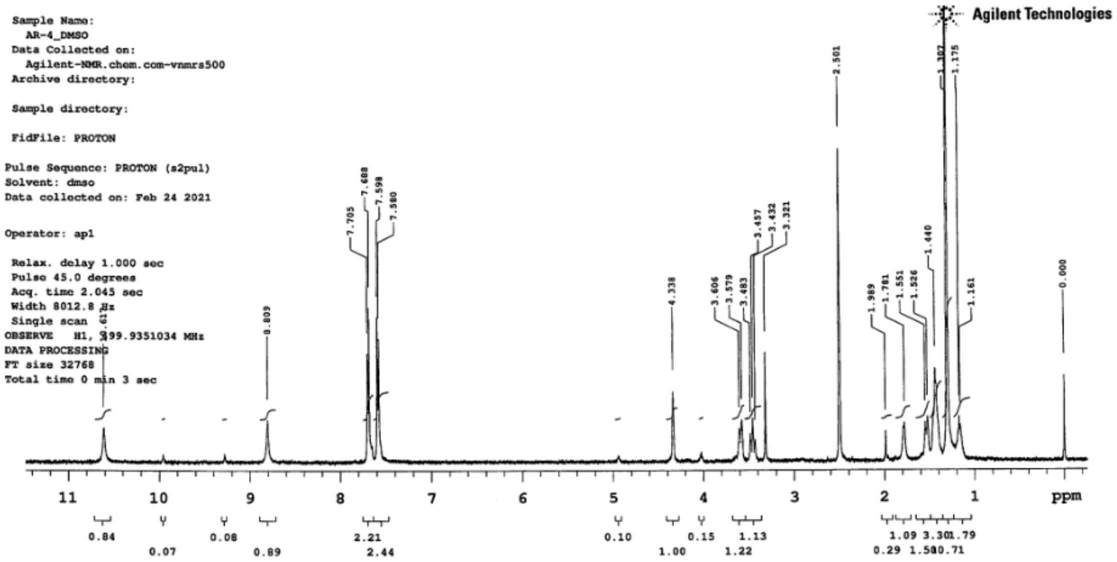

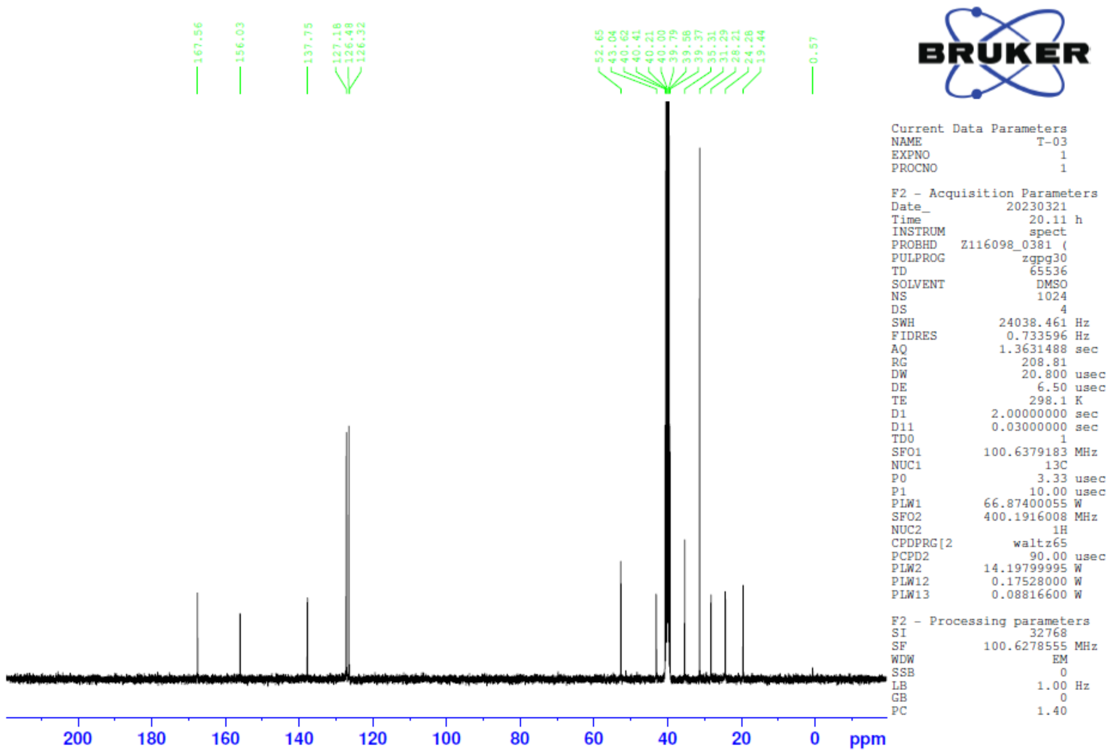

N-hydroxy-1-(naphthalen-2-ylsulfonyl)piperidine-2-carboxamide (1ah)

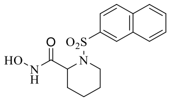

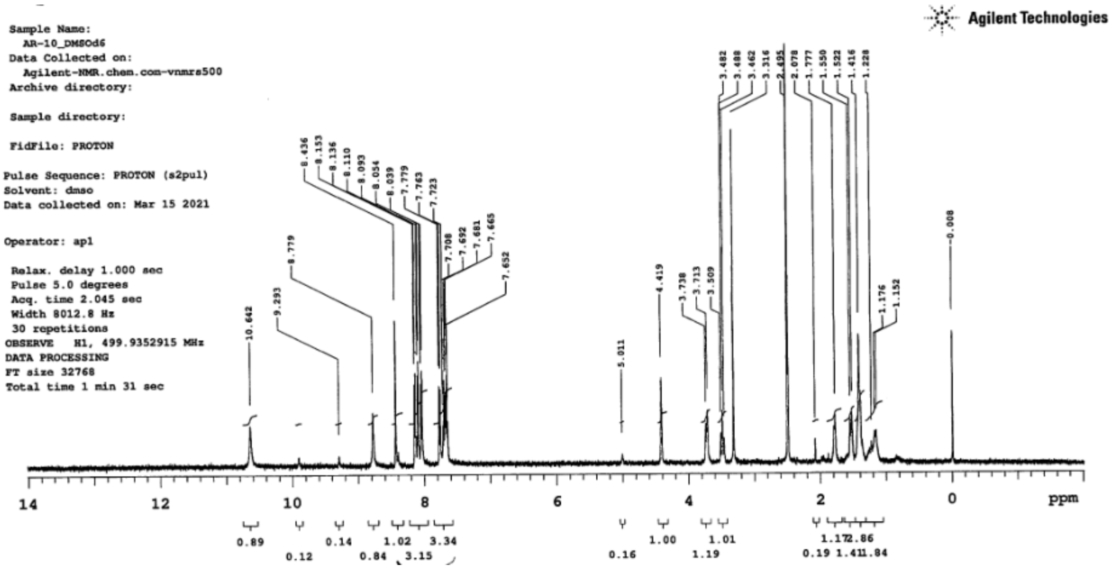

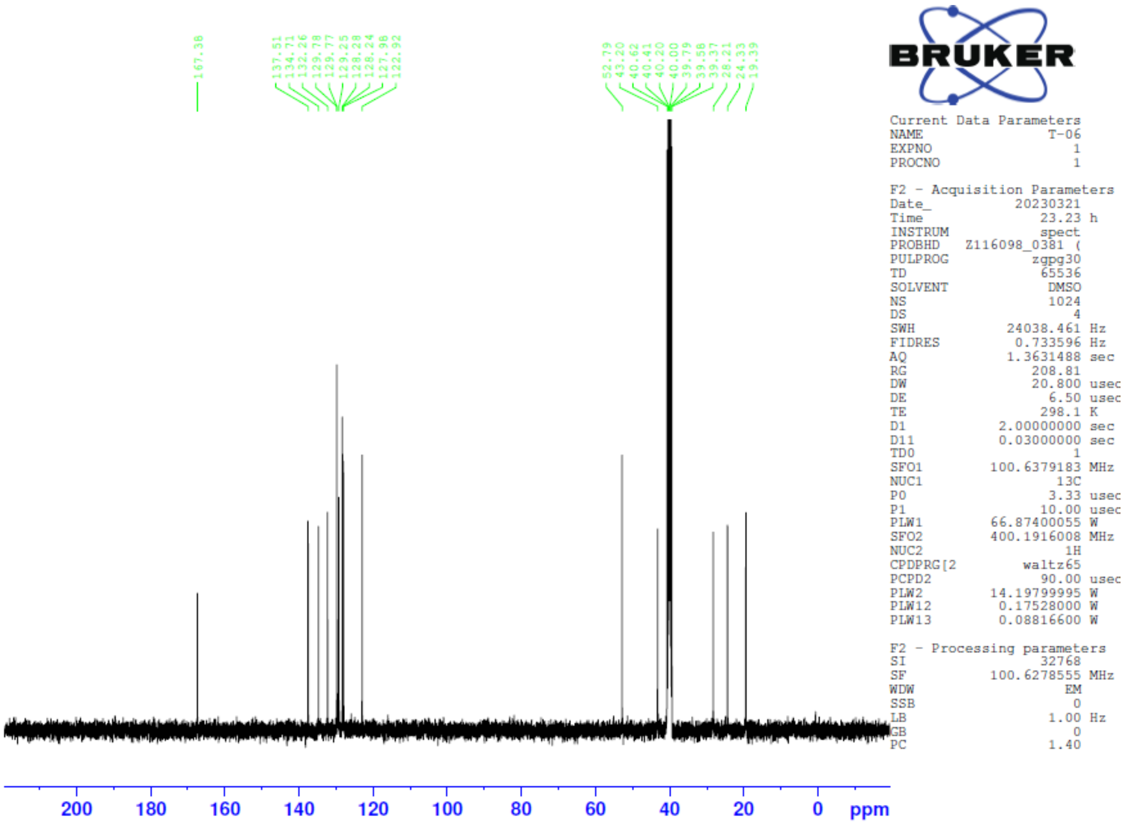

1-((4-fluorophenyl)sulfonyl)-*N*-hydroxypiperidine-2-carboxamide (1ai)

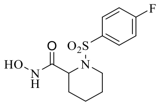

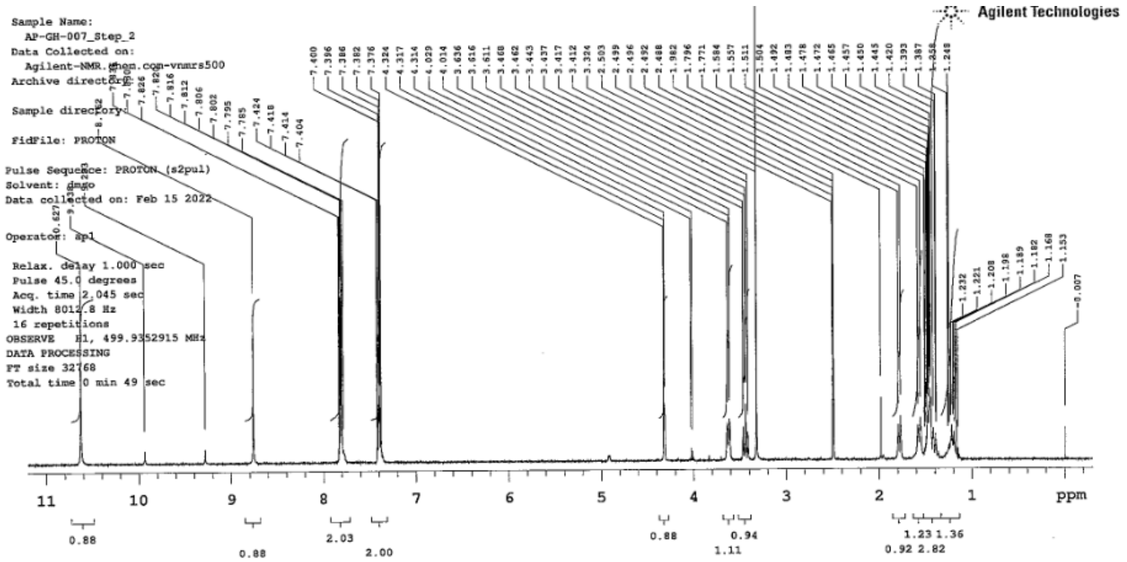

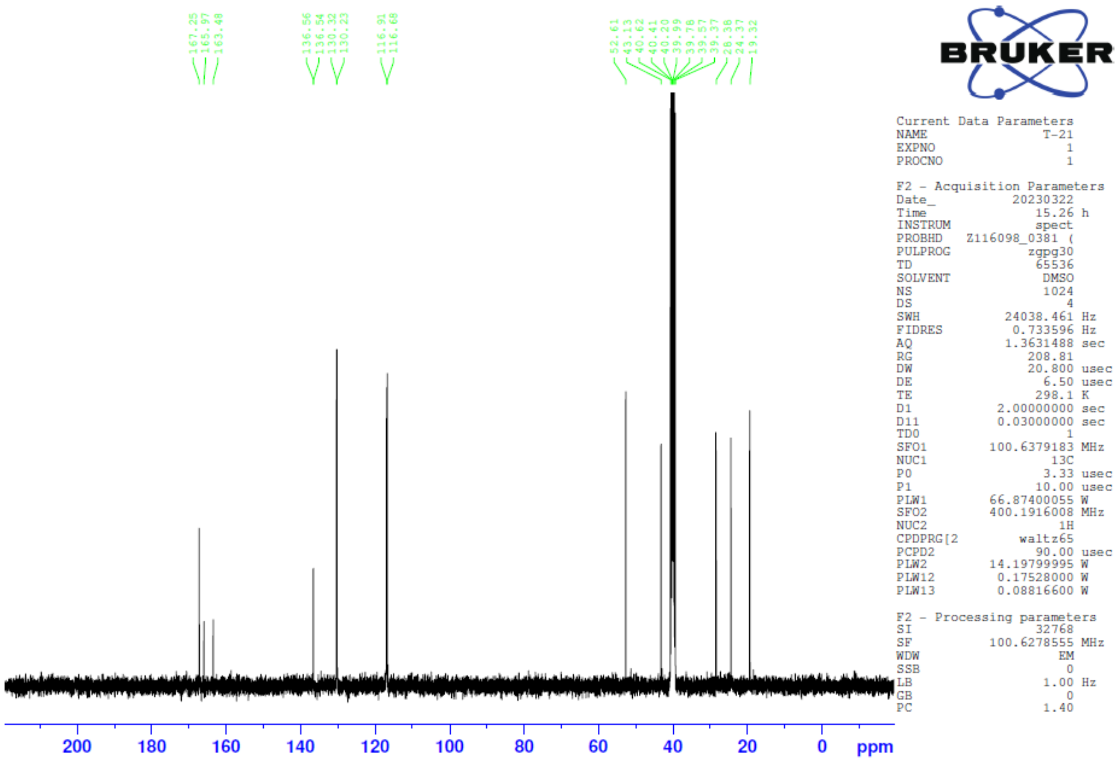

N-hydroxy-1-tosylpiperidine-2-carboxamide (1aj)

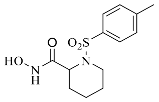

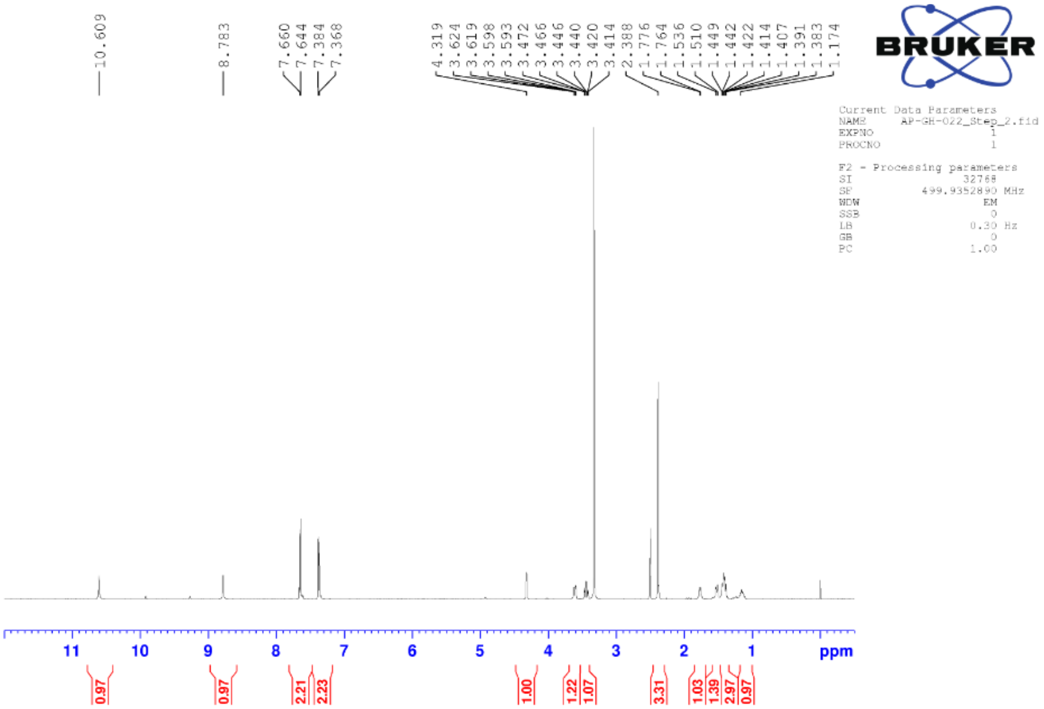

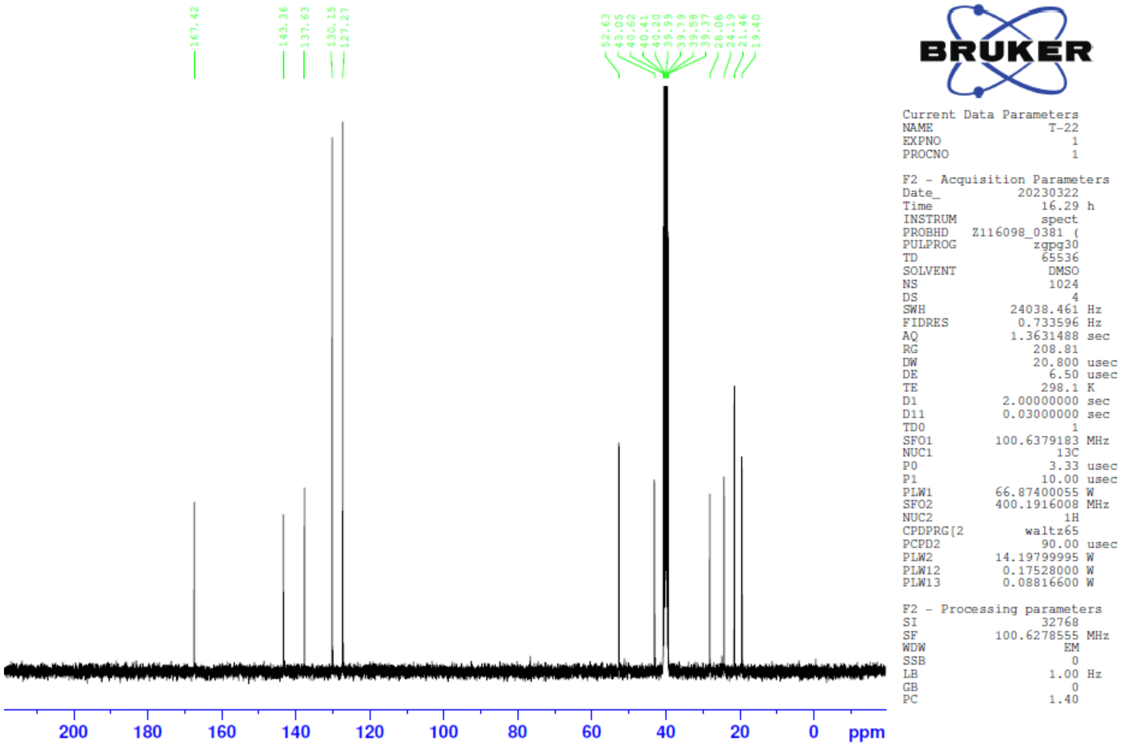

***N*-hydroxy-1-((4-propylphenyl)sulfonyl)piperidine-2-carboxamide (1ak)**

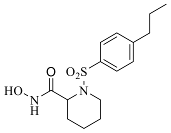

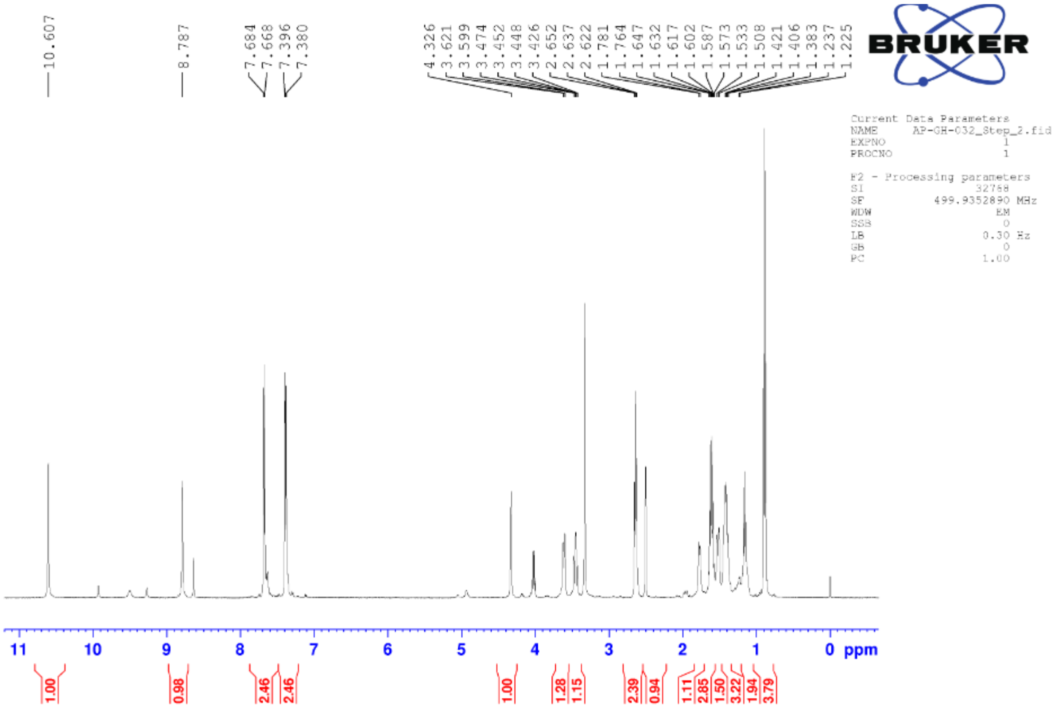

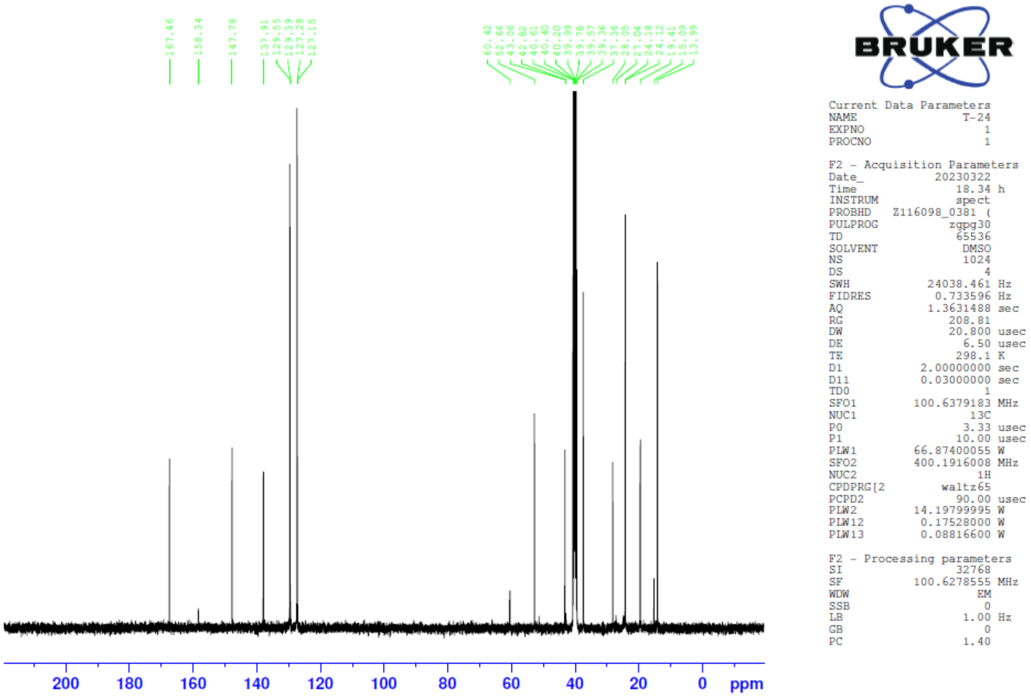

1-((4-((3*r*,5*r*,7*r*)-adamantan-1-yl)phenyl)sulfonyl)-*N*-hydroxypiperidine-2-carboxamide (1al)

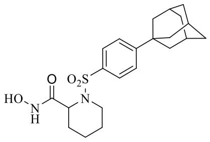

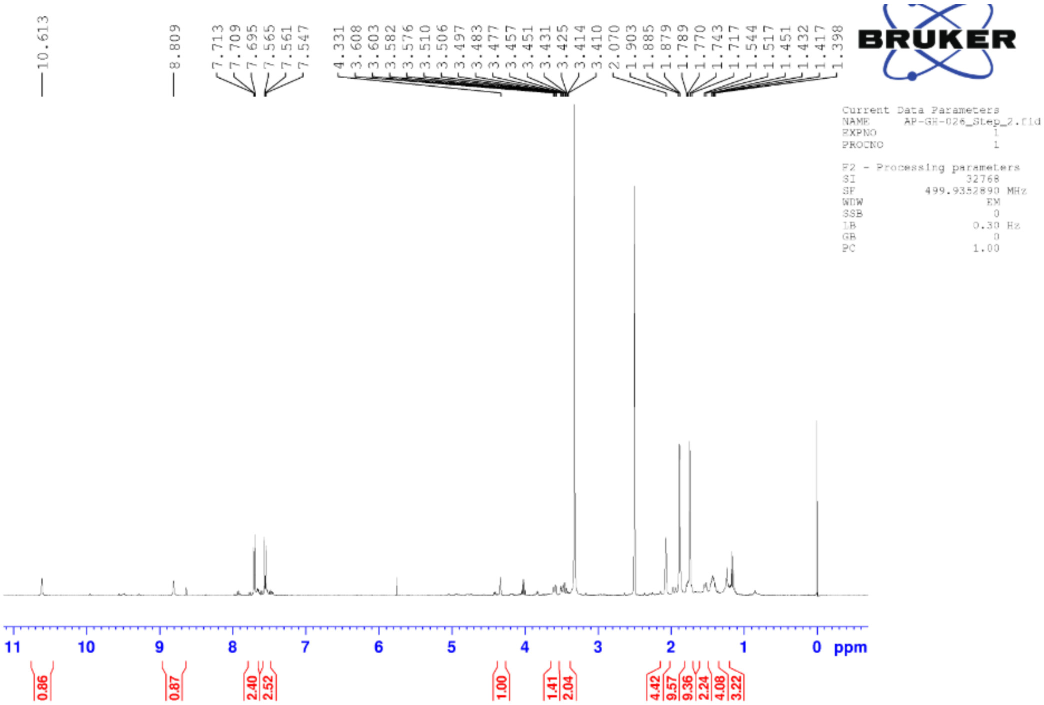

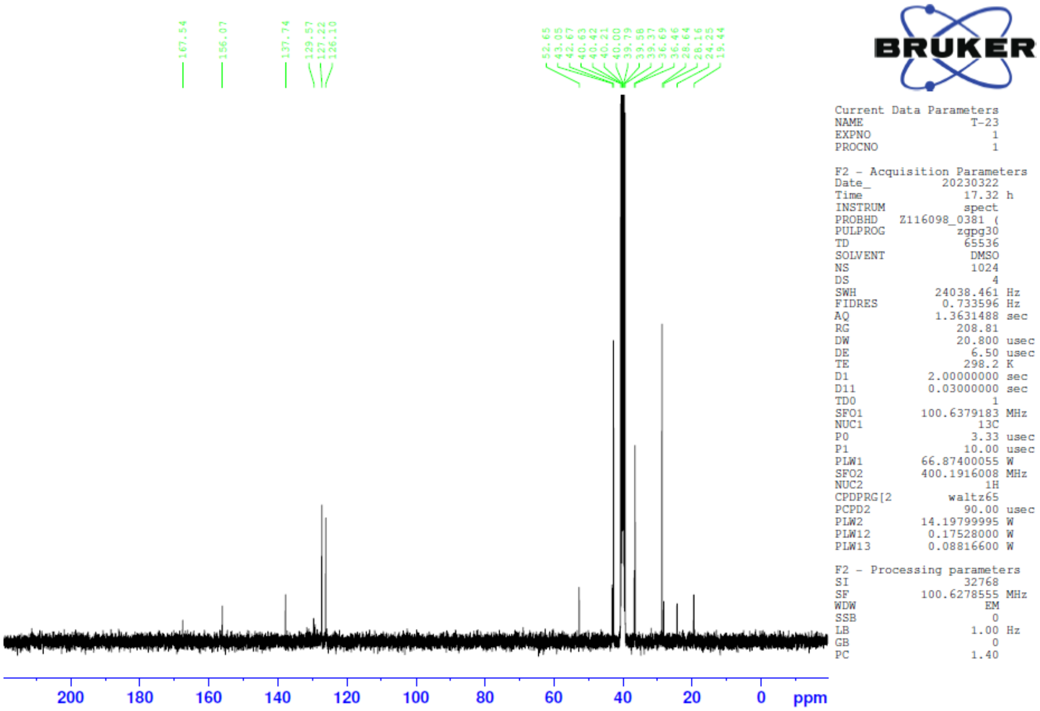

1-((4-acetamidophenyl)sulfonyl)-*N*-hydroxypiperidine-2-carboxamide (1am)

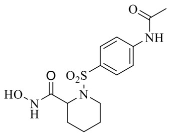

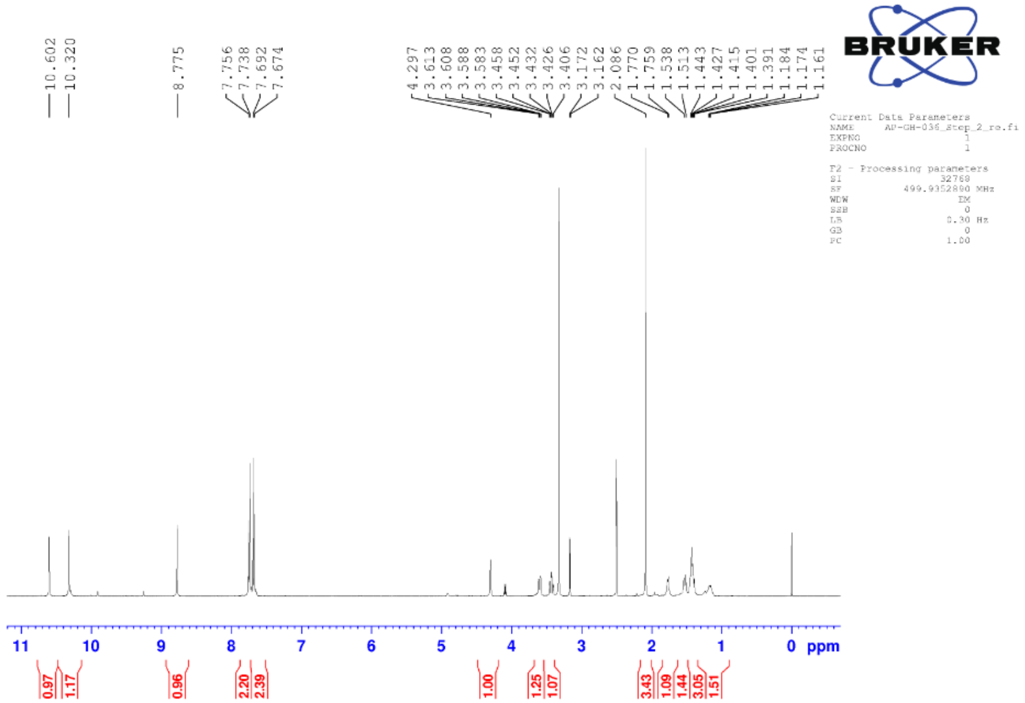

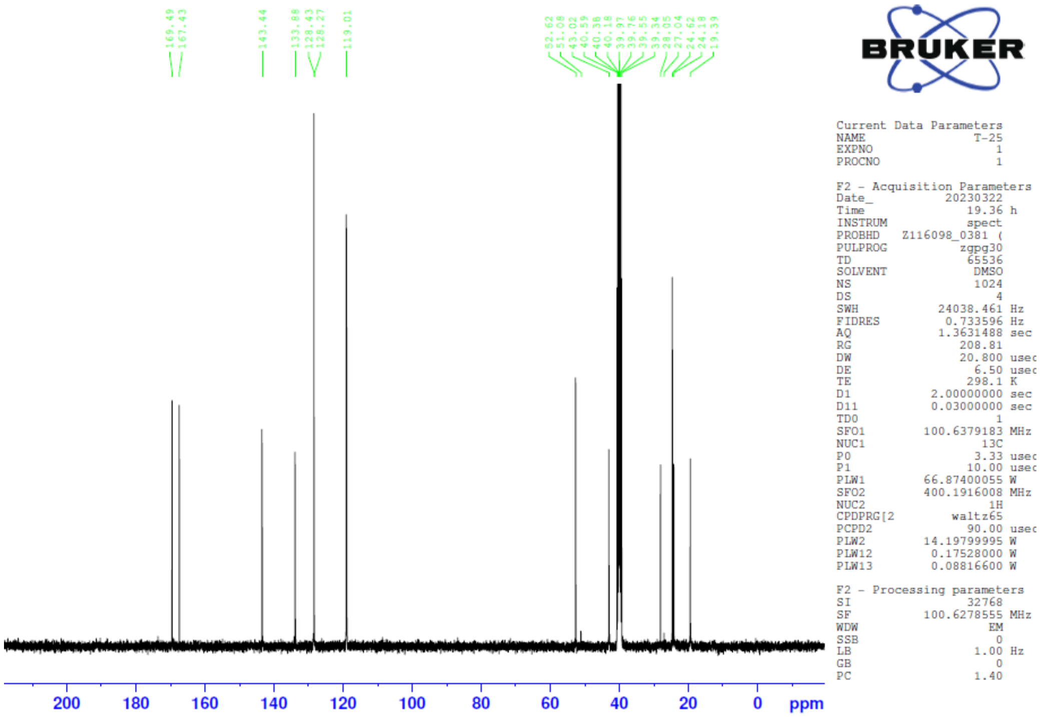

N-hydroxy-1-((4-(1-(hydroxyimino)ethyl)phenyl)sulfonyl)piperidine-2-carboxamide (4an)

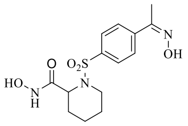

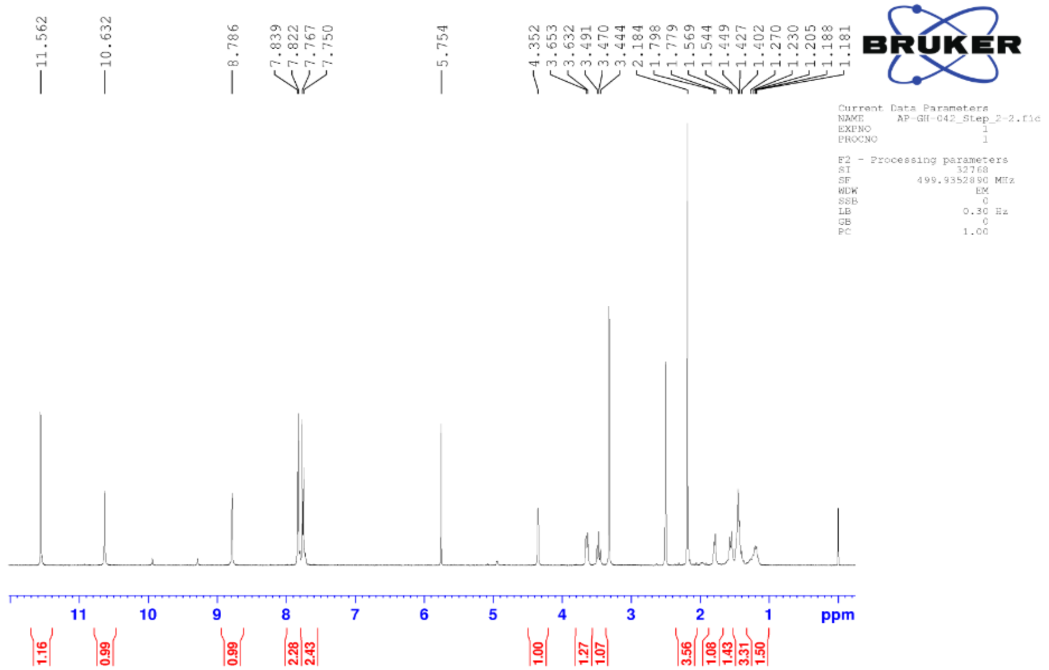

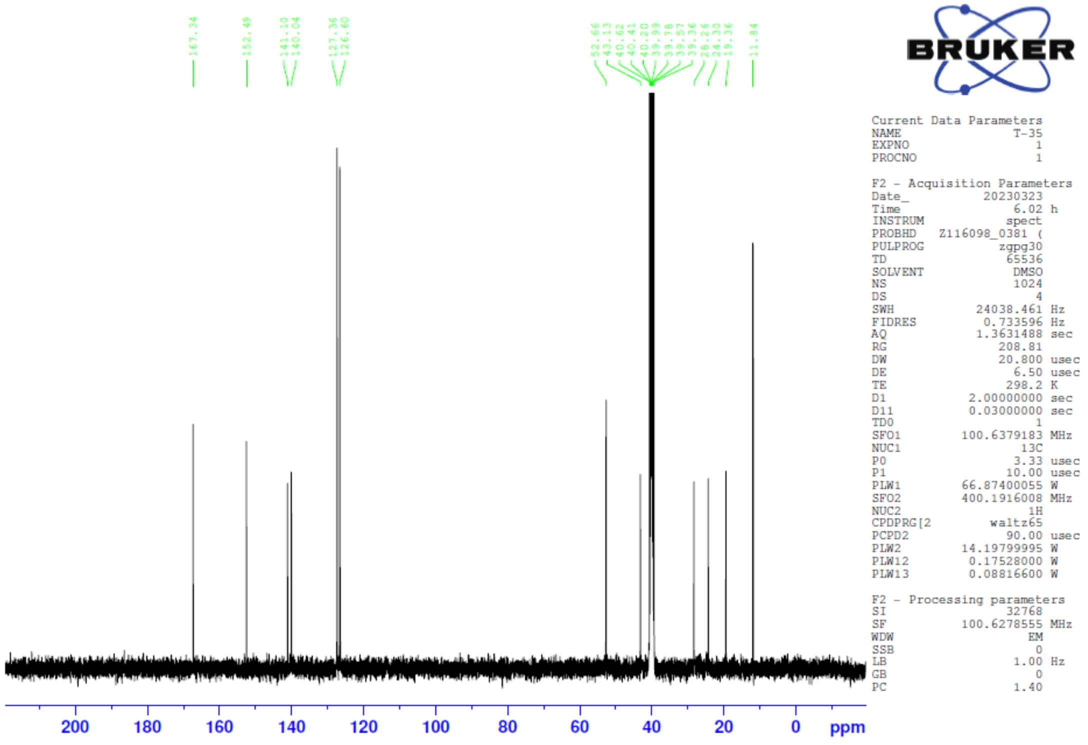

Supplement: Supplementary file 1 [file jmb-35-e2412027-supple.pdf]
